# New Pathway for Cisplatin Prodrug to Utilize Metabolic Substrate Preference to Overcome Cancer Intrinsic Resistance

**DOI:** 10.1021/acscentsci.3c00286

**Published:** 2023-07-12

**Authors:** Akil A. Kalathil, Subham Guin, Akash Ashokan, Uttara Basu, Bapurao Surnar, Katiana S. Delma, Leonor M. Lima, Oleksandr N. Kryvenko, Shanta Dhar

**Affiliations:** † NanoTherapeutics Research Laboratory, Department of Biochemistry and Molecular Biology, University of Miami Miller School of Medicine, Miami, Florida 33136, United States; ‡ Sylvester Comprehensive Cancer Centre, Miller School of Medicine, University of Miami, Miami, Florida 33136, United States; § Department of Pathology and Laboratory Medicine, Miller School of Medicine, University of Miami, Miami, Florida 33136, United States; ∥ Department of Chemistry, University of Miami, Coral Gables, Florida 33146, United States; ⊥ Department of Radiation Oncology, Miller School of Medicine, University of Miami, Miami, Florida 33136, United States; # Desai Sethi Urology Institute, Miller School of Medicine, University of Miami, Miami, Florida 33136, United States

## Abstract

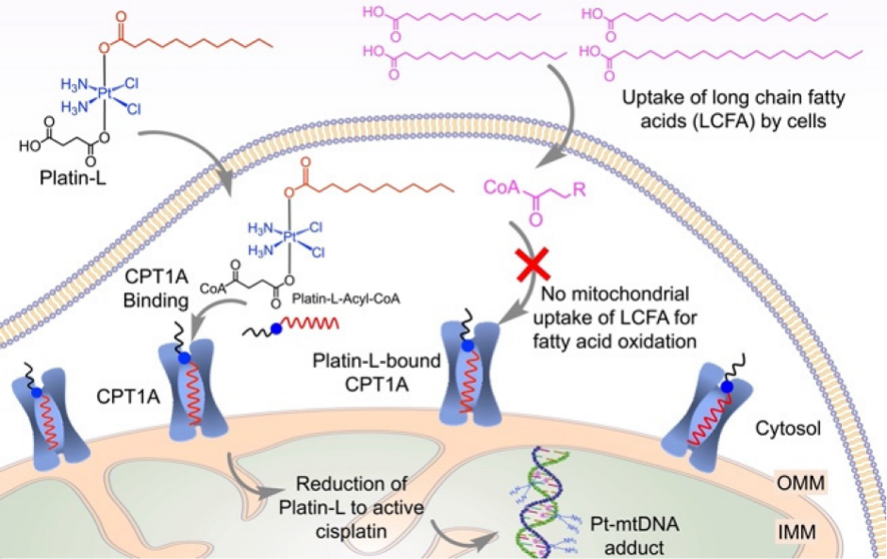

Tumor cells adapt to diverse survival strategies defying our pursuit of multimodal cancer therapy. Prostate cancer (PCa) is an example that is resistant to one of the most potent chemotherapeutics, cisplatin. PCa cells survive and proliferate using fatty acid oxidation (FAO), and the dependence on fat utilization increases as the disease progresses toward a resistant form. Using a pool of patient biopsies, we validated the expression of a key enzyme carnitine palmitoyltransferase 1 A (CPT1A) needed for fat metabolism. We then discovered that a cisplatin prodrug, Platin-L, can inhibit the FAO of PCa cells by interacting with CPT1A. Synthesizing additional cisplatin-based prodrugs, we documented that the presence of an available carboxylic acid group near the long chain fatty acid linker on the Pt(IV) center is crucial for CPT1A binding. As a result of fat metabolism disruption by Platin-L, PCa cells transition to an adaptive glucose-dependent chemosensitive state. Potential clinical translation of Platin-L will require a delivery vehicle to direct it to the prostate tumor microenvironment. Thus, we incorporated Platin-L in a biodegradable prostate tumor-targeted orally administrable nanoformulation and demonstrated its safety and efficacy. The distinctive FAO inhibitory property of Platin-L can be of potential clinical relevance as it offers the use of cisplatin for otherwise resistant cancer.

## Abstract 2

We studied how a cisplatin prodrug, Platin-L, interacts with carnitine palmitoyltransferase 1 A (CPT1A) to inhibit fatty acid oxidation and its *in vivo* efficacy using targeted nanoparticles.

## Introduction

Most malignancies use glycolysis for energy requirements to support rapid cell proliferation, growth, and metastasis.^1−3^ Prostate cancer (PCa), one of the most common types of cancers in American men,^4−6^ does not follow extensive glycolysis for transformation and growth. Plasticity in terms of substrate and pathway utilization for energy production in cancer and stromal cells is recognized as a signatory transformation, growth, and spread of PCa.^7^ Normal prostate epithelial cells use glycolysis instead of mitochondrial oxidative phosphorylation (OXPHOS) due to the impaired citrate oxidation system.^7^ On the contrary, the malignant transformation process involves the metabolic switching of cancer cells from glycolysis to efficient fatty acid oxidation (FAO) and taking advantage of the interaction with other cell types in the tumor microenvironment to force them to secrete metabolic intermediates which can be used by cancer cells.^7^ Multiple studies documented extensive use of FAO by PCa.^8−11^ Alteration of enzymes for the utilization of fatty acids (FAs) occurs in PCa relative to a normal prostate so that the transformation process can utilize the fatty acid β-oxidation pathway to support tumor growth. Enhanced FAO in prostate cancer provides both ATPs from β oxidation and ATPs from acetyl-coenzyme A (CoA) as energy sources. Mitochondrial FAO metabolism involves a circular wave of reactions utilizing long-chain fatty acids (LCFAs), medium-chain fatty acids (MCFAs), and short-chain fatty acids (SCFAs), resulting in acetyl-CoA (AcCoA) for consumption by the tricarboxylic acid (TCA) cycle. The oxidation of LCFAs which originates in the cytoplasm involves activation to produce an acyl-CoA version of the LCFAs which are then captured by the carnitine palmitoyltransferase 1 A (CPT1A), converted to LC acyl carnitine, and transported into the mitochondria for the wave of processes to participate in FAO for energy production. The shuttling of LCFAs from the cytosol to the mitochondria utilizing CPT1A is the rate-limiting step for FAO, and the presence of active CPT1A is a prerequisite for efficient FAO. However, the cells can also get limited energy by utilizing SCFAs and MCFAs which can follow a passive diffusion process across the mitochondrial membrane to feed into FAO.^12^ Thus, CPT1A is the key enzyme in the carnitine-dependent fatty acid transport across the mitochondrial inner membrane, and its deficiency results in a decreased rate of FAO.

Treatment for PCa may include surgery, radiation therapy (RT), androgen deprivation therapy (ADT),^13^ or a combination of these modalities.^14^ Most patients with high-grade PCa or diseases which poorly respond to ADT eventually demonstrate advancement of the disease. PCa, which advances in the presence of an androgen blockade, is known as castration-resistant PCa (CRPC).^4,15,16^ There is much evidence that second-generation androgen receptor (AR) antagonists, such as enzalutamide (ENZ), can be used after docetaxel or abiraterone acetate treatment^17^ to extend the lives of some CRPC patients. A wealth of new data demonstrated the beneficial effects of ENZ when combined with abiraterone acetate for CRPC patients.^18^ However, these treatment options for CRPC do not result in a cure of the disease. Under resistance and the metastatic status of CRPC, tumor cells utilize fatty acid metabolism through FAO to a greater extent to support their growth and division,^11,19,20^ and this metabolic rewiring is controlled by AR.^20−23^ In a PCa xenograft model, overexpression of the fatty acid synthase (*FASN*) gene was observed, followed by suppression of these genes under castration and again overexpression when the tumor reached the castration-resistant stage.^20^ Moreover, the transformation of both the stroma and malignant cellular network contributes to PCa advancement.^24^ In the tumor microenvironment (TME), predominantly the tumor-infiltrating myeloid-derived suppressor cells (T-MDSCs) participate in tumor growth and metastatic progression.^25^ In a PCa mouse model, it was shown that an MDSC blockage has the potential to revert chemoresistance.^26^ The prominent contributors of immunosuppressive TME, the T-MDSCs, also utilize a fatty acid substrate via an active FAO-driven metabolic pathway to fulfill their energy and acetyl-CoA requirements.^27^ Thus, the transformation of malignant and stroma cellular parts of PCa into a differentiated aggressive tumor is a multifactorial process that includes metabolic changes; both tumor and stromal immunosuppressive cells in the TME utilize FAO as a major pathway for their growth and survival.

We hypothesized that inhibition of FAO in the PCa microenvironment may be a promising strategy to sensitize these otherwise resistant cells toward chemotherapy. Our hypothesis is that the reciprocal relationship between glucose oxidation (GO) and FAO, also known as the “Randle cycle”,^28^ will play important roles under conditions where the FAO pathway is inhibited. This FAO inhibition will force the PCa cells to switch to GO to fulfill the need not only for energy but also for mitochondrial acetyl-CoA since both GO and FAO produce acetyl-CoA. This switch of metabolism to more GO will ultimately lead to decreased cell survival and increased apoptosis rates in the PCa cells. This hypothesis will work particularly well on PCa cells which utilize FAO unlike other cancer cells and immune cells which utilize glycolysis. The normal prostate cells and beneficial immune cells such as dendritic cells or T cells will not be affected by FAO inhibition since these cell populations utilize glycolysis.

Cisplatin, a FDA approved chemotherapeutic, shows extraordinary activity against testicular, breast, bladder, lung, and ovarian cancers.^29^ The efficacious anticancer activity of cisplatin arises from its ability to cause damage to nuclear DNA by forming cross-links.^30^ However, PCa is intrinsically resistant to cisplatin-based therapy.^31,32^ Thus, we also hypothesized that the development of new cisplatin analogs or prodrugs which can attack the FAO pathway can potentially provide a model scenario for cisplatin-based resistance in PCa and the ways that the resistance can be overcome.

In this report, we are summarizing, a cisplatin prodrug, Platin-L, which can (i) modulate mitochondrial metabolism and respiration of PCa cells by inhibiting FAO, forcing these populations to undergo apoptosis, (ii) show additive chemotherapeutic effects by attacking mitochondrial DNA (mtDNA) in addition to nuclear DNA (nDNA), (iii) show therapeutic effects in the PCa preclinical model, and (iv) possibly address the resistance of PCa towards cisplatin to offer the use of this prodrug in PCa. To address the clinical scenario, we incorporated Platin-L within an orally administrable prostate-specific membrane antigen (PSMA)-targeted biodegradable nanoparticle (NP) since the patient samples which demonstrate overexpression of CPT1A also express PSMA significantly.

## Results and Discussion

### Analyses of FAO-Related Genes in Patient Biopsies

**Figure 1:**
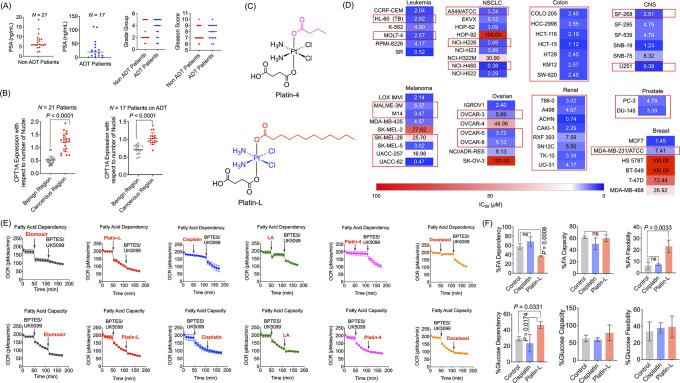
Information on patient samples and effects of Platin-L in different cell lines. Scatter plot representing the (A) PSA level, cancer grade group, and Gleason score of non-ADT-treated and ADT-treated patient samples. (B) Scatter plots indicating the relative expression of CPT1A in patient samples between benign and cancerous regions with respect to the number of nuclei. Quantification was done on the images using ImageJ software for an area containing 30 nuclei. Statistical analyses were carried out using a two-tailed paired *t* test with an α value of 0.05. (C) Structures of Platin-L and Platin-4. (D) IC_50_ values after multidose treatment with Platin-L in the NCI60 cell panel. Cell lines with dysregulated lipid metabolism are highlighted in red boxes based on the literature. Scale: max = 100 μm (deep red), mean = 14.5 μM (white), and min = 0.38 μM (deep blue). (E) Modulation of the FAO pathway in the presence of etomoxir, Platin-L, cisplatin, lauric acid, Platin-4, or docetaxel in LNCaP cells. [Etomoxir]: 40 μM; [BPTES]: 30 μM; [UK5099]: 20 μM; [all other compounds]: 100 μM. (F) Effects of Platin-L on the (upper) fatty acid dependency and (lower) glucose dependency of LNCaP cells. [Platin-L] or [cisplatin]: 10 μM for 24 h. Data are represented as an average with the standard deviation (S.D.) from three biological replicates. Statistical analyses were carried out using an ordinary one-way ANOVA analysis with an α value of 0.05.

We utilized a pool of patient biopsies from 21 new patients and 17 ADT-treated patients to understand the levels of CPT1A in the benign and cancerous regions of prostate tissue. Information about the age of the patients, prostate-specific antigen (PSA) levels, cancer grade group, and Gleason score of these samples is listed in Figure 1A and Supporting Information Figures S1A and S2A. Hematoxylin and eosin (H&E) staining of the tissue was done to visualize the cancerous region of the tissue and to understand the cancer grade demonstrating the wide range of cancer groups analyzed for this study (Supporting Information Figures S1B and S2B). These analyses revealed the histological heterogeneity of PCa as this type of cancer is known to be a heterogeneous disease.^33,34^ Given the heterogeneity on a molecular level, it is important to understand if the metabolic switch to FAO is common among different patients. To that end, immunofluorescence studies were conducted in both benign and cancerous sections of each patient sample to evaluate the difference in CPT1A expressions (Supporting Information Figure S3). A clear overexpression of CPT1A was seen in the cancerous regions compared to the benign region (Figure 1B, Supporting Information Figure S3). As a first line treatment for PCa includes ADT, we were able to analyze patient biopsies (Figure 1A and 1B and Figure S2) which had been previously treated with a form of ADT for expression of CPT1A to understand whether the treatment affected the expression. H&E staining of the samples showed the heterogeneity of the tumor (Figure S2B), indicating that the treatment does not change the morphology of the tumor. These patients have similar characteristics to the nontreated samples except for PSA levels (Figure 1A and 1B). Interestingly, the patients showed a higher PSA compared to patients which did not receive this treatment. Immunofluorescence staining for CPT1A in the benign and cancerous regions of the samples indicated that there is a higher expression of CPT1A in the cancerous regions, like that of the nontreated samples. (Figure S4A and S4B). Thus, we concluded that CPT1A can be a viable target for disrupting the FAO based metabolism for both non-ADT as well as the ADT-treated most aggressive form of PCa.

### Metabolic Alterations in PCa Cells

Normal prostate cells utilize glycolysis as their energy creation mechanism due to the impaired citrate oxidation pathway which prohibits the participation of citrate in the Krebs cycle to produce ATP.^10^ In contrast, PCa cells have highly active mitochondrial OXPHOS and fewer glycolytic patterns. We first compared the metabolic profiles, substrate utilization, and endogenous versus exogenous substrate dependence of the most used PCa cell lines, PC3 and LNCaP, to understand how metabolic modulation can nurture the tumor microenvironment. In addition to general differences between LNCaP and PC3 cells which include differential expressions of AR and PSA, LNCaP cells express both markers but PC3 cells do not. LNCaP cells are androgen-dependent and possess similar properties as observed with a majority of clinical PCa’s, while PC3 cells are androgen-independent. Our rationale to use PC3 and LNCaP was based on a recent finding by Tai et al.^35^ in which LNCaP cells have features of prostatic adenocarcinoma whereas the aggressive castration-resistant PC3 shares important features with prostatic small cell neuroendocrine carcinoma (SCNC). Thus, we wanted to explore metabolic differences, if any, in adenocarcinoma and SCNC prostate cancer types since SCNC is often seen in patients who have received hormonal therapy and develop into CRPC. We initially looked at mitochondrial OXPHOS patterns in PC3 and LNCaP cells demonstrating that in contrast to normal prostate cells, both of these PCa cell lines have active mitochondrial respiration. The extent of this respiration was more in PC3 cells as can be seen by the higher oxygen consumption rate (OCR), greater basal and maximal respiration parameters, and more intracellular ATP production (Supporting Information Figure S5A). We also observed comparatively lower glycolytic energy production by both PC3 and LNCaP cells. However, the extent of glycolysis was higher in PC3 cells compared to that observed in LNCaP cells (Supporting Information Figure S5B). We investigated the glutamine-, glucose-, and FAO-based substrate utilization by PC3 (Supporting Information Figure S5C) and LNCaP cells (Supporting Information Figure S5D) for energy production and growth. A series of fuel flex tests were carried out on these cells using the following inhibitors: UK5099, an inhibitor of plasma membrane monocarboxylate transporters and mitochondrial pyruvate carrier; etomoxir, an FAO inhibitor; and BPTES, an inhibitor of glutaminase GLS1. Addition sequence of UK5099, etomoxir, and BPTES were varied to understand the contributions from glucose, fatty acid, and glutamine, respectively.^36^ The addition sequence was varied to understand the capacity for a particular pathway. The flexibility was calculated by flexibility (%) = capacity (%) – dependency (%). First, these studies documented that both PC3 and LNCaP cells utilize FAO as a major pathway for their energy needs. Second, these experiments also demonstrated interesting differences between these two cell types; PC3 cells along with FAO also showed a dependency on glucose for mitochondrial OXPHOS, whereas LNCaP cells primarily utilize β oxidation of fatty acids for mitochondrial respiration. Furthermore, PC3 cells have the capacity for both glucose and fatty acids but flexibility only for glucose, signifying that if the glucose pathway is shut down, these cells will probably adopt more FAO oxidation pathways. On the other hand, LNCaP cells have a capacity for glucose and FAO but are flexible only for glucose, indicating that LNCaP cells fully depend on FAO and inhibiting this pathway will drive LNCaP cells toward death. We further investigated the endogenous and exogenous fatty acid substrate dependency by PC3 (Supporting Information Figure S5E) and LNCaP (Supporting Information Figure S5F) cells for FAO by utilizing the palmitate-BSA exogenous substrate. In these studies, keeping the cells in a substrate-deprived medium demonstrated lower OCRs in both cell types, and the addition of etomoxir as an inhibitor of FAO abolished this OCR, demonstrating that the respiration was FAO-driven and both PC3 and LNCaP cells utilize an endogenous fatty acid for FAO. Addition of the palmitate-BSA substrate demonstrated an enhancement in OCR, basal and maximal respiration parameters, and ATP production only in LNCaP cells but not in PC3 cells, implying that LNCaP cells utilize both an endogenous and exogenous fatty acid substrate for FAO-driven respiration but PC3 cells utilize only an endogenous substrate. In these experiments, we used a MitoStress kit with FCCP to make sure that the increase in oxygen consumption is due to FAO and mitochondria remaining coupled since free fatty acids are weak lipophilic acids and can mildly uncouple mitochondria.

We also studied the metabolic profile of two additional PCa cell lines: DU145, which is androgen-independent and insensitive, and 22RV1, which is androgen-independent but sensitive. Both DU145 and 22RV1 were found to rely mostly on fatty acid and glucose for their growth and proliferation, like PC3 but unlike LNCaP cells that were solely dependent on fatty acids (Supporting Information Figure S6). Nearly 60% dependency on FA was observed for DU145 and 22RV1 cells and 20–30% dependency on glucose was observed while the dependency on glutamine was nominal for all of the cell lines except DU145 cells, which also had nearly 20% glutamine dependency. These studies confirmed the utilization of FA by the PCa cells and allowed us to probe the activity of Platin-L in these cells to overcome cisplatin resistance.

### Modulation of Fatty Acid Oxidation-Based Bioenergetics in PCa Cells by Platin-L

Our observations that PCa cells mainly rely on FAO for energy production and other literature reported studies^37−39^ led us to hypothesize that the inability of existing Pt-based compounds to modulate FAO-based energy production in PCa cells might contribute to the resistance. In our continuing effort to find why PCa is resistant to cisplatin-based drugs, we screened several cisplatin prodrugs along with cisplatin and oxaliplatin to determine if any of these compounds can inhibit mitochondrial OXPHOS utilizing fatty acids in PCa cells. We discovered by serendipity that Platin-L (Figure 1C), a prodrug of cisplatin with a borderline long-chain fatty acid, lauric acid, can inhibit the FAO oxidation pathway in PCa cells to a similar extent as shown by etomoxir, an irreversible CPT1A inhibitor. In this work, we set out a series of mechanistic studies to understand the structural aspects which allow Platin-L to efficiently inhibit FAO. For a brief note, Pt(IV) prodrugs offer greater kinetic inertness^40^ and the biological activity of Pt(IV) prodrugs involves the intracellular reduction and release of active cisplatin to have its effect on nuclear DNA. Our first hypothesis why Platin-L might be inhibiting FAO comes from the fact that the lauric acid can probably interact with the hydrophobic area of CPT1A and thus interrupt the streaming of LCFAs into the mitochondria, resulting in the inhibition of FAO. To understand the mechanism, we created another Pt(IV) prodrug, Platin-4^41,42^ with an SCFA linker butyrate (Figure 1C). Both Platin-L and Platin-4 contain succinate as the other axial ligand in the octahedral Pt(IV) coordination sites. We previously called Platin-L as Platin-12.^42^ A recent study documented that butyrate could enhance CPT1A activity to promote FAO,^43^ thus we hypothesized that Platin-4 would be valuable to understanding and discovering the activity of Platin-L. Over the past few years, we actively addressed various facets to develop new cisplatin prodrugs which can show activity in PCa. These efforts resulted in several prodrugs: Platin-A,^44^ formulated using cisplatin and aspirin; Platin-B,^45^ a combination of cisplatin and a potent alkylating agent mimicking pipobroman; Platin-Az,^46^ synthesized as a tool to help us incorporate additional functionalities with ease; and Platin*-*M,^47^ a Pt-prodrug to detour cisplatin to avail the mitochondrial repair-resistant DNA pool to exert its therapeutic effect. We screened all of these Pt(IV) prodrugs along with docetaxel, oxaliplatin, and cisplatin to understand the effects of the Pt center on FAO inhibition (Supporting Information Figure S7). We documented that in addition to Platin-L, only Platin-Az, Platin-B, and Platin-M have some activity toward FAO. From a comparison of the structures of these Pt(IV) compounds, we believe that long alkyl chains might be playing a role in inducing FAO inhibitory properties through competitive binding to the active site of CPT1A. When Platin-L was challenged on the NCI60 cell panel via a single dose of 10 μM, it showed a remarkable effect at reducing the overall growth by more than 50% in several cell lines (Supporting Information Figure S8). Additionally, when the cell lines were challenged on a multidose regimen, several of the same cell lines had low IC_50_ values, indicative of the cytotoxicity of Platin-L (Figure 1D). Several of the cell lines or cancer types in the panel have dysregulated lipid metabolism. The aberrant expression of genes required for fatty acid synthesis, metabolism, and/or oxidation were reported for the cell lines indicated in red boxes in Figure 1D and Supporting Information Figure S8.^48−57^ Together with the previous metabolic studies done on PC3, LNCaP, 22RV1, and DU145 cells with Platin-L, these data indicated that a potent FAO inhibitor would be capable of inhibiting cancer growth in not only PCa but also different cancers which have aberrant FAO expression. When compared to the single-dose growth inhibition effect that cisplatin has on the same NCI-60 panel, across the board Platin-L shows more inhibition, indicative of its increased potency (DTP database NSC number 119875). This opens the possibility of treating cancers which are classically cisplatin-resistant with a cisplatin prodrug.

We then conducted FA dependency studies in LNCaP cells using cisplatin, docetaxel, Platin-4, Platin-L, and etomoxir, an irreversible inhibitor of CPT1A. First, to optimize the working concentration of Platin-L, a concentration-dependent study in LNCaP cells with 50, 100, and 200 μM Platin-L was conducted (Supporting Information Figure S9). It was noted that 50 μM is not effective at causing significant changes in OCR while 200 μM causes a very sharp change in OCR of the cells due to its immense cytotoxicity. In our next experiment, we compared the activity of Platin-L with the well-known CPT1A inhibitor, etomoxir. A sharp drop in the OCR from the basal level of LNCaP cells was observed when Platin-L was injected, and the OCR level did not change significantly after subsequent additions of glucose and glutamine metabolic inhibitors, UK5099 and BPTES, respectively (Figure 1E). The drop in OCR after the Platin-L port injection was like that when etomoxir was used (Figure 1E). Under the same experimental settings, injection of cisplatin, lauric acid (LA), Platin-4, or docetaxel did not demonstrate such an OCR drop (Figure 1E). It was seen that when etomoxir was injected first into LNCaP cells, the OCR dropped from 210 to 130 pmol/min as expected due to the high dependency of LNCaP cells on FAs. Similar changes in OCR were not observed with the addition of cisplatin, Platin-4, or docetaxel. The injection of lauric acid alone or in combination with cisplatin induced moderate changes in the OCR. Maximum lowering of OCR like that induced by etomoxir was observed with the injection of Platin-L. The cells where etomoxir or Platin-L was injected first did not show further significant OCR changes upon addition of UK5099 and BPTES whereas those where cisplatin, lauric acid (LA), a mixture of cisplatin and LA, or docetaxel was first injected showed a moderate drop in OCR after UK5099 and BPTES addition (Figure 1E). The addition of UK5099 or BPTES to the cells did not result in pronounced OCR changes while subsequent addition of Platin-L caused sharp drops in OCR values (Figure 1E bottom). Similar trends were observed when these experiments were carried out in PC3 cells (Supporting Information Figure S10). These studies demonstrated that Platin-L might be interacting with CPT1A due to the structural similarity with essential long-chain FAs.

When LNCaP cells were pretreated with Platin-L, these cells showed a reduced dependence on FAs (Figure 1F). In these experiments, the cells were incubated with Platin-L or cisplatin for 24 h and the fatty acid dependency was measured by recording the basal OCR and that after adding CPT1A inhibitor, etomoxir, and subsequently glucose and glutamine pathway inhibitors, UK5099 and BPTES, respectively (Figure 1F, Supporting Information Figure S11). The difference in basal OCR and that after the addition of the target inhibitor divided by the difference in basal OCR and that after the addition of the other two inhibitors gave the dependency on the fuel pathway. It was noted that the cells pretreated with Platin-L were less responsive toward etomoxir injection compared to cells alone or those pretreated with cisplatin. The FA dependency in LNCaP cells decreased from 80 to 30–40% in cells pretreated with Platin-L. Furthermore, this inhibition of fatty acid dependency of PCa cells by Platin-L was accompanied by an enhancement of the cells’ glucose dependency (Figure 1F). These results indicated that Platin-L has the potential to perform a metabolic switching of PCa cells from FAO to GO. As a result of these metabolic inhibitions by Platin-L, we observed overall reductions in total ATP production in both LNCaP and PC3 cells (Supporting Information Figure S12).

### Platin-L Binding to CPT1A

**Figure 2:**
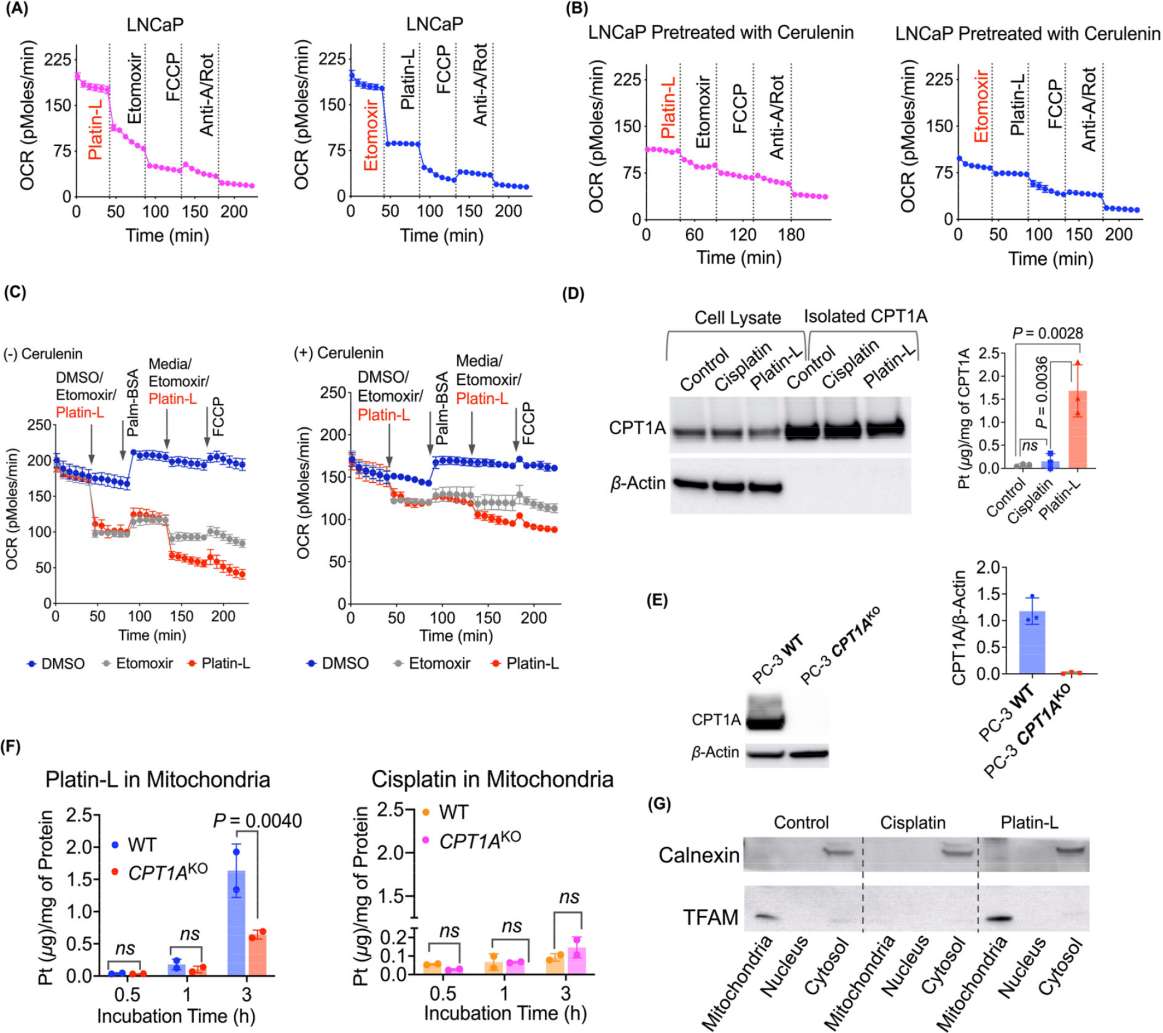
Interaction of Platin-L with CPT1A and its entry into the mitochondria. LNCaP cells (A) without cerulenin pretreatment and (B) with cerulenin pretreatment followed by etomoxir or Platin-L to check FAO inhibition efficacy. (C) OCR of Platin-L- or etomoxir-treated cells in the presence of palm:BSA with or without prior treatment with cerulenin. [Platin-L]: 10 μM for 24 h; [cerulenin]: 20 μM for 24 h. The concentration of free fatty acid [FFA] from palm-BSA was ∼4.3 nM. (D) CPT1A expression in cell lysate and immunoprecipitated CPT1A samples for control, cisplatin-, and Platin-L-treated groups for 6 h in PC3 cells by Western blot and the amount of Pt present in those immunoprecipitated CPT1A groups ([platin-L]: 50 μM and [cisplatin]: 50 μM). Statistical analysis was carried out using ordinary one-way ANOVA analyses with an α value of 0.05. (E) Validation of CRISPR/Cas9-based knockout of the *CPT1A* gene in PC3 cells using Western blot data. (F) Amount of Pt present in the mitochondrial fraction of Platin-L- and cisplatin-treated PC3 cells at different time intervals with respect to protein. [Platin-L]: 50 μg/mL and [cisplatin]: 50 μg/mL. Statistical analyses were carried out using a two-way ANOVA analysis with an α value of 0.05. (G) Expressions of calnexin and TFAM in nuclear, mitochondrial, and cytosolic fractions of the control, cisplatin-, and Platin-L-treated PC3 cells. [Platin-L]: 50 μg/mL and [cisplatin]: 50 μg/mL.

We next assessed the effects of etomoxir followed by the injection of either Platin-4 or Platin-L and *vice versa* in LNCaP cells to shed light on whether the cells respond to the injection of Platin-L once etomoxir partially inhibits the activity of CPT1A (Figure 2A, Supporting Information Figure S13). The rationale behind this study was if Platin-L interacts with the CPT1A binding pocket then the cells would not show sensitivity to subsequent etomoxir addition. It was noted that once etomoxir is injected, the cells do not show any further lowering of OCR with the addition of Platin-4. The injection of Platin-L, however, attenuated the OCR of the cells (Figure 2A, Supporting Information Figure S13). Likewise, when Platin-L was first injected, the cells showed very little changes in the OCR with etomoxir injection (Figure 2A). This series of experiments indicated that Platin-L might be interacting with the CPT1A binding pocket.

Cerulenin is an antifungal antibiotic that is known to inhibit fatty acid and steroid biosynthesis. In FA synthesis, it binds to β-keto-acyl-ACP synthase, one of the seven domains of FASN, blocking the interaction of malonyl-CoA. LNCaP cells were pretreated with cerulenin, and OCR changes were monitored with sequential injections of etomoxir and Platin-4, etomoxir and Platin-L, or the reverse (Figure 2B, Supporting Information Figure S13). The two notable observations were (i) the basal OCR of the cells was lower by ∼100 pmol/min compared to the cells without cerulenin and (ii) the cells were significantly less responsive to the injection of CPT1A inhibitor etomoxir or Platin-L. This implied that since FASN is inhibited, there is a deficit of FAs and hence binding to CPT1A does not alter its metabolic pattern. This observation is also in accordance with the FA dependency and FAO ability of LNCaP cells. When glucose and glutamine metabolic inhibitors, UK5099 and BPTES, respectively, were injected first followed by the subsequent injection of Platin-L, cisplatin, or Platin-4; only Platin-L demonstrated a drop in OCR in LNCaP cells, further confirming that the respiration due to substrates other than glucose or glutamine is inhibited by Platin-L. Cisplatin or Platin-4 did not have such an inhibitory property (Supporting Information Figure S14).

We also assessed actions of etomoxir or Platin-L in the presence of an exogenous FA substrate, palmitate-BSA conjugate or palm-BSA, in LNCaP cells (Figure 2C). The first injection of etomoxir or Platin-L caused a sharp drop in the OCR of LNCaP cells, but the injection of palm-BSA could partially recover the basal OCR. After the second addition of the inhibitors, the cells did not respond anymore to the externally provided substrate. Injection of the vehicle alone (DMSO) did not show any changes in the OCR patterns of the cells (Figure 2C). All of these observations further corroborate our hypothesis that Platin-L acts on CPT1A like etomoxir does via a competitive binding due to its structural similarity with long-chain FAs which is recognized by the FA transporters on the outer mitochondrial membrane (OMM).

We extended our studies further to understand how Platin-L works on CPT1A and the consequences for mitochondrial FAO. Our attempt to isolate CPT1A from Platin-L-treated PC3 cells by immunoprecipitation of CPT1A and quantification of Platin-L in isolated protein by ICP-MS indicated that Platin-L remains bound to CPT1A, but cisplatin did not show such binding toward this protein (Figure 2D). We probed this further by creating *CPT1A*^KO^ PC3 cells using CRISPR-cas9 technology (Figure 2E, Supporting Information Figure S15), and a time-dependent mitochondrial localization study revealed that the overall uptake of Platin-L in the mitochondria is significantly less in *CPT1A*^KO^ cells than in the wild type PC3^WT^ cells (Figure 2F). However, the uptake of Platin-L in the nuclear fractions was not different between WT and *CPT1A*^KO^ cells (Supporting Information, Figure S16). The purity of different cellular fractions was confirmed by Western blot using TFAM as mitochondrial marker and Calnexin as cytosol marker (Figure 2G). Thus, we believe that Platin-L utilizes the lauric acid arm to become associated with CPT1A and then internalizes inside the mitochondria.

### Possible Mechanism of Action of Platin-L

**Figure 3:**
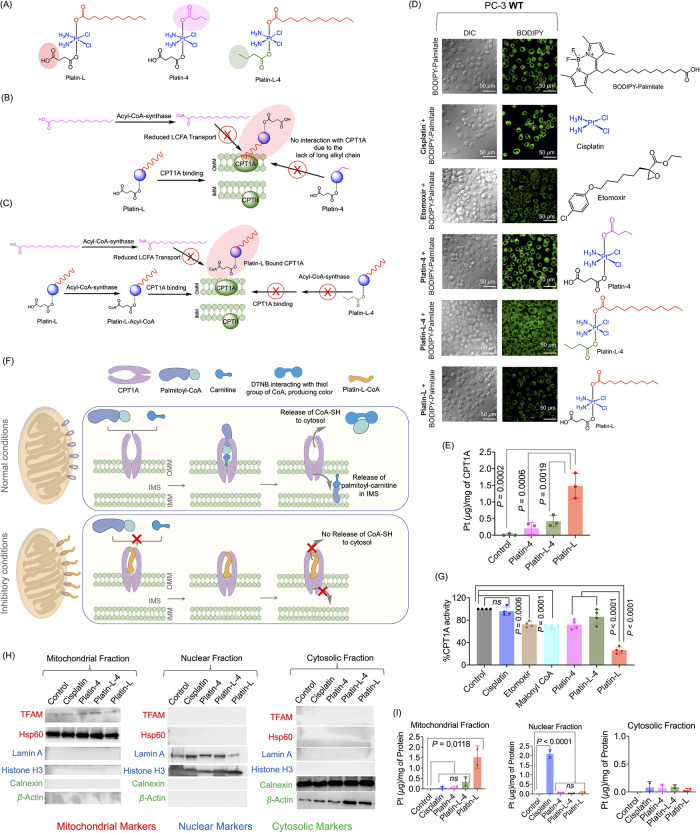
Effects of axial ligands on CPT1A activity. (A) Structures of Platinum(IV) prodrugs with variation at the axial ligands for understanding the role of these ligands in CPT1A binding. Schematic representation of (B) Platin-L binding to CPT1A due to the long-chain fatty acid, compared to Platin-4 which does not bind due to the short-chain fatty acid and (C) Platin-L binding to CPT1A due to its ability to interact with acyl-CoA synthase to become Platin-L-Acyl-CoA and interact with CPT1A compared to Platin-L-4, which does not have the ability to convert to an acyl-CoA form. (D) Incorporation of fluorescent, long-chain fatty acid BODIPY-palmitate into PC3 cells after treatment with various compounds using live cell imaging. (E) Immunoprecipitation (IP) of CPT1A to quantify the amount of Platinum present on CPT1A after treatment of PC3 cells with Platin-4, Platin-L-4, or Platin-L. The data presented here is an average with the standard deviation from three independent biological replicates (*N* = 3). (F) Schematic representation of the CPT1A activity assay. Top: normal conditions for CPT1A function, resulting in the release of CoA-SH into the cytosol. Bottom: inhibitory conditions, where CPT1A is inhibited and CoA-SH is not released into the cytosol. The amount of CoA-SH released can be directly correlated to the activity of CPT1A. (G) Relative activity of CPT1A after treatment with test articles at 10 μM. The data presented here is an average with the standard deviation from four independent biological replicates (*N* = 4), each with three technical replicates (*n* = 3). Ordinary one-way ANOVA was used to calculate the significance. (H) Western blot analyses of the subcellular fractions used for Pt quantification to ensure purity. (I) Platinum content in subcellular fractions by ICP-MS. The data presented is the mean with the standard deviation from two independent biological replicates. Ordinary one-way ANOVA with multiple comparisons was used to calculate the significance.

We probed the binding ability of Platin-L to CPT1A by exploring the structure–activity relationship. We hypothesized that perhaps CPT1A binding of Platin-L causes competition toward LCFA uptake to the mitochondria for oxidation, and hence we see fatty acid oxidation inhibition by Platin-L. To this end, we synthesized and characterized a new compound, Platin-L-4 (Figure 3A, Supporting Information Figure S17). We utilized Platin-4, Platin-L, and Platin-L-4 to probe our hypotheses on two fronts (Figure 3A). Our first hypothesis included that if CPT1A binding by Platin-L was facilitated by the long lauric acid arm then Platin-4 lacking such an arm will not result in CPT1A and FAO inhibition (Figure 3B). However, we also wanted to understand the effects which the succinate arm can have on these activities. We questioned whether the Pt-bound succinate can be activated by acyl-coA-synthase to form acyl-CoA, which then helps Platin-L to bind CPT1A more efficiently (Figure 3C). This could take place if the carboxylic acid group on the succinate arm causes the molecule to be recognized as a fatty acid and is then shuttled to the mitochondria, preventing Platin-L from being reduced to cisplatin. To probe these, we used BODIPY-labeled palmitate. We treated cells with cisplatin, etomoxir (positive control), Platin-4, Platin-L-4, or Platin-L followed by the addition of BODIPY-palmitate and observed that only pretreatment with etomoxir or Platin-L causes inhibition in the uptake of palmitate, further confirming that Platin-L binds to CPT1A (Figure 3D). The reduction of BODIPY-palmitate uptake in the cells after treatment with Platin-L could be due to the inability of the cells to process the long-chain fatty acid since Platin-L interacts with CPT1A, preventing the relocation of BODIPY-palmitate to the mitochondria. The cells may break down the long-chain fatty acid through other mechanisms or efflux out the excess. The lack of inhibition by Platin-L-4 in palmitate uptake confirmed that the succinate arm is important for initial acylation to occur for recognition by CPT1A followed by binding of the lauric arm. These studies were further confirmed by conducting experiments in *CPT1A*^KO^ cells. Overall, we observed no appreciative uptake of palmitate in these cells (Supporting Information Figure S18). We also observed that in *CPT1A*^KO^ cells the overall uptake of BODIPY-palmitate is less, and this can be due to some other changes such as the reduced expression of fatty acid transporter CD36 and the subsequent need for a longer incubation period with the long-chain fatty acids. We further confirmed this structure–activity relationship by comparing Pt-bound CPT1A by isolating CPT1A from PC-3 cells treated with Platin-L, Platin-4, or Platin-L-4 and quantifying Pt in the immunoprecipitated protein by ICP-MS (Figure 3E, Supporting Information Figure S19 for the purity of CPT1A by Western blot). Our analyses further confirmed that only Platin-L binds to CPT1A significantly compared to Platin-4 or Platin-L-4.

### Effects of Axial Ligands on CPT1A Activity

The effect of Platin-L on CPT1A activity was measured by quantifying the amount of CoA-SH released during the normal functioning of FAO-dependent cells.^43,58^ Ellman’s reagent, 5,5-dithio-bis(2-nitrobenzoic acid) (DTNB), was used to react with the free thiol group of CoA-SH. When CPT1A interacts with fatty acids, such as palmitoyl-CoA in the presence of fatty acid transporter quaternary ammonium compound carnitine, it converts the fatty acid to the carnitine version, palmitoyl-carnitine, and releases CoA-SH into the cytosol and palmitoyl-carnitine into the intermembrane space of the mitochondria (Figure 3F). To investigate the extent to which Platin-L inhibits CPT1A, we treated PC-3 cells with Platin-L and then collected the lysate to evaluate the CPT1A activity. The lysates were treated with palmitoyl-CoA and L-carnitine. After 3 h, DTNB was added to interact with the thiols, and the absorbance values were recorded on a plate reader. Platin-L showed significantly less CoA-SH compared to the controls, indicating that Platin-L prevented CPT1A from executing its primary function. The data also showed that Platin-L has a direct impact on CPT1A and mechanistically inhibits it from transporting long-chain fatty acids into the mitochondria, prohibiting fatty acid oxidation from occurring (Figure 3G, Supporting Information Figure S20 for four independent sets of experiments along with images of original plates with color changes). The known CPT1A inhibitor, etomoxir, had a dose-dependent effect on the activity (Figure 3G, Supporting Information Figure S20). Similarly, malonyl-CoA, an allosteric inhibitor of CPT1A, has a negative effect on CPT1A activity (Figure 3G, Supporting Information Figure S20). Cisplatin and Platin-L-4 had no effect on CPT1A activity, and Platin-4 had a slight effect (Figure 3G, Supporting Information Figure S20). However, the CPT1A inhibitory effects of Platin-L were significantly higher than those for Platin-4 and Platin-L-4 (Figure 3G, Supporting Information Figure S20), reinforcing our previous conclusions that a succinic acid arm and a long-chain fatty acid-based ligand are necessary for Platinum compounds to interact with CPT1A. Our observations were further confirmed by a quantitative comparison of the transport of the Pt(IV) prodrugs to the mitochondria via CPT1A binding. Treatment of PC3 cells with Platin-4, Platin-L-4, or Platin-L and subsequent isolation of mitochondrial, nuclear, and cytosolic components and debris were conducted. The purity of each of these components was confirmed by using several markers for each of these components (Figure 3H, Supporting Information Figure S22 for two independent sets of experiments). Differences in the lipophilicity of Platin-4, Platin-L-4, and Platin-L^42^ contribute to differences in overall cellular uptake kinetics of the molecules (Supporting Information Figure S21). The quantification of Pt in each of these components by ICP-MS indicated transport of only Platin-L to the mitochondria compared to Platin-4 or Platin-L-4 due to the binding of Platin-L to the CPT1A (Figure 3I, Figure S22 for two independent sets of experiments). The difference in the accumulation of Pt by treatment with either cisplatin or Platin-L indicated that Platin-L is able to reach the mitochondria without undergoing cytosolic reduction to Pt(II) (Figure 3I). This is further supported by the effect of Platin-L in preventing long-chain BODIPY-palmitate from entering the mitochondria (Figure 3D). We would like to add that lauric acid is typically regarded as a medium -chain fatty acid^12^ and normally would not require the use of CPT1A to enter the mitochondria. However, in Platin-L, given the addition of cisplatin and succinic acid, the overall length of Platin-L may resemble that of a long-chain fatty acid which would allow it to be taken up by CPT1A.

### Orally Administrable Targeted Platin-L NPs

**Figure 4:**
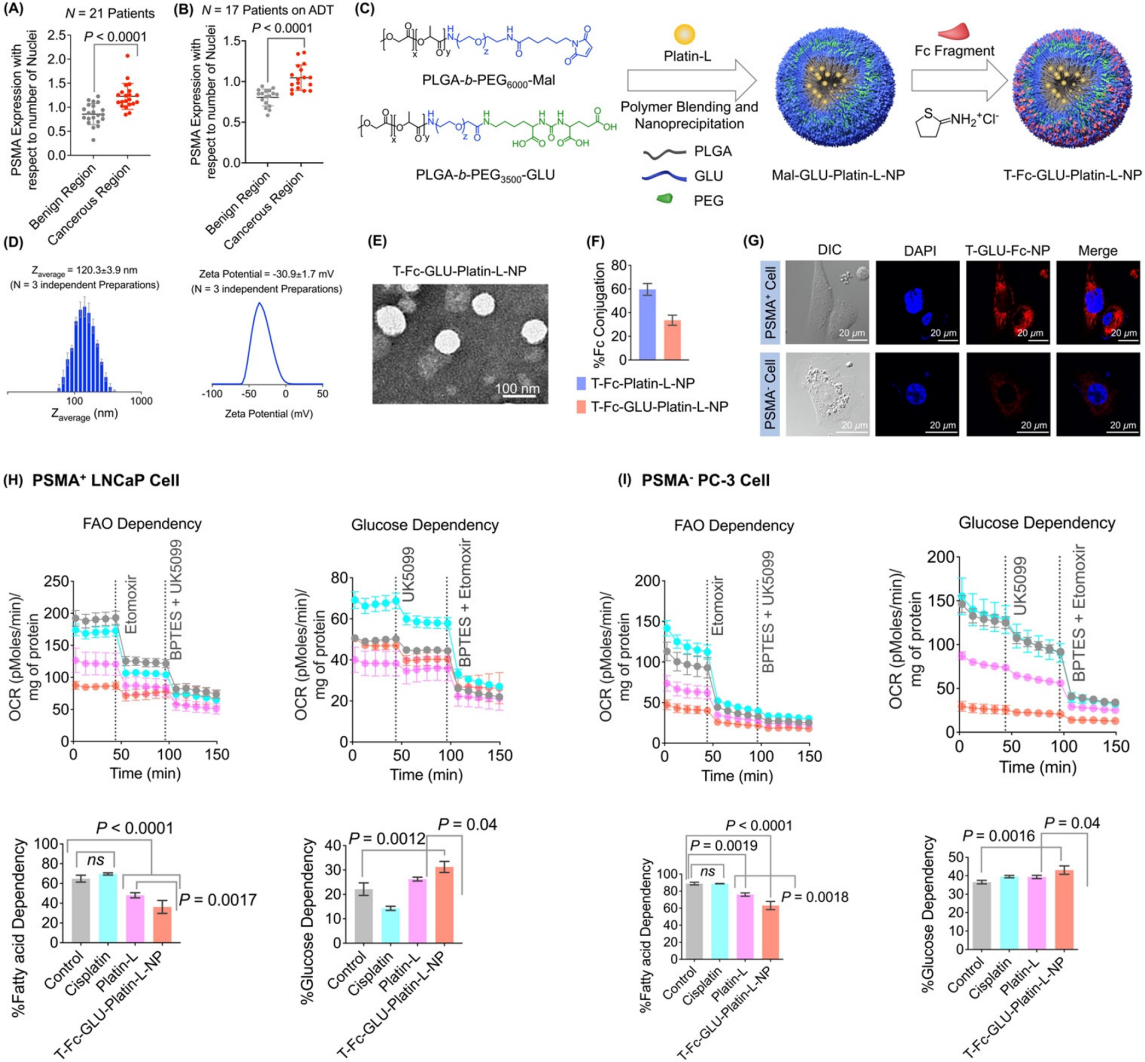
Targeted nanoparticle of Platin-L for clinically relevant formulation development. Scatter plot indicating the relative expression of PSMA in (A) non-ADT-treated patient samples and (B) ADT-treated patient samples between benign and cancerous regions with respect to the nuclei. Quantification was done on the images using ImageJ software for an area containing around 30 nuclei. Statistical analyses were carried out using a two-tailed paired *t* test with an α value of 0.05. (C) Synthesis of Platin-L-loaded nanoparticles made from PLGA-*b*-PEG_3500_-GLU and PLGA-*b*-PEG_6000_-Mal, followed by conjugation of the Fc immunoglobulin fragment producing dual-targeted T-Fc-GLU-Platin-L-NPs. (D) Hydrodynamic diameter and zeta potential of dual-targeted NPs using DLS. (E) Analyses of morphology and diameter of dual-targeted NPs using TEM. (F) Percent Fc conjugation of Platin-L-loaded nanoparticles made with PLGA-*b*-PEG_6000_-Mal and dual-targeted nanoparticles. (G) PSMA-mediated uptake of the targeted nanoparticles in PSMA-positive LNCaP and PSMA-negative PC3 cells. (H) Fatty acid dependency and glucose dependency of cisplatin, Platin-L, and T-Fc-GLU-Platin-L-NP in LNCaP cells. [Platin-L]: 50 μM and [cisplatin]: 50 μM. The concentration of T-Fc-GLU-Platin-L was calculated with respect to [Pt]: 50 μM. Statistical analysis was carried out using an ordinary one-way ANOVA analysis with an α value of 0.05. (I) Fatty acid dependency and glucose dependency of cisplatin, Platin-L, and T-Fc-GLU-Platin-L-NP in PC3 cells. Statistical analysis was carried out using an ordinary one-way ANOVA analysis with an α value of 0.05.

Liver plays an important role in regulating the systemic metabolism of fatty acids, and hepatic fatty acid β oxidation represents an important component.^59^ We investigated the substrate dependence of HepG2 hepatocytes (Supporting Information Figure S23) and the FAO inhibitory properties of Platin-L in these cells (Supporting Information Figure S23). These studies indicated that Platin-L diminishes the fatty acid-based metabolism in these cells; however, the extent of FAO inhibition was less in the hepatocytes than in those seen in PCa cell lines as the drop-in OCR value was less compared to that seen with etomoxir (Supporting Information Figure S23). Most low-molecular-weight drug candidates show poor pharmacokinetic (PK) parameters and unfavorable biodistribution (bioD) properties when evaluated *in vivo* or in clinical trials. Our preliminary bioD studies with Platin-L indicated that this compound is mostly distributed in the liver and kidneys 24 h after systemic administration in normal BALB/c albino mice (Supporting Information Figure S24). Thus, we hypothesized that since Platin-L shows a significant distribution in the liver and affects the metabolic profiles of the hepatocytes, for clinical translation, this compound needs to be formulated for targeted delivery to prostate tumors. Polymeric NPs of poly(lactide-*co*-glycolide)-*b*-polyethylene glycol (PLGA-*b*-PEG) block copolymers are especially promising as drug delivery vehicles.^60−63^ We worked on several aspects of the development of aptamer-targeted PLGA-*b*-PEG NPs that differentially bind to and get taken up by PCa cells^31,63,64^ and also small-ligand-based prostate specific membrane antigen (PSMA) targeted NPs.^42^ We stress that cancer of the prostate is generally less vascularized, resulting in a poor enhanced permeability and retention (EPR) effect,^65^ and thus we hypothesized that we need to use a targeted NP system using an active targeting approach for FAO inhibition using Platin-L under clinical settings of PCa. We first investigated whether the same pool of patient samples we previously analyzed for CPT1A also have PSMA expression. Thus, we analyzed the same pool of ADT- and non-ADT-treated patients by immunofluorescence to check the expression of PSMA. The data revealed that the expression of PSMA was significantly higher in the cancerous region of both non-ADT- and ADT-treated groups as compared to the respective benign regions (Figure 4A, 4B, Supporting Information Figures S25, S26). In order to target the PSMA expressing prostate cancer cells, we developed biodegradable orally administrable PSMA targeted nanoparticles from PLGA-*b*-PEG_3500_-GLU and PLGA-*b*-PEG_6000_-Mal polymers which were previously reported by us^66^ (Figure 4C). Using these polymers, Platin-L-loaded dual-targeted NPs (T-Fc-GLU-Platin-L-NPs) were synthesized via a nanoprecipitation technique using a ratio of 50:50 PLGA-*b*-PEG_3500_-GLU/PLGA-*b*-PEG_6000_-Mal and a 20% feed of Platin-L with respect to the total amount of polymer (Figure 4C). NPs were also synthesized with the individual polymers and characterized in comparison to the blended NP (Supporting Information Figure S27). Furthermore, these nanoparticles were conjugated with the Fc antibody fragment on PLGA-*b*-PEG_6000_-Mal by ene–thiol chemistry (Figure 4C). The hydrodynamic diameter and zeta potential of the nanoparticles were measured using dynamic light scattering (DLS) and were found to be ∼120 nm and ∼−30 mV, respectively (Figure 4D). The morphology of Platin-L-loaded dual-targeted nanoparticles revealed to be spherical using transmission electron microscopy (TEM) (Figure 4E). The Fc fragment conjugation efficiency was determined by the bicinchoninic acid assay. A high conjugation efficiency of ∼60% was observed for T-Fc-Platin-L-NPs, and a value of ∼30% was observed for T-Fc-GLU-Platin-L-NPs (Figure 4F). We also studied the release kinetics of Platin-L from T-Fc-GLU-Platin-L-NPs under a physiological pH of 7.4 and at 37 °C. This study indicated a controlled release profile of Platin-L from the NPs (Supporting Information Figure S27). Our studies using an optical probe loaded uptake of T-Fc-GLU-Cy5.5-NPs indicated higher amounts of targeted NPs in PSMA expressing LNCaP cells than in PSMA negative PC3 cells, suggesting that our nanoparticle platform has an effective targeting ability for PSMA expressing prostate cancer cells (Figure 4G). Fuel flex analyses were performed using the seahorse analyzer to determine the metabolic dependencies of the PSMA^+^ and PSMA^–^ cells. The % fatty acid dependency in PSMA^+^ cells showed significant reduction upon treatment with Platin-L alone and T-Fc-GLU-Platin-L-NPs as compared to cisplatin and the control (Figure 4H, Supporting Information Figure S28). However, the nanoparticle-treated group showed the maximum reduction. Furthermore, we observed a significant increase in the % glucose dependency upon treatment with the T-Fc-GLU-Platin-L-NPs compared to the other treatment articles (Figure 4H). In the case of PSMA^–^ cells, a similar trend of decreased % fatty acid dependency with increasing % glucose dependency was observed upon treatment with T-Fc-GLU-Platin-L-NP (Figure 4I).

### Efficacy of T-Fc-GLU-Platin-L-NP in LNCaP Xenograft Mouse Model

**Figure 5:**
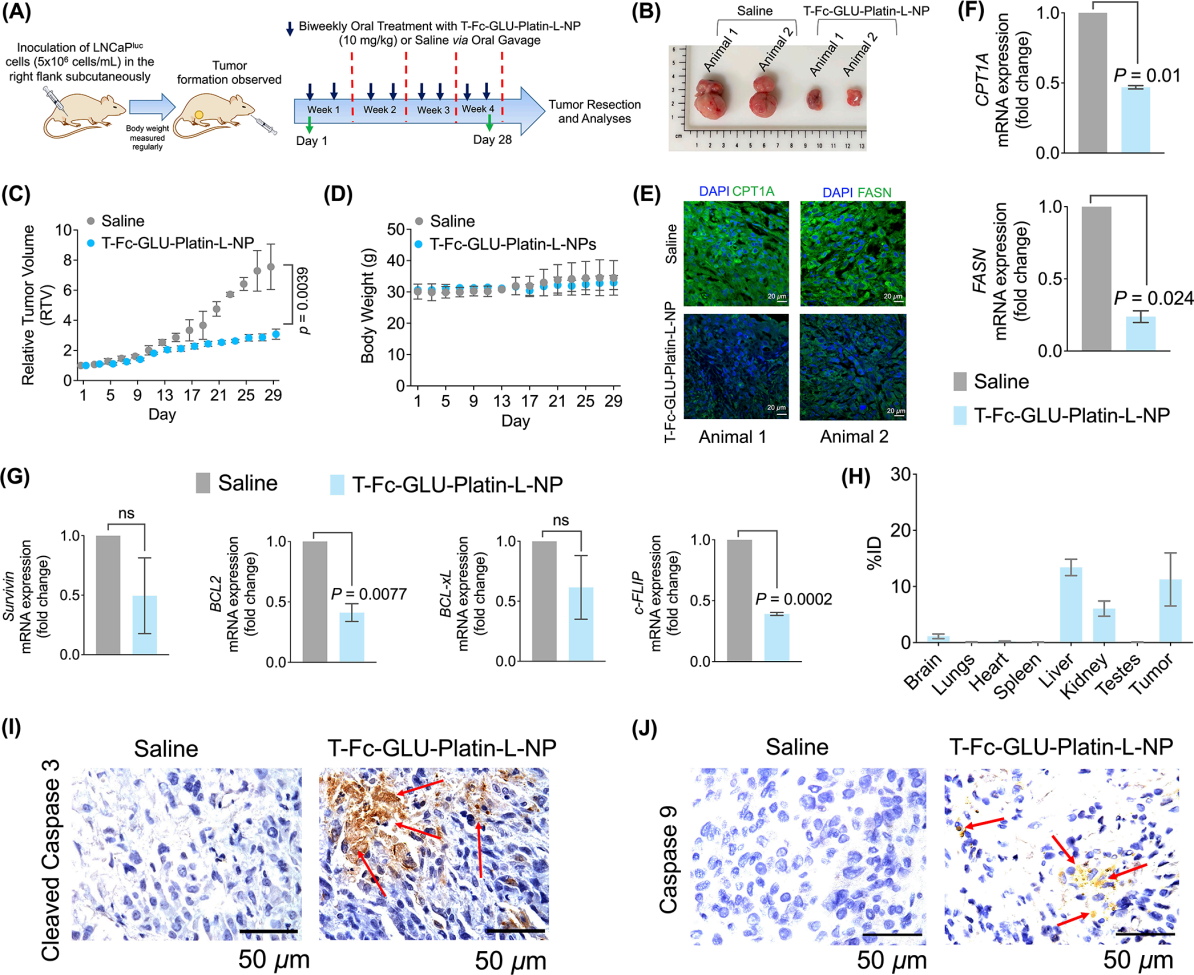
Efficacy of T-Fc-GLU-Platin-L-NP in the LNCaP xenograft mouse model. (A) Schematic representation of the treatment regimen of the LNCaP xenograft mouse model. (B) Images showing the tumor mass formation in the treatment group as compared to its saline control. (C) Scatter plot showing the relative tumor volume (RTV) between the saline and the T-Fc-GLU-Platin-L-NP treated group and the (D) change in body weight of the animals during the treatment regimen. (E) Immunofluorescence (IF) studies performed on the tumor tissues showing the reduction of CPT1A and FASN in the T-Fc-GLU-Platin-L-NP-treated group of animals as compared to the saline group. RT-PCR studies showing the fold change in the mRNA expression of (F) fatty acid oxidation-related (FAO) genes, *CPT1A* and *FASN*. (G) Antiapoptotic genes *Survivin*, *BCL2*, *BCL-xL*, and *cFLIP*. Statistical analysis was carried out using an unpaired *t* test with an α value of 0.05. (H) Biodistribution of T-Fc-GLU-Platin-L-NPs in all the organs of the animals. Immunohistochemical (IHC) analysis of apoptotic markers (I) cleaved caspase 3 and (J) caspase 9 in tumor tissues. Red arrows indicate the staining of cleaved caspase 3 and caspase 9.

To analyze the potency of our nanoparticles, we established an LNCaP xenograft mouse model. The group receiving the nanoparticle treatment was administered with T-Fc-GLU-Platin-L-NPs orally at a dosage of 10 mg/kg twice weekly for 4 weeks. We did not consider utilizing NPs with encapsulated cisplatin or Platin-4 for two reasons. First, the physicochemical properties of cisplatin would prevent its effective loading inside the hydrophobic core of the NP, and second, we are aware that both cisplatin and Platin-4 would demonstrate an anticancer effect, as they are potent chemotherapeutic agents. However, our goal is to demonstrate the ability of Platin-L to inhibit cancer growth through CPT1A and mitochondrial pathways. At the end of the treatment regimen, the mice were euthanized, and all the organs and tumors were harvested for *ex vivo* analyses (Figure 5A). The data from the representative images of the saline- and nanoparticle-treated tumor samples revealed lowering of the tumor mass in the nanoparticle-treated group (Figure 5B). The relative tumor volume (RTV) showed that there was a significant reduction in tumor volume in the T-Fc-GLU- Platin-L-NP-treated group as compared to the saline group (Figure 5C) without any significant change in body weight of the animals (Figure 5D). Immunofluorescence studies from the tumor tissues showed downregulation of FAO-related proteins CPT1A and FASN, suggesting that the nanoparticles can alter the tumor cell metabolism (Figure 5E). These observations were further confirmed by RT-PCR studies where the mRNA expression of both *CPT1A* and *FASN* genes in the tumor tissue was downregulated by the treatment of T-Fc-GLU-Platin-L-NP (Figure 5F). It is possible that upon inhibition of CPT1A, the cell recognizes that synthesizing more long-chain fatty acids for β oxidation is futile, resulting in the downregulation of *FASN.* Furthermore, the expressions of the antiapoptotic genes *Survivin*, *BCL2*, *BCL-xL*, and *cFLIP* were downregulated upon nanoparticle treatment, suggesting that the treatment was able to induce cell death in these tumor tissues by inducing apoptosis (Figure 5G). This could be the result of the Randle effect where once FAO is inhibited in the cells, they switch to glucose utilization, which results in the increase in apoptosis. The organs that were harvested from the animals were utilized for the biodistribution studies. We observed significant presence of the nanoparticles in the tumor (Figure 5H). The ability of the nanoplatform to induce apoptosis in tumor tissues was further confirmed by immunohistochemical (IHC) studies. The data showed higher expressions of apoptotic proteins, cleaved caspase 3 (Figure 5I and Figure S29), and caspase 9 (Figure 5J and Figure S29) in the nanoparticle-treated group as compared to the saline group. Multiple images from different animals were used to score these markers (Figure S29) to conclude a higher level of apoptosis in NP-treated tumors. The extent of protein expression was scored on a scale of 0–5: 0 (no detectable stain), 1 (few nuclei were stained), 2 (up to 10% of the nuclei were stained), 3 (10–50% of the nuclei were stained), and 4 (>50% of the nuclei were stained).^67^

### Efficacy of T-Fc-GLU-Platin-L-NPs in Cisplatin-Resistant Mouse Model

**Figure 6:**
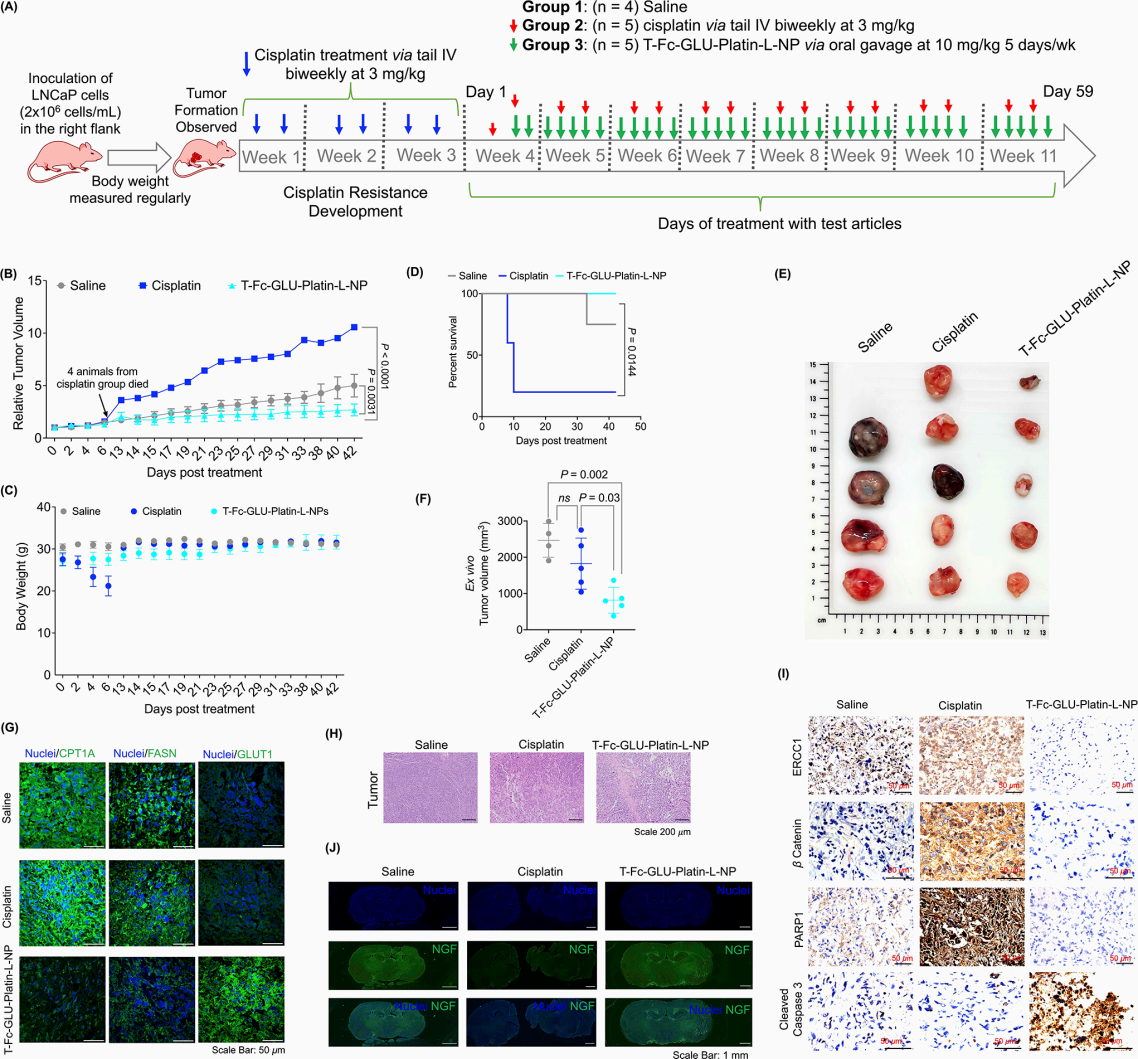
Efficacy of T-Fc-GLU-Platin-L-NPs in the cisplatin-resistant LNCaP xenograft mouse model. (A) Schematic representation of the development of the cisplatin-resistant LNCaP mouse xenograft model showing the timeline of LNCaP inoculation followed by the regular biweekly dosage of cisplatin to acquire cisplatin resistance and finally treatment with T-Fc-GLU-Platin-L-NPs to analyze its efficacy. (B) Relative tumor volume during the period of treatment with NP. The statistical significance was analyzed using two-way ANOVA analysis with an α value of 0.05. (C) Body weight of the mice from the different groups throughout the study. (D) Kaplan–Meier survival curve of the study. Statistical analysis was done with a log-rank (Mantel–Cox) test with a bonferroni correction for multiple comparisons applied; the α value was 0.05. (E) Images of excised tumors from each mouse from the study. (F) *Ex vivo* tumor volumes of the different treatment groups. Statistical analysis was done with an ordinary one-way ANOVA analysis with an α value of 0.05. *Ex vivo* volume analysis provides an accurate measurement of the tumors, which accounts for the difference in volume from (B) to (F). (G) IF studies on the tumor tissues showing the reduction of CPT1A, FASN, and GLUT1 in the T-Fc-GLU-Platin-L-NP-treated group compared to saline and cisplatin groups. (H) Hematoxylin and eosin (H & E) staining of tumor sections. (I) Immunohistochemical (IHC) analysis from the tumor tissue samples showing the change in expression of different cisplatin resistance genes, namely, ERCC1, β-catenin, and PARP1, and apoptotic-marker-cleaved caspase 3 upon treatment with T-Fc-GLU-Platin-L-NP as compared to its saline- and cisplatin-treated animals. (J) Immunofluorescence staining of whole brain sections for nerve growth factor (NGF). Scale bar = 1 mm.

To study the efficacy of the T-Fc-GLU-Platin-L-NPs in a cisplatin-resistant PCa model, we established an LNCaP xenograft mouse model. After tumor formation, the mice were injected with cisplatin at a dosage of 3 mg/kg twice a week for 2 weeks to develop resistance as evidenced by no significant reduction in tumor growth (Figure 6A for experimental details, Supporting Information Figure S30 for tumor growth, group assignment, and body weight during the resistance development period). After cisplatin treatment, the mice were randomly divided into three groups and treated with saline (*n* = 4, this group did not receive any cisplatin prior), cisplatin at 3 mg/kg via iv (*n* = 5), or T-Fc-GLU-Platin-L-NP at a dosage of 10 mg/kg with respect to Platin-L with an oral gavage (*n* = 5). The treatment regimen was followed for 8 weeks (Figure 6A). Complete characterization of all batches of T-Fc-GLU-Platin-L-NPs used during the dosing period is presented in Supporting Information Figure S31. Treatment with NP via oral gavage resulted in a remarkable tumor reduction compared to saline and cisplatin groups (Figure 6B). The cisplatin treatment group showed a significant loss in body weight (Figure 6C) and poor survival due to developed resistance (Figure 6D); however, the animals from the NP-treated group continued to have steady body weight and significantly increased survival (Figure 6B and 6C). As several mice from cisplatin treated group died due to tumor burden, the tumors and other tissue were stored for future analysis. Mice in the saline group showed uncontrollable tumor growth. In particular, one mouse from the saline group developed a tumor later in the study and still became larger than mice in the NP-treated group (Figure 6B and Supporting Information S32). Due to the uncontrollable tumor growth in the saline group, the treatment with the NP could not be continued and the study was terminated. However, the treatment with NP under cisplatin resistance demonstrated a significant reduction and control of the tumors (Figure 6B). At termination, *ex vivo* tumor volumes were calculated from all tumors including tumors from the mice which died from the cisplatin treated group during the experiment; we documented that NP-treated group had significantly less tumor volume compared to saline- and cisplatin-treated groups (Figure 6E and Figure 6F, Supporting Information Figure S32). Immunofluorescence studies from the tumor tissues confirmed NP-mediated inhibition of FAO-related proteins CPT1A and FASN and upregulation of glucose transporter 1 (GLUT1) implying upregulation of glucose oxidation in the cisplatin-resistant model (Figure 6G). This upregulation could be the result of the Randle effect and the cells’ response to the shutdown of the FAO pathway. Tumor tissue were analyzed via H&E staining, revealing much destruction of tumor cells compared to that in saline- or cisplatin-treated groups (Figure 6H). Immunohistochemistry was conducted to evaluate cisplatin resistance markers in the tumor tissue. The data revealed that DNA excision repair protein ERCC1, which plays a significant role in promoting nucleotide excision repair, was found to be expressed in the saline-treated group, which increased upon cisplatin treatment due to the increased cisplatin resistance. However, we observed a significant decrease in ERCC1 expression in the T-Fc-GLU-Platin-L-NP-treated group, suggesting that our NPs have the potential to sensitize the otherwise resistant cells (Figure 6I and Figure S33). Similarly, β-catenin, which is responsible for bypassing the apoptotic pathway in cisplatin-resistant cells, was observed to be decreased in T-Fc-GLU-Platin-L-NP-treated tumor samples as compared to cisplatin- and saline-treated samples (Figure 6I and Figure S33). The data also showed that there is an overexpression of PARP in cisplatin-resistant tumor samples. PARP, an important DNA repair protein which is also involved in metabolic pathways of cisplatin-resistant tumors, decreased upon treatment with T-Fc-GLU-Platin-L-NP (Figure 6I and Figure S33). Furthermore, cleaved caspase 3, a crucial apoptotic marker, was observed to be increased in the nanoparticle-treated group as compared to saline- and cisplatin-treated sample (Figure 6I and Figure S33). Taken together, these data along with quantification presented in Figure S33 using multiple images from all animals across the study demonstrated that Platin-L utilizes CPT1A binding followed by mitochondrial dislocation through the protein to act on the mitochondrial DNA, producing repair-resistant DNA adducts to promote apoptosis.

Peripheral neuropathy induced by cisplatin treatment is a well-established side effect. To evaluate the extent of peripheral neuropathy in these treated animals, we analyzed the mouse brains from each group for nerve growth factor (NGF) which is essential in allowing pain signals from the peripheral to reach to the central nervous system.^68^ The mice in the cisplatin group displayed reduced NGF expression compared to the mice from the saline- and NP-treated groups, indicating that there may be less peripheral neuropathy caused by the NP treatment (Figure 6J). H&E stained images of all other tissues were analyzed, and no significant toxic changes were observed (Supporting Information Figure S32).

## Conclusions

The impact of this current targeted metabolic modulation of advanced prostate cancer extends beyond this cancer type. The reported mechanistic investigations will allow us to find the clues to make this platform more general to be used for cancers where the cellular pathways can be altered. To our knowledge, this is the first cisplatin prodrug which can modulate the mitochondrial metabolism and respiration of prostate cancer cells by inhibiting fatty acid metabolism forcing these populations to undergo apoptosis and possibly address the resistance of prostate cancer to platinum-based therapy and offer the use of cisplatin for this deadly disease. We discovered that a cisplatin prodrug, Platin-L, can inhibit the FAO of PCa cells by interacting with CPT1A and thereby disrupting the transfer of long-chain fatty acids to the mitochondria for metabolism. Our further studies synthesizing additional prodrugs documented that the presence of succinate near lauric acid is crucial for CPT1A binding and supported our hypothesis that Pt-bound succinate can be activated by acyl-CoA-synthase to form acyl-CoA, which then helps Platin-L to bind CPT1A efficiently. Additionally, in a cisplatin-resistant tumor mouse model, we demonstrated that Platin-L-loaded orally administrable nanoparticles are effective at reducing the tumor volume and peripheral neuropathy caused by cisplatin treatment. This distinctive observation of cisplatin prodrug activity is of clinical relevance as it offers the potential use of cisplatin for this otherwise resistant disease.

### Patient Samples

All patient samples were obtained by Dr. Kryvenko under approved University of Miami IRB protocols. All patients enrolled are in a phase II randomized clinical trial called the “MRI-Guided Prostate Boosts Via Initial Lattice Stereotactic versus Daily Moderately Hypofractionated Radiotherapy (BLaStM)” trial (NCT02307058). These tissues are deidentified by the University of Miami BSSR, where an arbitrary identifier was assigned so that all recipients (including the clinicians) were blinded to information that identifies or could be used to identify the donor subject from whom the material was obtained.

### Animals

BALB/c nude and BALB/c albino male mice (4 to 5 weeks old) were purchased from Jackson Laboratory. All animals were handled in accordance with “The Guide for the Care and Use of Laboratory Animals” of the American Association for Accreditation of Laboratory Animal Care (AAALAC), the Animal Welfare Act (AWA), and other applicable federal and state guidelines. All animal work presented here was approved by the Institutional Animal Care and Use Committee (IACUC) of the University of Miami (UM) Miller School of Medicine (animal protocol number 20-171, IBC protocol number IBC 20-140). For the cisplatin-resistant model, there was approval to use a 3000 mm^3^ tumor volume as the termination point due to the nature of the experiment for resistance development followed by treatment (addendum number 20-171-ad07). All housing, surgical procedures, and experimental protocols were approved by the IACUC Committee of UM. Animals had free access to food and water during all experiments.

### Materials and Methods

Descriptions of materials and methods, cell lines, chemicals, biochemicals, synthesis of targeting ligands, polymers, NPs, and other experimental and characterization methods are described in the Supporting Information.

### Statistics

All data were expressed as the mean ± SD (standard deviation). Statistical analyses were performed using GraphPad Prism software v. 5.00. Comparisons between two values were performed using an unpaired Student *t* test. One-way ANOVA with a posthoc Tukey test was used to identify significant differences among the groups.

## Supporting Information Available

Description of materials and methods, cell lines, chemicals, biochemical, synthesis of targeting ligands, polymers, NPs, other experimental and characterization methods, and additional data (PDF)
