# Correction to “New Pathway for Cisplatin Prodrug to Utilize Metabolic Substrate Preference to Overcome Cancer Intrinsic Resistance”

**DOI:** 10.1021/acscentsci.3c00920

**Published:** 2023-08-08

**Authors:** Akil A. Kalathil, Subham Guin, Akash Ashokan, Uttara Basu, Bapurao Surnar, Katiana S. Delma, Leonor M. Lima, Oleksandr N. Kryvenko, Shanta Dhar

After the publication, we realized that the clinical trial name mentioned in the “Patient Samples” section is not correct. The “Patient Samples” section should read as below:

## Patient Samples

From the prospective radical prostatectomy database maintained by Dr. Kryvenko, we obtained patient samples from treatment naïve and salvage radical prostatectomies with preoperative neoadjuvant androgen deprivation therapy or chemotherapy. All tissue samples submitted to the research lab were deidentified. An arbitrary identifier was assigned to each specimen so that all recipients were blinded to information that identifies or could be used to identify the donor subject from whom the material was obtained.

