# Supplementary material for: New Pathway for Cisplatin Prodrug to Utilize Metabolic Substrate Preference to Overcome Cancer Intrinsic Resistance
Source: ACS Cent Sci. 2023 Jul 12;9(7):1297–312. doi: 10.1021/acscentsci.3c00286 (PMC10375877; doi:10.1021/acscentsci.3c00286)
Supplement: Supplementary file 1 — oc3c00286_si_001.pdf [file oc3c00286_si_001.pdf]

Supplementary Information

New Pathway for Cisplatin Prodrug to Utilize Metabolic Substrate Preference to Overcome Cancer Intrinsic Resistance

Akil A. Kalathil, [a§] *Subham Guin,*[a§] *Akash Ashokan,*[a,b§] *Uttara Basu,*[a,b§] *Bapurao Surnar,*[a,b] *Katiana S. Delma,*[c] *Leonor M. Lima,*[c] *Oleksandr N. Kryvenko,*[c,b,e,f] *and Shanta Dhar*[*a,b,d]

[a]NanoTherapeutics Research Laboratory, Department of Biochemistry and Molecular Biology, University of Miami Miller School of Medicine, Miami, FL 33136
[b]Sylvester Comprehensive Cancer Center, University of Miami Miller School of Medicine, Miami, FL 33136
[c]Department of Pathology and Laboratory Medicine, Miller School of Medicine, University of Miami, Miami, FL 33136
[d]Department of Chemistry, University of Miami, Coral Gables, FL 33146
[e]Department of Radiation Oncology, Miller School of Medicine, University of Miami, Miami, FL 33136
[f]Desai Sethi Urology Institute, Miller School of Medicine, University of Miami, Miami, FL 33136
[§]Equally contributed authors
*To whom correspondence should be addressed. Email: shantadhar@med.miami.edu

Chemicals: All chemicals were used as received without further purification unless otherwise noted. Etomoxir, 2-Cyano-3-(1-phenyl-1H-indol-3-yl)-2-propenoic acid (UK-5099), Bis-2-(5-phenylacetamido-1,3,4-thiadiazol-2-yl)ethyl sulfide (BPTES), Krebs-Henseleit Buffer (KHB), Roswell Park Memorial Institute Medium (RPMI), oligomycin, carbonyl cyanide 4-(trifluoromethoxy)phenylhydrazone (FCCP), rotenone, antimycin-A, cerulenin, cisplatin(15663-27-1), polyethylene glycol diamine(ClH_3N-PEG_{6000}-NH_3Cl), Dodecanoic acid(143-07-7), L-carnitine(541-15-1), triphenylphosphine(603-35-0), Palmitoyl coenzyme A lithium salt(188174-64-3), Acetyl coenzyme A lithium salt (32140-51-5), Malonyl coenzyme A lithium salt(108347-84-8), dimethyl sulfoxide (DMSO) (67-68-5), hydrogen peroxide (7722-84-1), dimethyl formamide (DMF) (68-12-2) were purchased from Sigma Aldrich. Docetaxel was purchased from LC laboratories. L-Glutamine (2530081), penicillin/streptomycin (10378016), trypsin-ethylenediaminetetraacetic acid (EDTA) solution (25200056) was purchased from ThermoFisher Scientific. Polyethylene glycol (HOOC-PEG_{3500}-NH_3Cl) was purchased from Jenkem, (2-[4-(2-hydroxyethyl) piperazin-1-yl]ethanesulfonic acid (HEPES) buffer (1 M) (15630130) and sodium pyruvate (11360070) was purchased from ThermoFisher Scientific. Roswell Park Memorial Institute (RPMI) medium, Dulbecco's Modified Eagle Medium (DMEM), fetal bovine serum (FBS), phosphate buffered saline (1X PBS) was purchased from Gibco (reference number 10010-023). Regenerative cellulose membrane Amicon Ultra centrifugal 100 kDa filters were purchased from Merck Millipore Ltd. PLGA-COOH of inherent viscosity dL/g, 0.15 to 0.25 was purchased from Durect LACTEL® Absorbable Polymers. XFe96 FluxPaks (SKU 102416-100) and Seahorse XF Palmitate-BSA FAO Substrate were purchased from Agilent Seahorse Bioscience. PC3, LNCaP, DU145, 22RV1, and HepG2 cells were purchased from American Type Culture Collection (ATCC). All RT-PCR primers were purchased from Integrated DNA Technologies (IDT). DAB chromogen SignalStain® DAB Substrate Kit #8059 was purchased from Cell Signaling Technology. Cleaved caspase 3 (catalog #9661) and hematoxylin (catalog #14166) were purchased from Cell Signaling Technology. Cleaved caspase 9 (ab25758), CPT1A (ab220789), FASN (ab128870), *β*-Actin (ab8226), ERCC1 (ab129267), Beta Catenin (D10A8), NGF antibody [EP1320Y] (ab52918), Hsp60 (ab190828), Histone H3 (ab1791), Calnexin (ab133615) and SIRT2 antibody (ab211033) were purchased from abcam. PARP (Antibody#9542) and TFAM (D5C8) was purchased from Cell Signalling Technology. BDNF (Catalog # PA5-15198) was purchased from Invitrogen. NF-L Antibody (NFL/736) (NBP2-44893) – was purchased from Novus Biologicals. Mitochondria isolation kit (Catalog number: 89874) was purchased from ThermoFisher Scientific. The western blots were developed using Bio-Rad clarity western ECL substrate (1705061) was purchased from Bio-Rad. RIPA Lysis and Extraction Buffer (catalog number: 89900) was purchased from ThermoFisher Scientific. Protease Inhibitor Cocktail (200-664-3) was purchased from Sigma Aldrich. TRIzol™ (15596026) was purchased from ThermoFisher Scientific. RNeasy Micro Kit (74004) was purchased from Qiagen. SYBR® Green Master Mix (15596026) and iScript™ (1708890) were purchased from Bio-Rad. DMEM was purchased from Gibco (Gibco™ 11995073), RPMI 1640 was purchased from Gibco (Gibco™ 21870076). DAPI Solution was purchased from ThermoFisher Scientific Catalog number: 62248. Platinum standard (ICP-078) was purchased from Agilent Technologies. Hydrochloric acid (320331-500ML) and nitric acid (438073-500ML). The Firefly

Luciferase LentifectTM purified lentiviral particles (catalog number: LPP-HLUC-LV105-025-C) were purchased from GeneCopoeia.

Instruments
Distilled water was purified by passage through a Millipore Milli-Q Biocel water purification system (18.2 MΩ) containing a 0.22 *μ*m filter. Dynamic light scattering (DLS) measurements were carried out using a Malvern Zetasizer Nano ZS system. Cells were counted using Countess® Automated Cell Counter procured from Invitrogen life technology. Inductively coupled plasma mass spectrometry (ICP-MS) studies were performed on an Agilent 7900 ICP-MS instrument. Mitochondrial bioenergetics assays were performed on $XF^{e}96$ Extracellular Flux Analyzers (Agilent Seahorse Biosciences). Immunohistochemistry was performed under Leica microscope. Confocal microscopy was performed under Olympus FluoView FV3000 confocal microscope. TEM images were recorded using JEOL JEM-1400 instrument. Western blot images were taken by Bio-Rad ChemiDoc MP. RT-PCR experiments were performed in Bio-Rad CFX Real-Time PCR Detection Systems. ^{1}H, ^{13}C and ^{195}Pt NMR were recorded using Bruker 400 MHz instrument. VS-120 microscope was used to image whole mouse brain sections.

Synthesis of Platinum prodrugs: Synthesis of Platin-L or Platin 12,[8] Platin-4,[8] Platin-M,[9] Platin-Az,[9, 10] Platin-A,[6] Platin-B[11] were carried out based on our lab reported procedures.

Synthesis of Platin-L-4
Cisplatin (2.0 g, 6.67 mmol) was stirred in 100 mL of H_2O_2 in a round bottom flask (RBF) at 65 °C for 4 h. After 4 h, the reaction mixture was cooled down to room temperature and filtered to yield *cis,cis,trans*(diaminedichlorodihydroxy)platinum(IV) (**1**). Yield = 1.64 g (74.4%).

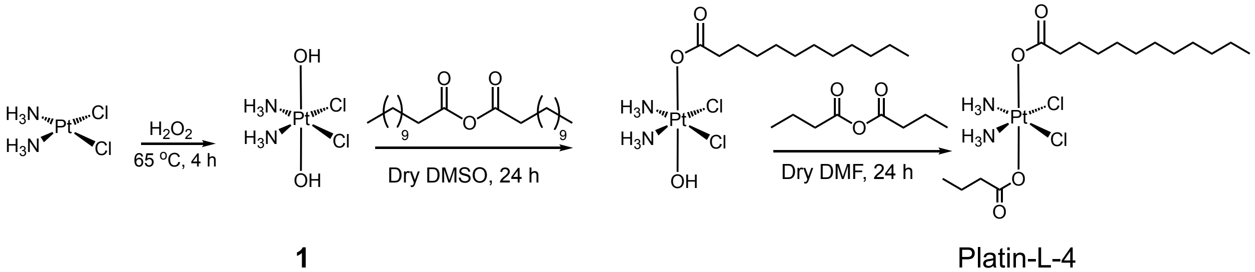

Compound **1** (0.5 g, 1.5 mmol) was dissolved in 10 mL of DMSO in an RBF with stirring. Lauric anhydride (0.57 g, 1.5 mmol) was dissolved in 5 mL of DMSO and added dropwise into the reaction. The reaction mixture was stirred at room temperature for 24 h. This reaction mixture was extracted using diethyl ether to reduce the amount of DMSO, and finally the product was precipitated in acetone to yield a pale-yellow colored compound. This pale-yellow compound (0.2 g) was dissolved in 15 mL of dimethylformamide (DMF) and butyric anhydride (0.193 g, 0.8 mmol) was added to the reaction mixture with stirring. The reaction was stirred overnight at room temperature. DMF was evaporated under reduced pressure. Impurities were removed by precipitating in diethyl ether and

centrifuging at 6000 rpm for 5 min at 4 ℃. The diethyl ether layer was collected and dried under reduced pressure to yield Platin-L-4. Yield= 0.16 g (64%). ^{1}H NMR ($CDCl_3$, 400 MHz): 6.6-6.45 (6H, m); 2.21-1.21 (4 H, t);1.49-1.43 (4 H, m); 1.29 (12 H, m); 0.88-0.84 (6 H, m) ppm. ^{13}C ($CDCl_3$, 400 MHz): 181.27, 38.47, 31.77, 29.50, 29.44, 29.18, 25.92, 22.56, 19.32, 14.42 ppm. ^{195}Pt ($CDCl_3$, 400 MHz): 1232.809 ppm.

Lipophilicity of Platin-L-4

In order to determine the lipophilicity of Platin-L-4, 1-octanol and phosphate buffered saline (PBS) were used as organic and aqueous systems. To make the Platin-L-4 solution in PBS saturated octanol, equal volumes of 1-octanol (5 mL) and PBS (5 mL) were mixed and stirred vigorously for 16 h. The layers were separated carefully using separating funnel and the organic layer was collected as PBS saturated 1-octanol. Platin-L-4 solution was made using the PBS saturated 1-octanol at a concentration of 500 μM in 5 mL. To this, 5 mL of PBS was added and stirred vigorously for 16 h. Both, organic and aqueous layers were separated carefully using separating funnel and Platin-L-4 concentration was measured in both organic and aqueous layers using ICP-MS. Log P value was determined by using the following equation: $\log P=\log([\text{Platin-L-4}_{Oct}]/ [\text{Platin-L-4}_{PBS}])$, where Platin-L-$4_{Oct}$ indicates Platin-L-4 concentration in octanol and Platin-L-4_{PBS} indicates the Platin-L-4 concentration in the PBS.

Cell Lines and Cell Culture

Human prostate cancer cell lines 22RV1, DU145, PC3, and LNCaP were procured from the American Type Culture Collection (ATCC). The cells were grown at 37 °C in 5% CO_2 in RPMI 1640 medium supplemented with 10% FBS, 1% sodium pyruvate, 1% HEPES, and 1% penicillin/streptomycin. Hepatocytes, HePG2 cells were grown at 37 °C in 5% CO_2 in Dulbecco's Modified Eagle's Medium (DMEM) supplemented with 1% L-glutamine, 1% sodium pyruvate, 1% penicillin/streptomycin, and 10% FBS. Cells were passed every 3 to 4 days and restarted from frozen stocks after 20 passages.

Animals

Male nude BALB/c mice about 3-4 weeks old and BALB/c Albino male mice were purchased from Jackson Laboratory. All mice were maintained in accordance with ''The Guide for the Care and Use of Laboratory Animals'' of American Association for Accreditation of Laboratory Animal Care (AAALAC), Animal Welfare Act (AWA), and other applicable federal and state guidelines. All animal data showed here has been approved by Institutional Animal Care and Use Committee (IACUC) of University of Miami (UM) Miller School of Medicine (Animal Protocol Number: 20-171, IBC Protocol Number: IBC 20-140). All housing, surgical procedures, and experimental protocols were approved by the IACUC Committee of UM. Animals had free access to diet and water during all experiments.

MitoStress Assay

PC3 and LNCaP cells were seeded at a density of 20,000 cells per well in Seahorse XFe96 well plates in RPMI-1640 supplemented with 10% FBS, 1% sodium pyruvate and 1% HEPES buffer at 37 °C in 5% CO_2. After 24 h, the media was replaced with fresh media and Platin-L (10 μM) was added. After 24 h of incubation, the media was removed

and the cells were washed thrice using bicarbonate and serum free DMEM, supplemented with glucose, 1.8 mg/ mL; 1% glutamine and 1% sodium pyruvate and incubated in a non-CO_2 incubator at 37 °C for 1 h. Mitostress assay was run using oligomycin (1 μM), FCCP (1 μM), and a mixture of antimycin-A/ rotenone (1 μM) each in ports A, B, and C respectively using Seahorse XFe96 Extracellular Flux Analyzer.

Glycolytic Stress Assay

PC3 and LNCaP cells were seeded at a density of 20,000 cells per well in Seahorse XFe96 well plates in RPMI-1640 supplemented with 10% FBS, 1% sodium pyruvate, 1 % penicillin/streptomycin and 1% HEPES buffer at 37 °C in 5% CO_2. After 24 h, the media was replaced with fresh media containing Platin-L (10 μM). After 24 h of incubation, the media was removed and the cells were washed thrice using bicarbonate, glucose, and serum free DMEM, supplemented with 1% glutamine and incubated in a non-CO_2 incubator at 37 °C for an hour. Glycolytic stress test was run using glucose, 10 mM, Oligomycin, 1 μM and 2-Deoxyglucose, 50 mM in ports A, B, and C, respectively using Seahorse XF^{e}96 Extracellular Flux Analyzer.

Fuel Flex Assay

PC3, LNCaP, DU145 and 22RV1 cells were seeded in 96-well XFe96 Seahorse microplates at a density of 20,000 cells/ well in 100 μL RPMI-1640 supplemented with 10% FBS, 1% penstrep, 1% sodium pyruvate, 1% HEPES buffer and grown for 12 h. HePG2 cells were seeded in 96-well XFe96 Seahorse microplates at a density of 20,000 cells/ well in 100 μL DMEM supplemented with 10% FBS, 1% penstrep, 1% sodium pyruvate, 1% glutamine and grown for 12 h. Subsequently the media was removed again, and the cells were washed with Seahorse basal media three times and incubated at 37 ºC in a non-CO_2 incubator. Fuel flex assay for the different fuel pathways viz. glucose, glutamine and fatty acid was studied by measuring the basal oxygen consumption rates and that after addition of the inhibitor of the target pathway in port A and a mixture of the inhibitors of the other two pathways in port B. This gave a measure of the dependency of the cells on a particular fuel pathway. To study the capacity of a certain fuel pathway, the sequence of addition of the inhibitors was reversed. In port A was added the mixture of inhibitors for the other pathways and in port B was added the inhibitor for the target pathway. UK-5099 (pyruvate dehydrogenase inhibitor, 20 μM) was used as an inhibitor for the glucose pathway. BPTES (selective inhibitor of Glutaminase GLS1, 30 μM) was used as an inhibitor for the glutamine pathway. Etomoxir (O-carnitine palmitoyltransferase-1 (CPT1A) inhibitor, 40 μM) was used as an inhibitor for the fatty acid pathway.

Exogenous and Endogenous Fatty Acid Oxidation

PC3 and LNCaP cells were plated at a density of 20,000 cells per well in 96-well Seahorse microplates in 100 μL RPMI-1640 supplemented with 10% FBS, 1% sodium pyruvate, 1% penicillin/streptomycin and 1% HEPES buffer and grown for 12 h. The media was removed and replenished with 100 μL fresh media. Cisplatin (10 μM), Platin-4 (10 μM) or Platin-L (10 μM) were added to the cells and incubated for 24 h. The media was removed and 200 µL of substrate limited media (RPMI: 0.5 mM glucose,1 mM Glutamine, 0.5 mM, carnitine, and 1 % FBS) was added to the cells and incubated for another 24 h. The cells

were washed thrice with FAO assay medium (KHB: (111 mM NaCl, 4.7 mM KCl, 1.25 mM $CaCl_2$, 2 mM, $MgSO_4$, 1.2 mM NaH_2PO_4) supplemented with 2.5 mM glucose, 0.5 mM carnitine, and 5 mM HEPES on the day of the assay, adjusted to pH 7.4 at 37 °C) and incubated at 37 ºC for 30 mins in a non-CO_2 incubator. Fifteen minutes prior to starting the assay (t = -15 minutes), etomoxir was diluted in FAO assay medium was added to the cells such that the final concentration was 40 μM and incubated for 15 minutes at 37 °C in a non-CO_2 incubator. Just prior to starting the assay (t = 0 minutes), 30 μL XF-Palmitate-BSA FAO Substrate or BSA was added to the appropriate wells. A Mito Stress assay was run using oligomycin (1 μM), FCCP (1 μM), and a mixture of Antimycin-A/ Rotenone (1 μM) each in ports A, B, and C respectively using Seahorse XFe96 Extracellular Flux Analyzer.

Fatty Acid Dependency using different Pt(IV) Prodrugs
PC3 or LNCaP cells were seeded in 96-well XFe96 Seahorse microplates at a density of 20,000 cells/ well in 100 μL RPMI-1640 supplemented with 10% FBS, 1% pen/strep, 1% sodium pyruvate, 1% HEPES buffer and grown for 12 h. Subsequently the media was removed, and the cells were washed with Seahorse basal media three times and incubated at 37 ºC in a non-CO_2 incubator. The port A of the previously hydrated cartridge was loaded with either etomoxir (final concentration = 40 μM), or various Platin-X compounds (Platin-A, Platin-B, Platin-Az, Platin-M, Platin-4, Platin-L, or chemotherapeutics like cisplatin, oxaliplatin or docetaxel (final concentration = 100 μM of each component) and port B was loaded with a mixture of UK5099 and BPTES (20 and 30 μM respectively). The basal oxygen consumption rates of the cells and after injections were measured using a Seahorse XFe96 extracellular flux analyzer.

Optimization of Concentration of Platin-L
LNCaP cells were seeded in 96-well XFe96 Seahorse microplates at a density of 20,000 cells/ well in 100 μL RPMI-1640 supplemented with 10% FBS, 1% pen/strep, 1% sodium pyruvate, 1% HEPES buffer and grown for 12 h. Subsequently the media was removed, and the cells were washed with Seahorse basal media three times and incubated at 37 ºC in a non-CO_2 incubator. The ports A and B of the previously hydrated cartridge was loaded with etomoxir (final concentration = 40 μM) and Platin-L (final concentration = 50, 100, 200 μM) or vice versa. The basal oxygen consumption rates of the cells and after injections were measured using a Seahorse XFe96 extracellular flux analyzer.

Fatty Acid Dependency studies
PC3 or LNCaP cells were seeded in 96-well XFe96 Seahorse microplates at a density of 20,000 cells/ well in 100 μL RPMI-1640 supplemented with 10% FBS, 1% pen/strep, 1% sodium pyruvate, 1% HEPES buffer and grown for 12 h. Subsequently the media was removed, and the cells were washed with Seahorse basal media three times and incubated at 37 ºC in a non-CO_2 incubator. The ports A and B of the previously hydrated cartridge was loaded with etomoxir (final concentration = 40 μM), cisplatin, lauric acid, mixture of cisplatin and lauric acid, Platin-L or docetaxel (final concentration = 100 μM of each component) respectively. The basal oxygen consumption rates of the cells and after injections were measured using a Seahorse XFe96 extracellular flux analyzer.

Fatty Acid Dependency Studies with or without Fatty Acid Synthase (FASN) inhibitor

LNCaP cells were seeded in 96-well XFe96 Seahorse microplates at a density of 20,000 cells/ well in 100 µL RPMI-1640 supplemented with 10% FBS, 1% pen/strep, 1% sodium pyruvate, 1% HEPES buffer and grown for 12 h. The media was replenished with fresh media (200 *µ*M) and cells were either treated with cerulenin (20 *µ*M) or incubated as such for 24 h. Subsequently the media was removed again, and the cells were washed with Seahorse basal media three times and incubated at 37 ºC in a non-CO_2 incubator. The ports A, and B of the previously hydrated cartridge was loaded with etomoxir (final concentration = 40 *µ*M), Platin-4 or Platin-L (final concentration = 100 *µ*M) or vice versa. Ports C and D were loaded with FCCP (1 *µ*M) and Antimycin-A/rotenone (1 *µ*M each). The basal oxygen consumption rates of the cells and after injections were measured using a Seahorse XFe96 extracellular flux analyzer.

Fatty acid dependency studies with external substrate with or without fatty acid synthase (FASN) inhibitor.

LNCaP cells were seeded in 96-well XFe96 Seahorse microplates at a density of 20,000 cells/ well in 100 *µ*L RPMI-1640 supplemented with 10% FBS, 1% pen/strep, 1% sodium pyruvate, 1% HEPES buffer and grown for 12 h. The media was replenished with fresh media (200 *µ*M) and cells were either treated with cerulenin (20 *µ*M) or incubated as such for 24 h. Subsequently the media was removed again, and the cells were washed with Seahorse basal media three times and incubated at 37 ºC in a non-CO_2 incubator. Port A of the previously hydrated cartridge was loaded with either the vehicle, etomoxir (final concentration = 40 *µ*M), or Platin-L (final concentration = 100 *µ*M). Port B was loaded with Palm-BSA conjugate (22 *µ*L), port C was loaded with vehicle, etomoxir (final concentration = 40 *µ*M), or Platin-L (final concentration = 100 *µ*M) and port D was loaded with FCCP (1 *µ*M).

Generation of PC3 *CPT1A^{KO}* using CRISPR/Cas9 transfection

PC3 cells were seeded at a density of $0.4x10^6$ in three wells of a 6 well plate and allowed to grow for 18 h. The lenti virus was prepared according to the procedure from Origene. Briefly, 1 *µ*g of guide RNA (gRNA) or scramble control is added to 250 *µ*L of OPTI-MEM and vortexed gently. To this solution, 1 *µ*g of DNA was added and then the solution was again vortexed gently. Then, 8 *µ*L of turbofectin 8.0 is added to the mixture and it is mixed by gently pipetting the solution. This is incubated at RT for 15 minutes. The OPTI-MEM solution is then added dropwise into the well containing cells. 48 h later, the cells are split 1:10. Following this, the cells are split at confluency 7 times. The cells undergo a puromycin screen, till a final concentration of 1 *µ*g/mL to eliminate cells that did not undergo transfection of the plasmid. The cells are allowed to grow, and the transfection is also checked through confocal microscopy to verify the transfection of the plasmid. Finally, knockout of the target gene is confirmed with through Western blot analysis.

Isolation of Cellular Organelles

Cells (PC3 and PC3 *CPT1A*^{KO}) were seeded at a density of $1x10^7$ cells in T-175 cell culture flasks and were allowed to grow to near 100% confluency. The cells were then incubated with 50 μg/mL of Platin-L or 50 μg/mL of cisplatin for 0.5 h, 1 h and 3 h respectively. Cells were then trypsinized and collected in centrifuge tubes pre-incubated on ice and sub-cellular fractionation was carried out using reagents from a mitochondria isolation kit for mammalian cells purchased from Thermo Fisher Scientific (Catalog number: 89874). Reagent A with protease inhibitors (10 mg/mL) was added and the cells were incubated on ice for 2 min. Reagent B was added and incubated on ice for 5 mins with vertexing at maximum speed every minute. Reagent C was then added and gently mixed and centrifuged at 700 x g at 4 °C for 10 mins. The supernatant contains the cytoplasmic and mitochondrial fraction, and the pellet contained the nuclear fraction. The supernatant was further purified using reagent C and centrifuging at 12000 x g at 4 °C for 15 mins. The resulting pellet contained the isolated mitochondria, and the supernatant contained the cytoplasmic fraction. Similarly for test articles uptake study, PC3 cells were plated at a similar density in T175 flasks and allowed to grow. Test articles namely 10 μM of Platin-L, 10 μM of cisplatin, 10 μM of Platin-L-4, and 10 μM of Platin-4 were added to cells and incubated for 3 h at 37 °C, 5% CO_2. Mitochondrial and cytoplasmic fractions were in a similar manner and the mitochondrial fractions were dissolved in 100 μL of RIPA. The isolated nucleus fraction was further purified into nuclear and cellular debris fraction. The pellet was dissolved in 600 μL of modified Tris-HCl buffer (10 mM Tris-HCl of pH 7.0, 10 mM NaCl, 3 mM $MgCl_2$, and 30 mM sucrose). The solution was incubated in ice for 10 mins and centrifuged at 3000 rpm, 4 °C for 10 mins. The supernatant was the debris fraction and was stored in separate Eppendorf tubes. The resulting pellet was resuspended in pre-chilled $CaCl_2$ buffer (10 mM Tris-HCl of pH 7.0, 10 mM NaCl, 3 mM $MgCl_2$, 10 mM $CaCl_2$, and 30 mM sucrose). The washing was repeated twice, and the supernatant was discarded. Finally, the pellet was further purified in resuspending buffer (20 mM Tris-HCl of pH 7.9, 20% glycerol, 0.1 M KCl and 0.2 mM EDTA) and centrifuged at 14000 rpm for 30 mins at 4 °C. The pellet was dissolved using RIPA and the protein content of the individual fractions was quantified using BCA assay and the Pt content of each fraction was quantified by ICP-MS. Statistical analysis was done using one-way ANOVA.

Immunoprecipitation of CPT1A in PC3 cells

PC3 cells were plated in T-175 flasks at 2.1 x 10^6 cells/flask and grown for 24 hours. On the same day, 25 μL of magnetic beads (Thermo Fischer, Cat# 26162) were prepared by incubating with 5 μg of anti-CPT1A antibody (Abcam, ab220789) at 4 °C, rotating overnight for each treatment condition. Vehicle control or articles (cisplatin or platin-L) were then added at 50 mM concentration for 6 h. Cells were washed with 1X PBS twice to remove all media and were then scraped off the flask using cell scrapers, and the cell pellets were obtained by centrifuging at 1800 x g for 5 mins in PBS. The pellets were lysed in buffer A (0.8% Triton X-100, 20 mM Tris-HCl pH7.9, 10 mM KCl, 1.5 mM $MgCl_2$, 0.5 mM 4-(2-aminoethyl)benzenesulfonyl fluoride hydrochloride (AEBSF)) with 150 mM NaCl on ice for 10 mins, followed by probe sonication for 5 seconds. The samples were then centrifuged at 15000 rpm at 4 °C for 15 min and the supernatants were collected. The prepared magnetic beads were added to the individual supernatants and incubated

at 4 °C, rotating overnight. The next day, the magnetic beads were separated using a magnetic stand, and the supernatant was removed. The beads were washed for three minutes, three times with buffer A. Isolated magnetic beads were resuspended in 50 μL of buffer A, and confirmation was carried out by western blot. Pt content was measured using ICP-MS. Statistical analysis was done one-way ANOVA.

Western Blot Analyses

The protein content and cell lysates and immunoprecipitated samples were quantified using bicinchoninic acid (BCA) assay. A 40 μg portion of protein was used in each well along with 1X Laemmli sample buffer and the gel was run at 100 mV for 2 h at room temperature. The resolved proteins were transferred onto a PVDF membrane at 50 mV at 4 °C for 2 h. The membrane was blocked for 1 h at room temperature in blocking buffer made with 5% nonfat dry milk powder in tris buffer saline TWEEN-20 buffer (TBST, TRIS = 2.42 g and NaCl = 8.0 g in 1 L of DI water at pH of 7.6; TWEEN20 = 1:1000 dilution). The membrane was kept at 4 °C overnight for primary antibody incubation. Primary antibody was made in 5% nonfat dry milk solution. Respective antibodies at a concentration of 1:1000 (CPT1A, β-actin, PSMA, beta catenin, calnexin, Hsp60, histone H3, TFAM) were used. The next day, the membrane was washed 5 times with TBST buffer and then incubated with respective HRP conjugated secondary antibodies (1:5000 dilution in 5% nonfat dry milk in TBST) at room temperature for 1 h. The membrane was again washed 5 times with TBST buffer before developing with ECL. The images were taken using a BioRad ChemiDoc imaging system. The bands in the membrane were quantified using the ImageJ.

Cellular Uptake Study using BODIPY-Palmitate

Human prostate cancer PC3 or CPT1A^{KO} cells were plated at a density of 10,000 cells per dish in a live cell imaging dishes in RPMI media supplemented with 10% FBS, 1% Penicillin/Streptomycin, 1% HEPES and 1% sodium pyruvate and cells were incubated at 37 °C with 5% CO_2 for 24 h. The cells were treated with cisplatin, etomoxir, Platin-4, Platin-L-4 or Platin-L (all compounds = 10 μg/mL) for 3 h. After incubation the cells were washed thrice with PBS. These cells further incubated with 5 μL of 2 mM BODIPY-Palmitate in 1 mL of media for 5 min at 37 °C and finally was washed thrice with PBS. Finally, the cells were washed thrice with PBS and phenol red free media (2 mL/ dish) was added. Live cell images were recorded using a confocal microscope using 488/512 nm for BODIPY-Palmitate. All the images were acquired at same LASER power and exposure times.

CPT1 Enzymatic Activity Assay in PC3 cells

PC3 cells were plated at a density of 1 x 10^6 cells/flask and grown overnight in culture medium. Following day cells were treated with test articles at a concentration of 10 μM. Etomoxir was treated at 10 μM whereas Malonyl-CoA was treated with 10 μM and 80 μM. The incubation time for all the test articles was for 3 h. Cells were then trypsinized and the protein lysates were prepared in buffer A (0.8% Triton X-100, 20 mM Tris-HCl pH7.9, 10 mM KCl, 1.5 mM $MgCl_2$, 0.5 mM 4-(2-aminoethyl)benzenesulfonyl fluoride hydrochloride (AEBSF) with 150 mM NaCl. The protein lysates were quantified using BCA assay. The DTNB (5,5'- dithiobis(2-nitrobenzoic acid) buffer (116 mM Tris-HCl, pH 8.0,

0.09% Triton X-100, 1.1 mM EDTA, 0.12 mM DNTB) was mixed with the cell lysates and incubated at room temperature for 30 min in separate wells of a 96-well plate. The absorbance at 405 nm was recorded which served as the background. Then 100 μM of palmitoyl-CoA prepared in water and 5 mM L-carnitine prepared in 1 M Tris pH 8.0 were added to each well. After incubation of 20 min at 37 °C, the absorbance at 405 nm was recorded again. The difference between the readings with and without the substrates was normalized with protein concentration. CPT1A activity was defined as millimoles of CoA-SH released per milligram of protein.

Biodistribution of Intravenously Administered Platin-L in Normal Mice

BALB/c Albino male mice (N = 3) were injected with 1.2 mg/kg of Platin-L through tail IV. After 24 h, the mice were sacrificed, and the organs were collected including the brain, lungs, heart, liver, kidneys, spleen and testes. Organs were weighed and then dissolved in freshly prepared aqua regia. The solutions were then analyzed via ICP-MS for platinum content.

Synthesis of T-Fc-GLU-Platin-L-NPs

The Platin-L loaded nanoparticles (NPs) were made using either PLGA-*b*-PEG_{6000}-MAL or PLGA-*b*-PEG_{3500}-GLU, or a combination of the two (dual-targeted NP). A solution of the polymer(s) (total concentration of polymer = 10 mg/mL) and Platin-L (2 mg/mL, 20 % feed with respect to polymer construct) was made in 1 mL of DMF. The polymer and drug solution were added dropwise to 10 mL of deionized water with constant stirring (900 RPM) at room temperature and stirred for 2 h. The NPs were washed 3 times with nanopure water with amicon ultracentrifugation filtration membranes with a molecular weight cutoff of 100 kDa (2800 rpm, 4 °C). The formed NPs were suspended in water and stored at 4 °C. The NP size (diameter, nm), PDI, and surface charge (zeta potential, mV) were obtained from three independent measurements. Amounts of encapsulated Pt(IV) prodrug inside the polymeric nanoparticles were quantified by measuring the contents of Pt by ICP-MS. The morphology of the NPs was evaluated using TEM. NPs at a concentration of 10 mg/mL with respect to the total polymer were diluted 100 times using nanopure water. This NP solution (1 mL) was mixed with a 4% solution of uranyl acetate solution (10 μL) and gently vortexed. This solution was filtered with a 0.45-μm filter and ~20 μL was dropped on the dark side of a copper grid and allowed to dry for 24 h in a desiccator at room temperature.

Conjugation of Fc Immunoglobulin Fragment to Nanoparticle

Using the same process as in previous syntheses of our Platin-L loaded NPs, polyclonal IgG Fc fragments were covalently conjugated to nanoparticles made of PLGA-*b*-PEG-Mal and/or PLGA-*b*-PEG-GLU using thiolene chemistry. 2-Iminothiolane (4.5 μL from a 5 mg/mL solution in 5 mM EDTA) was mixed with 35 μL of Fc fragment (2.48 mg/mL) at 37 °C for 1 h. This mixture was added to the NP solution (10 mg/mL) and allowed to react at 4 °C for 1 h. The NPs were purified in a similar fashion as mentioned before and the change in diameter and zeta potential of the nanoparticles was studied using DLS. The amount of Fc was quantified using BCA assay. TEM images were recorded using JEOL JEM-1400 instrument.

Release Kinetics of Platin-L from Nanoparticles

The release of Platin-L from the targeted nanoparticles, T-GLU-Platin-L-NP, T-Mal-Platin-L-NP, T-Fc-Platin-L-NP, T-Mal-GLU-Platin-L-NP, and T-Fc-GLU-Platin-L-NP was studied using 1X PBS at physiological pH 7.4. The nanoparticle solutions were prepared by diluting five times with nanopure water. Then, 200 μL of the diluted solution was added to 500 μL dialysis tubes with MWCO of 10,000. These tubes were then submerged in PBS and kept in the shaking incubator at 37 °C up to 72 h. For the first 6 h, buffer was replenished with fresh buffer every hour and later on changed every 12 h. The samples were collected at predetermined time points and dissolved in dimethylformamide and analyzed by ICP-MS for remaining Pt concentrations. The drug release concentrations were remaining in the samples was quantified by using Pt standards, and these values were subtracted from the concentration of drug that was initially added to the dialysis tubes.

Biodistribution of T-Fc-GLU-Platin-L-NP

The distribution T-Fc-GLU-Platin-L-NPs was carried out in the tumor bearing mice from LNCaP xenograft experiment. A randomization study was conducted to assign the groups. Saline and T-Fc-GLU-Platin-L-NPs. Mice were administered orally with T-Fc-GLU-Platin-L-NP at a dose of 10 mg/kg with respect to Platin-L or saline (200 μL) biweekly for 4-weeks. On the terminal day of the experiment, when the tumor reached to the maximum volume, under anesthesia the blood was collected by cardiac puncture in heparin tubes, followed by perfusion with PBS at the flow rate of 7.5 mL/min. After perfusion, the animals were sacrificed, and all organs including tumor were harvested. The collected blood was centrifuged at 5000 RPM for 20 min to isolate the plasma. The collected organs were weighed and homogenized using aquaregia. The tissues were dissolved in the aquaregia for 2 days. Further, ICP-MS was performed to determine the Pt amount in all the organs, tumor tissue, and blood. The biodistribution of Platin-L was expressed as %injected dose and %injected dose/g of tissue.

Efficacy of NPs in LNCaP^{luc} Xenograft Model

Generation of Luciferase tagged LNCaP cells: Two wells of a 24-well plate LNCaP WT cells were seeded at a density of 2.5 x 10^5 cells/well. The cell plate was incubated overnight at 37 °C, 5% CO_2. A solution with the luciferase lentiviral particles (GeneCopoeia, Inc.) was prepared at a ratio of 1:5 of cells to viral particles with 1 μL of polybrene. The above solution of the lentiviral particles was prepared in 500 μL of RPMI growth media which was then added to the cells. This was followed by an incubation for 24 h at 37 °C, 5% CO_2. Post-transfection, the media was replenished with fresh growth media and the cells were allowed to become confluent. Once the cells reached 90% confluency, they were split into three T25 culture flasks and were allowed to grow until they became 80% confluent. Finally, the cells were exposed to the puromycin selection media (3 μg/mL) and allowed to grow for few passages until no cell death was observed. After 3 weeks of keeping the cells under puromycin selection, there was no cell death observed and the luciferase expression in the cells was confirmed by IVIS imaging.

Design and Development of LNCaP xenograft mouse model: For injection into male BALB/c nude mice, cultured cells were trypsized from tissue culture flasks and were washed with phosphate-buffered saline without Ca^{2+} or Mg^{2+} (PBS), cell number and viability was determined using trypan blue. Finally, 5 x 10^6 cells were taken in 50 μL of 1X PBS and mixed with 50 μL of Matrigel solution. This solution of cells was injected subcutaneously using a 27.5 gauge needle in the right flank of the mice. Mice were observed regularly, and tumor growth was monitored. It took about 11 weeks after cell inoculation to observe semi-solid palpable tumors at the inoculated area and their size was measured twice a week using a caliper. The length (L) and width (W) were measured. Each tumor volume measurement was repeated three times for verification. The diameters, areas, and volumes of all tumors were calculated using the formula (L*W*W)/2. We started dosing the NPs when the tumors reached ~200 mm^3. A randomization study was conducted to assign the groups. Mice were administered orally with T-Fc-GLU-Platin-L-NP at a dose of 10 mg/kg with respect to Platin-L or saline (200 μL) biweekly for 4-weeks. Tumor volumes and body weight were measured at the same time and finally the tumor weights were subtracted from the body weight of the mice. Once the mice reached to the maximum tumor volume, they were sacrificed. Under anesthesia, around 300 μL of blood was collected in heparin tubes by cardiac puncture, followed by perfusion of 1X PBS at 7.5 mL/min flow. The blood was further centrifuged at 5000 RPM for 20 min to collect the plasma. All the organs including the tumor tissue were dissected into two halves, out of which, one half was immediately snap frozen for further analyses and the other half was immersed in 4% paraformaldehyde (PFA) and submitted for tissue sectioning and H&E staining. The section, which was snap frozen on the terminal day, was further used for immunostaining, and RT-PCR analysis studies.

Development of Cisplatin Resistant LNCaP Xenograft Model

Inoculation of 2 x 10^6 LNCaP cells was done into the right flank of male BALB/c nude mice of age ~6 weeks. Tumors were observed on Day 52 after cell inoculation. Cisplatin treatment at a dosage of 3 mg/kg, was carried out twice a week for 3 and half weeks from day 62 post cell inoculation. Post cisplatin treatment, the mice were randomly divided into 3 groups: saline (n=4), cisplatin (n=5), and T-Fc-GLU-Platin-L-NP (n=5). The saline group did not receive any prior cisplatin treatment. Cisplatin was injected *via* tail iv at a dosage of 3 mg/kg twice weekly. Saline and T-Fc-GLU-Platin-L-NPs were administered *via* oral gavage at a dosage of 10 mg/kg with respect to Platin-L for 5 consecutive days in a week. Tumor volume and body weights were monitored regularly. The animals were also observed for any abnormalities. This was carried out for 11 weeks. At the terminal day of the study, the animals were euthanized, and the organs and tumor tissues were harvested for further analyses.

Quantitative PCR studies

The tissue sections were dissolved in 5 mL of TRIzol. The total RNA from each sample lysate was isolated using the manufacturer's protocol provided and was quantified using the nanodrop. Briefly, 350 μL of RLT buffer was added to each sample and incubated for 5 mins at room temperature. The samples were centrifuged for 3 mins at 14000 rpm and the supernatant was used for further purification steps. The supernatant was thoroughly mixed with 350 μL of 70% ethanol and transferred to a RNeasy mini spin column. The

samples were centrifuged for 15 sec at a speed ≥ 8000 x g. The flow through was discarded and steps for DNase digestion was performed. Prior to incubating the samples with DNase, I solution, the samples were washed with 350 μL of RW1 buffer at ≥ 8000 x g, 15 sec. For each sample 10 μL of DNase I was added to 70 μL of RDD buffer, mixed, and finally added on the spin column membrane. The samples were incubated for 15 mins at room temperature. Post incubation with DNase I solution, the samples were again washed with 350 μL of RW1 buffer and flow through was discarded. Finally, the samples were washed twice with 500 μL of RPE buffer which helps in removing any excess traces of salts from the samples. The RNA was eluted using RNase free water and collected in separate tubes. RNA was quantified using the nanodrop and used to synthesize cDNA using the 5X supermix from Bio-Rad. The cDNA was used to perform the RT-PCR in presence of primers for the different FAO genes, anti-apoptotic genes, using β-actin as control. The FAO primers: CPT1A_F 5'-CTCCGCCTGAGCCATGAAG-3'; CPT1A_R 5'-CACCAGTGATGATGCCATTC-3'; FASN_F 5'-CTTCCGAGATTCCATCCTACGC-3'; FASN_R 5'-TGGCAGTCAGGCTCACAAACG-3'. The β-actin primers: FH1_ACTB 5'-GACGACATGGAGAAAATCTG-3'; RH1_ACTB 5'-ATGATCTGGGTCATCTTCTC-3'. The anti-apoptotic primers: Survivin_F 5'-CCACTGAGAACGAGCCAGACTT-3' Survivin_R 5'-GTATTACAGGCGTAAGCCACCG-3'; BCL2_F 5'-ATCGCCCTGTGGATGACTGAGT-3' BCL2_R 5'-ATCGCCCTGTGGATGACTGAGT-3'; BCL-xL_F 5'-GCCACTTACCTGAATGACCACC-3' BCL-xL_R 5'-AACCAGCGGTTGAAGCGTTCCT-3' c-FLIP_F 5'-AGTGAGGCGATTTGACCTGCTC-3' c-FLIP-R 5'-CCTCACCAATCTCTGCCATCAG-3'

Immunohistochemistry of Tumor Tissue Sections

The slides were dewaxed by heating them at 60 °C for 10-15 mins. The wax was carefully wiped out and the slides were kept for rehydration in decreasing ethanol gradient. Incubation in xylene was carried out for 5 mins thrice followed by incubation in 100% ethanol twice for 10 mins each and then finally in 95% ethanol twice for 10 mins each. The slides were then washed with 1X TBS (tris buffer saline). The tissues are incubated in antigen retrieval buffer (2.94 g trisodium citrate in 1 L DI water with 500 μL Tween-20, pH = 6.0) for 20 mins in a decloaking chamber. The tissue sections were washed 3 times with water. The tissue sections on the slides were then subjected to permeabilization with 0.1% Triton-X-100 in 1X TBS for 10 mins at room temperature. The tissues were again washed 3 times with water and incubated in 3% H_2O_2 solution for 10 mins. The sections were then washed with 1X TBST (TBS with 0.1% Tween 20) thrice and blocked with blocking buffer for 1 h at room temperature. For all the antibodies 5% goat serum was used as the blocking buffer. Tissues were further incubated with the primary antibody solution in 5% goat serum in TBST (Cleaved caspase 3 at 1:400 dilution; caspase 9 at 1:1000 dilution; ERCC1 at 1:1000; PARP at 1:1000; Beta Catenin at 1:1000) in a humidifying chamber overnight at 4 ˚C. Tissues were washed with TBST twice at an interval of 5 mins and then incubated with HRP conjugated secondary antibody solution (1:20,000 dilution) in the humidifying chamber at room temperature for 1 h in dark. The sections were further washed thrice with 1X TBST buffer at an interval of 5 mins. Each tissue section was then covered with DAB signal strength solution and kept for 10 mins and finally washed with water. The slides were counter stained with hematoxylin for 30 sec at room temperature and immediately washed with tap water. The tissue sections

were then rehydrated again with increasing ethanol gradient. At each step the slides were kept in the solution for 20 secs. The immersion sequence is as follows: 95% ethanol, 100% ethanol, and xylene. Finally, the slides were mounted with a DPX Mountant and viewed under brightfield microscope.

Immunostaining of Tumor Sections

The tumor sections from the patient prostate tissues and the tumor tissues harvested from the mice were sectioned and embedded on glass slides using paraffin. These tissues were stained for PSMA and CPT1A. The slides with the paraffin embedded tissue sections were heated at 55-60 °C for 30 mins. The slides were then rehydrated in a gradually decreasing ethanol gradient. At each step the slides were kept in the solution for 10 mins. The immersion sequence is as follows: xylene, 100% ethanol, 95% ethanol, 80% ethanol, 70% ethanol, 60% ethanol, 50% ethanol, 40% ethanol, 30% ethanol, 20% ethanol, 10% ethanol, and water. The tissues are incubated in antigen retrieval buffer (2.94 g trisodium citrate in 1 L DI water with 500 μL Tween-20, pH = 6.0) for 20 mins in a decloaking chamber. The tissues were washed 3 times with 1X PBS. The tissue sections on the slides were then subjected to permeabilization with 0.1% Triton-X-100 in 1X PBS for 10 mins at room temperature. The tissues were again washed 3 times with 1X PBS and blocked with blocking buffer for 1 h at room temperature. For all the antibodies 5% goat serum was used as the blocking buffer. Tissues were further incubated with the primary antibody solution in 1% BSA (PSMA at 1:1000 dilution; CPT1A at the concentration of 5 μg/mL) in a humidifying chamber overnight at 4 ˚C. Tissues were washed with blocking buffer twice at an interval of 30 min and incubated with Alexa Fluor® 488 conjugated secondary antibody solution (1:200 dilution) for PSMA and Alexa Fluor® 488 conjugated secondary antibody solution (1:200 dilution) for CPT1A in the humidifying chamber at room temperature for 1 h in dark. Tissues were then washed thrice with 1X PBS followed by addition of DAPI and kept for 5 min at room temperature. The sections were finally washed with 1X PBS for 5 times and mounted on a glass slide using DPX mounting media. The slides were then imaged using Olympus FluoView FV3000 confocal microscope. The sampling speed for every image was kept as 8.0 μs/pixel (0.56 min per image). The immunofluorescence images showed the presence of the respective markers in the patient tissue samples. Quantification of the fluorescence of the proteins was carried out using ImageJ software. Statistical analysis was done by paired p-test. Immunofluorescence of whole mouse brain samples were carried out in similar way and stained for NGF marker. Whole brain section images were taken in VS-120 microscope.

(A)

Analyses of CPT1a in 21 Individuals with Acinar Adenocarcinoma in Prostate Gland				
**No.**	**Age**	**PSA (ng/mL)**	**Grade Group**	**Gleason Score**
1.	58	9.61	5	5+5 = 10
2.	63	5.87	5	4+5 = 9
3.	57	2.23	5	5+4 = 9
4.	66	5.68	5	4+5 = 9
5.	80	5.95	5	4+5 = 9
6.	80	8.3	5	4+5 = 9
7.	64	6.0	5	4+5 = 9
8.	75	4.4	3	4+3 = 7
9.	71	7.06	2	3+4 = 7
10.	76	18.7	2	3+4 = 7
11.	60	5.63	2	3+4 = 7
12.	67	13.9	2	3+4 = 7
13.	63	4.6	2	3+4 = 7
14.	56	2.47	2	3+4 = 7
15.	64	8.89	2	3+4 = 7
16.	75	7.74	2	3+4 = 7
17.	62	8.7	2	3+4 = 7
18.	38	6.0	2	3+4 = 7
19.	58	5.6	2	3+4 = 7
20.	70	5.7	1	3+3 = 6
21.	62	11.5	1	3+3 = 6

(B)

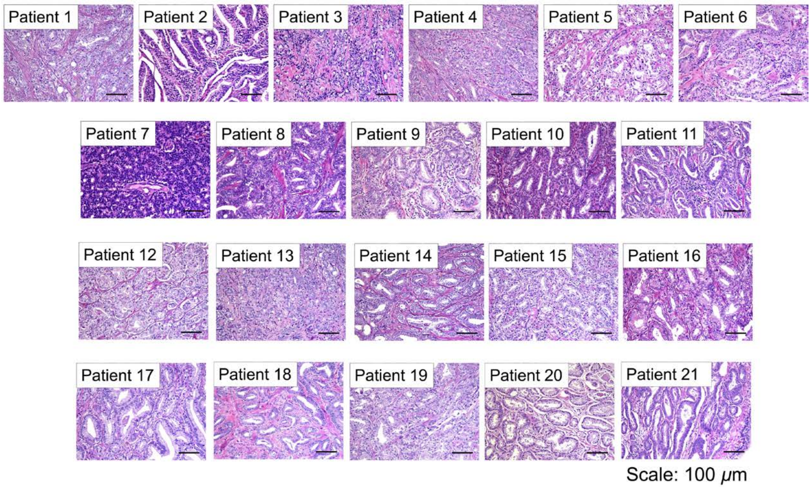

Fig. S1. Treatment naïve patient samples. 21 patient tumor sections were obtained and characterized. (A) Physical characteristics of the patient including the age, PSA level, cancer grade group and Gleason score. (B) H&E images of the cancerous regions of the tumor sections taken at 20x magnification. Scale bar = 100 μm.

(A)

Analyses of CPT1A in ADT Treated 17 Patients with Acinar Adenocarcinoma in Prostate Gland					
**Patient No.**	**Age**	**PSA (ng/mL)**	**Grade Group**	**Gleason Score**	**Treatment**
1	76	11.7	5	5+5 =10	Casodex
2	67	57.59	5	4+5 = 9	ADT (Monthly Firmagon)
3	74	27.4	5	4+5 = 9	Chemotherapy
4	68	4.3	5	4+5 = 9	Neoadjuvant ADT started 12/2019 and has had a total of 9 months ADT (Casodex)
5	67	19.9	5	4+5 = 9	Eligard x 2
6	75	0.57	5	4+5 = 9	Bicalutamide, Lupron
7	76	0.0575	5	4+5 = 9	Lupron , Enzalutamide
8	66	112.00	4	3+5 = 8	Casodex- Hormone Associated Therapy and Hormone Based Chemotherapy
9	67	119.8	4	4+4 = 8	Lupron
10	74	0.014	4	4+4 = 8	Lupron
11	61	25.80	3	4+3 = 7	ADT, Lupron
12	68	36.84	3	4+3 = 7	LHRH agonist/antagonist
13	70	6.20	2	3+4 = 7	Casodex- Hormone Associated Therapy and Hormone Based Chemotherapy
14	65	23.54	2	3+4 = 7	Eligard injection
15	69	14.51	2	3+3 = 6	Triptorelin injections, Bicalutamide
16	69	5.6	1	3+3 = 6	Dutasteride
17	76	6.5	1	3+3 = 6	Finasteride, Bicalutamide

(B)

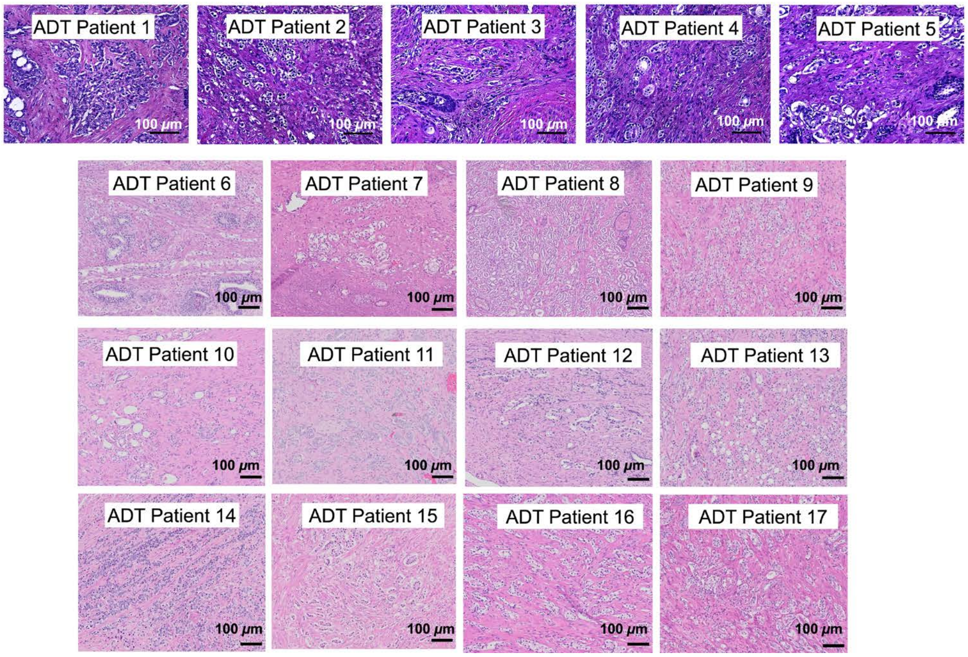

Fig. S2. Patient samples after receiving treatment. Characterization of 17 patient samples who had received treatment prior to tumor analysis. (A) Physical characteristics of the patient including the age, PSA level, cancer grade group, Gleason score and details on the type of treatment administered. (B) H&E images of the cancerous regions of the tumor sections. Images were taken at 20x magnification. Scale bar = 100 μm.

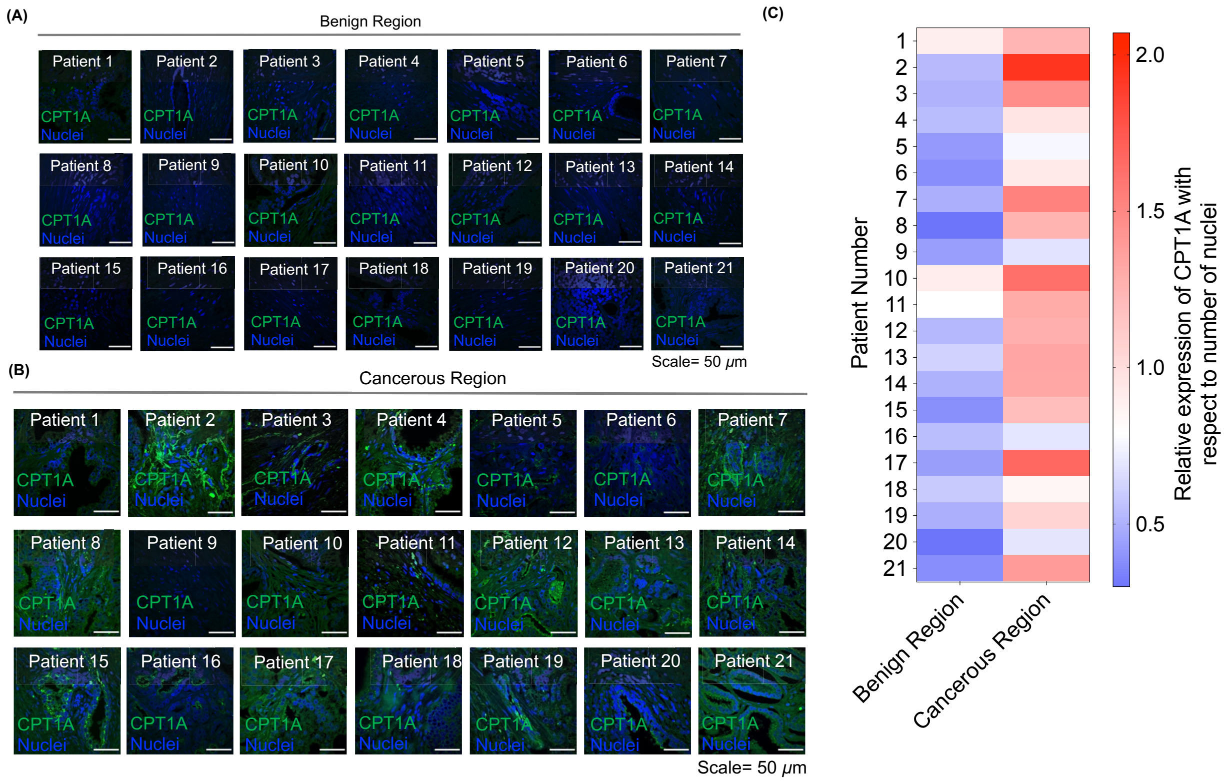

Fig. S3. CPT1A expression in treatment naïve patient samples. Expression of CPT1A in the tumor sections was analyzed through immunofluorescence in both benign (A) and cancerous (B) regions of the tumor section. (C) Expression of CPT1A with respect to the number of nuclei was calculated and expressed as a heat map. Three images were taken for each sample and the average expression of CPT1A was calculated with respect to the number of nuclei present in the image. Quantification was carried out using ImageJ software.

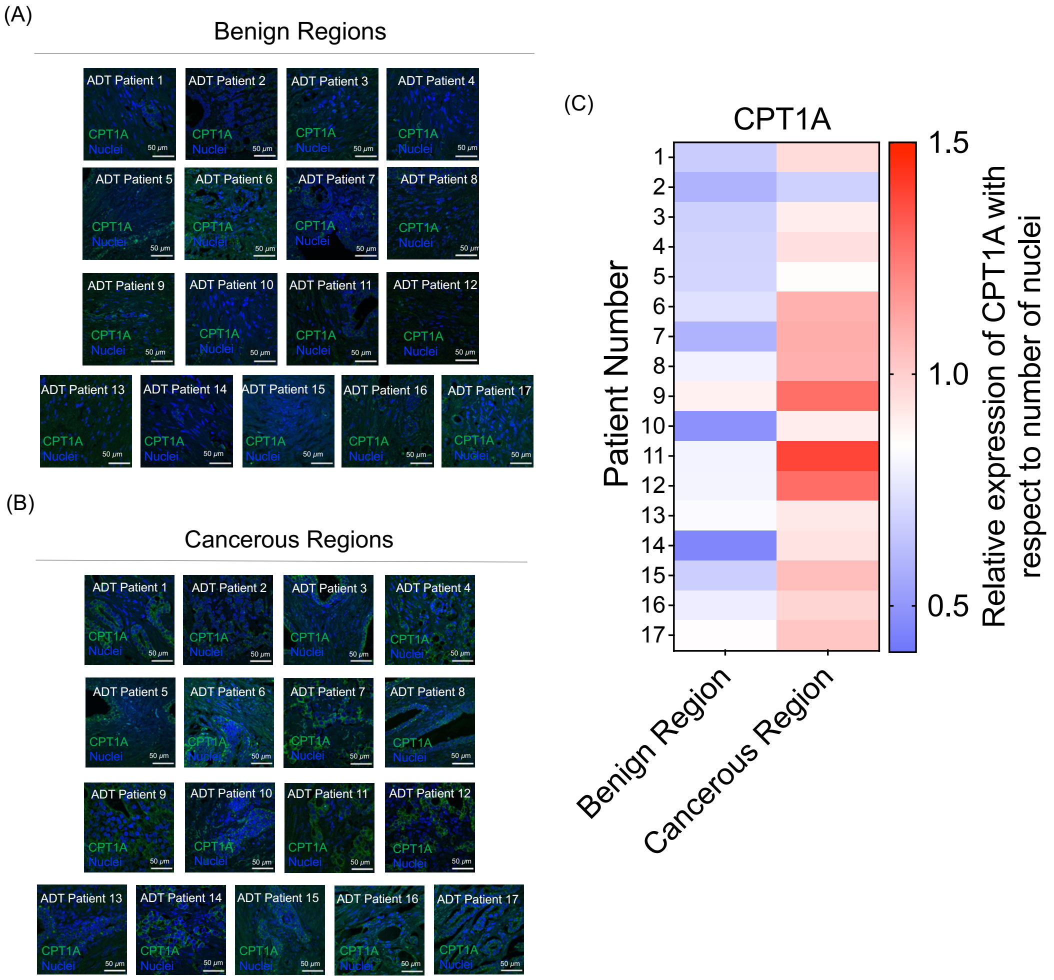

Fig. S4. CPT1A expression in ADT treated patient samples. Expression of CPT1A in the tumor sections was analyzed through immunofluorescence in both benign (A) and cancerous (B) regions of the tissue. (C) Expression of CPT1A with respect to the number of nuclei was calculated and expressed as a heat map. Five images were taken for each sample and the average expression of CPT1A was calculated with respect to the number of nuclei present in the image. Quantification was carried out using ImageJ software.

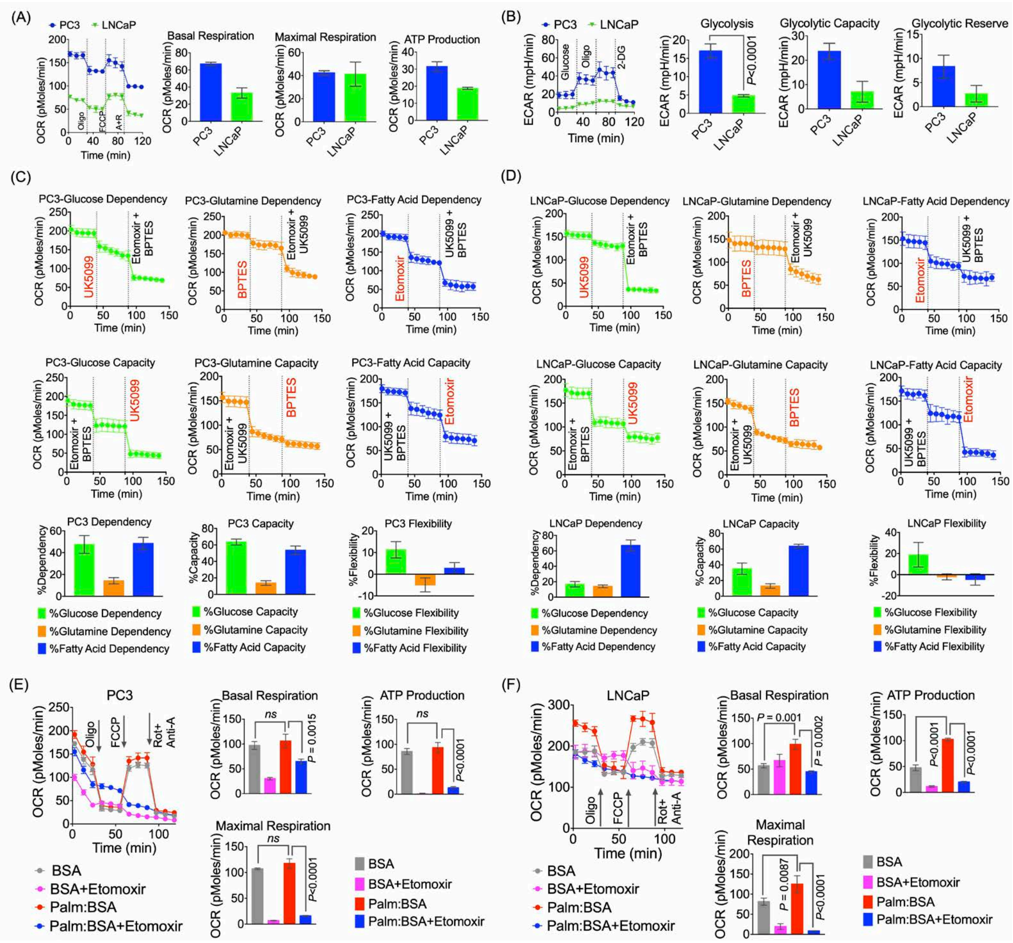

Fig. S5. (A) Mitochondrial respiration profiles of PC3 and LNCaP cells. Oligo-oligomycin, ATP synthase inhibitor; FCCP-carbonyl cyanide-p-trifluoromethoxyphenylhydrazone, an ionophore; Rot-rotenone, an inhibitor of mitochondrial complex I; and Anti-A-antimycin A, an inhibitor of mitochondrial complex III. (B) Glycolytic patterns of PC3 and LNCaP cells by evaluating extra cellular acidification rate (ECAR) of these cells using glucose, oligo, and an inhibitor of hexokinase II, 2-deoxy glucose (2-DG). Substrate utilization by (C) PC3 cells and (D) LNCaP cells for mitochondrial OXPHOS by using Seahorse Fuel Flex kit which includes varied addition sequence of UK5099, BPTES, and etomoxir to understand the contributions from glucose, glutamine, and fatty acids, respectively. Glucose dependency of cells was measured by using XF fuel flex optimization assay medium and injection of UK5099 and then simultaneous addition of etomoxir and BPTES; the injection order was also reversed to study the glucose capacity. The glutamine and fatty acid dependency and capacity were studied in a similar way as glucose pathway except the injection chemicals were varied. For glutamine dependency, BPTES, then etomoxir plus UK5099 were injected and reverse injection order was used to study the glutamine capacity. For fatty acid dependency, etomoxir, then BPTES plus UK5099 were injected and the reverse injection order was used to study the fatty acid. Simultaneous measurements of oxidation of exogenous and endogenous fatty acids by (E) PC3 and (F) LNCaP cells using the XF Palmitate-BSA FAO substrate. PC3 and LNCaP cells were grown in substrate-limited medium overnight to promote exogenous fatty acid utilization and then Palmitate-BSA FAO substrate was used to understand the effects of exogenous fatty acid on beta oxidation using Mito Stress Test. Etomoxir was used as an inhibitor of FAO to confirm the FAO driven respiration towards OCR.

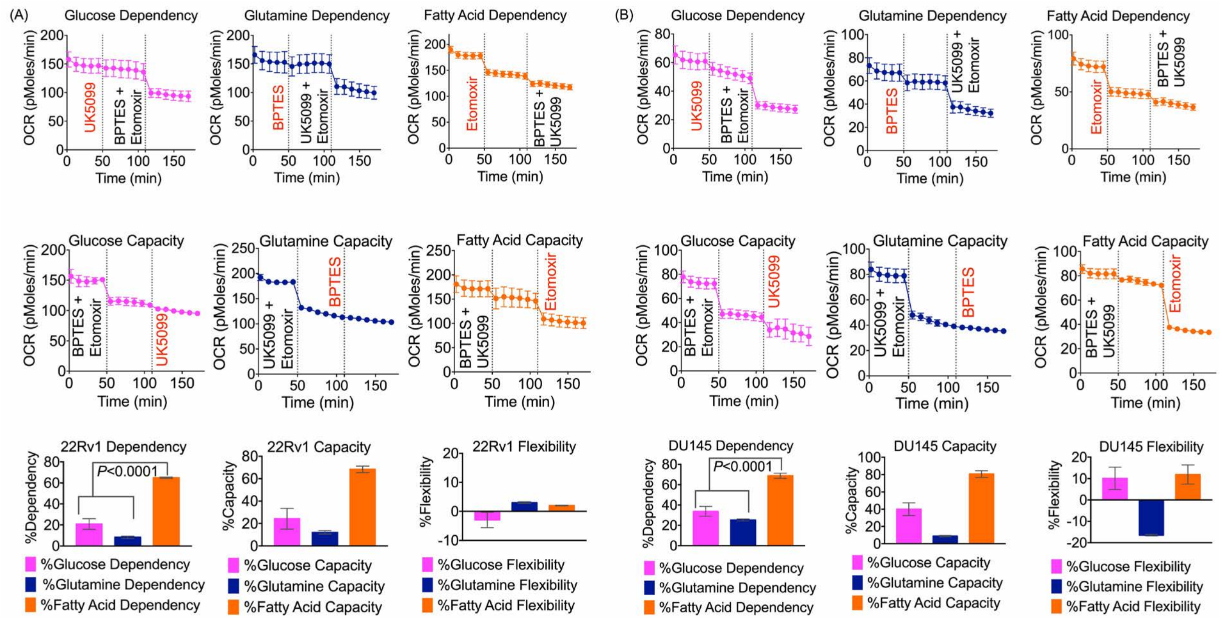

Fig. S6. Substrate utilization by (A) DU145 and (B) 22RV1 cells for mitochondrial OXPHOS using Seahorse Fuel Flex assay kit which includes varied addition sequence of UK5099, BPTES, and etomoxir to understand the contributions from glucose, glutamine, and fatty acids, respectively. Glucose dependency of cells was measured by using XF fuel flex optimization assay medium and injection of UK5099, then simultaneous addition of etomoxir and BPTES; the injection order was also reversed to study the glucose capacity. The glutamine and fatty acid dependency and capacity were studied in a similar way as glucose pathway except the injection chemicals were varied. For glutamine dependency, BPTES, then simultaneous addition of etomoxir plus UK5099 were injected and reverse injection order was used to study the glutamine capacity. For fatty acid dependency, etomoxir, then simultaneous addition of BPTES plus UK5099 were injected and the reverse injection order was used to study the fatty acid capacity.

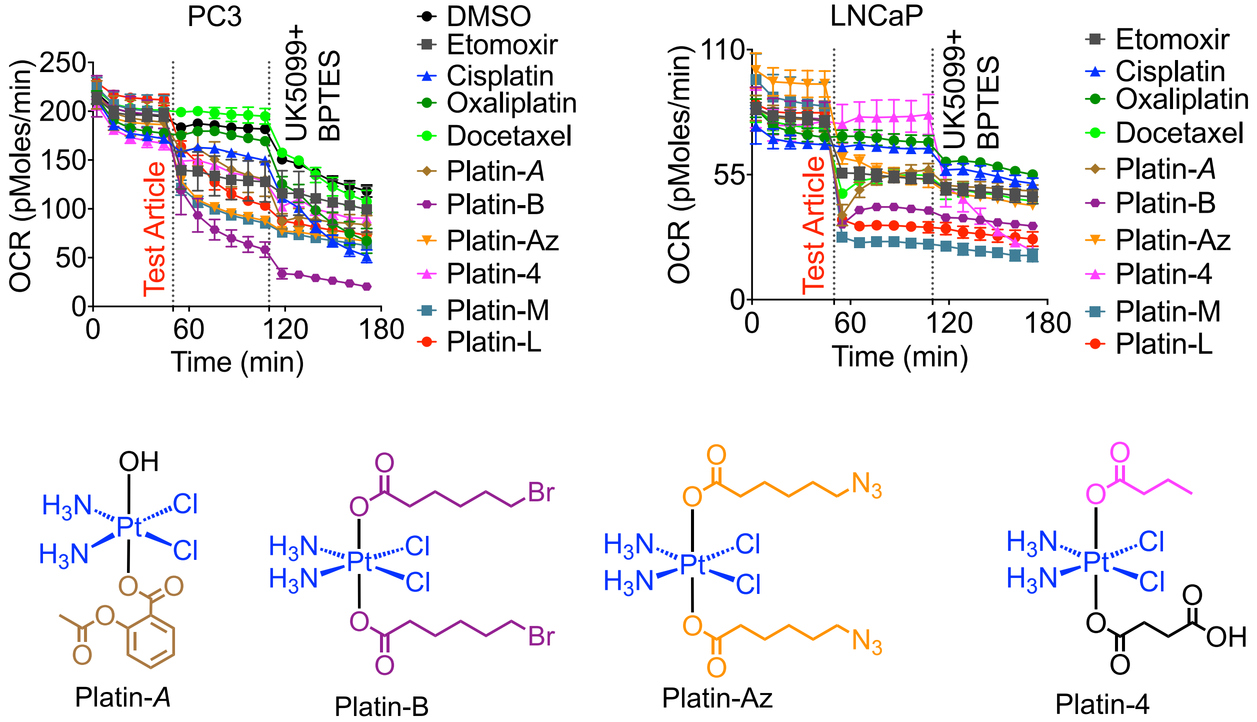

Figure S7. Effect of platinum prodrugs and common chemotherapeutic agents on affecting the fatty acid oxidation pathway in PC3 and LNCaP cells. A modified Seahorse Fuel Flex assay was used to study *the* effect of the compounds on the fatty acid oxidation pathway. Injection of the test article (final concentration 100 μM) was followed by the simultaneous injection of BPTES plus UK5099. Structures of the additional platinum prodrugs are given on the bottom.

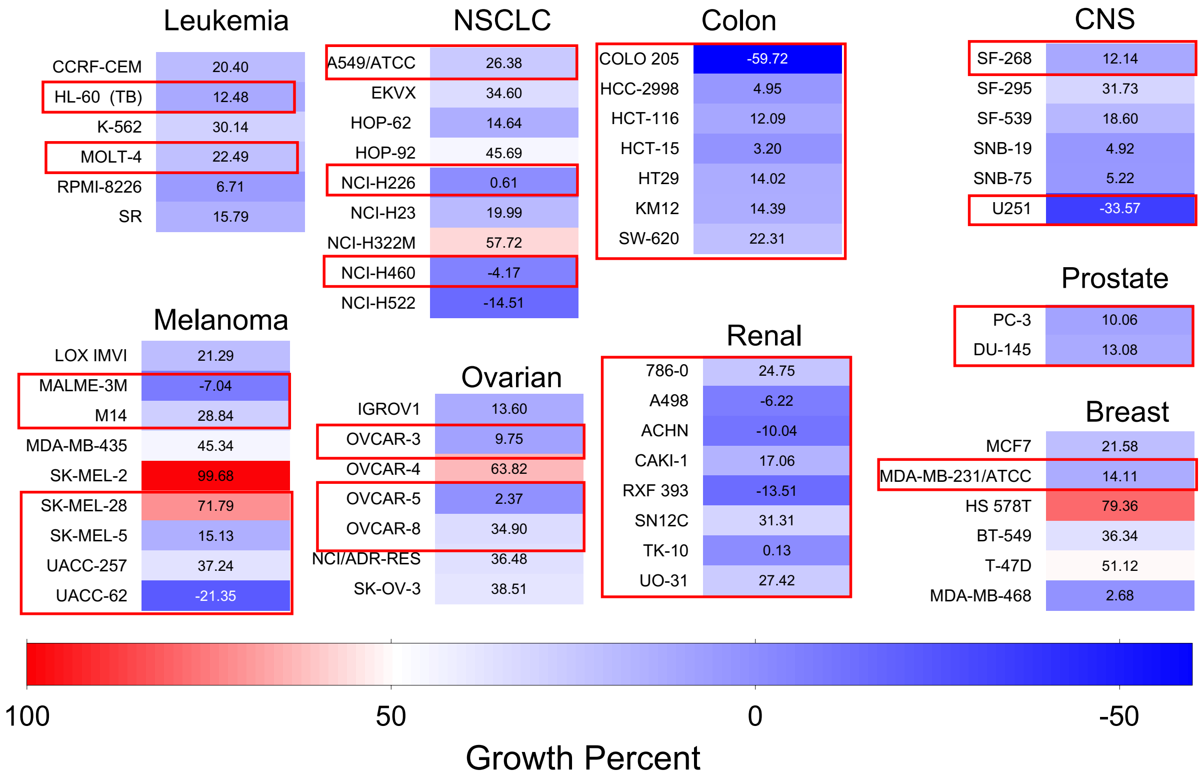

Fig. S8. Growth percent of NCI60 cell panel after single dose treatment with Platin-L at 10 μM. Several cell lines show growth percent of less than 50% after treatment with Platin-L. Cell lines with dysregulated lipid metabolism are highlighted in red boxes as suggested by literature or by our unpublished data. Scale: Color gradient change at 50% growth.

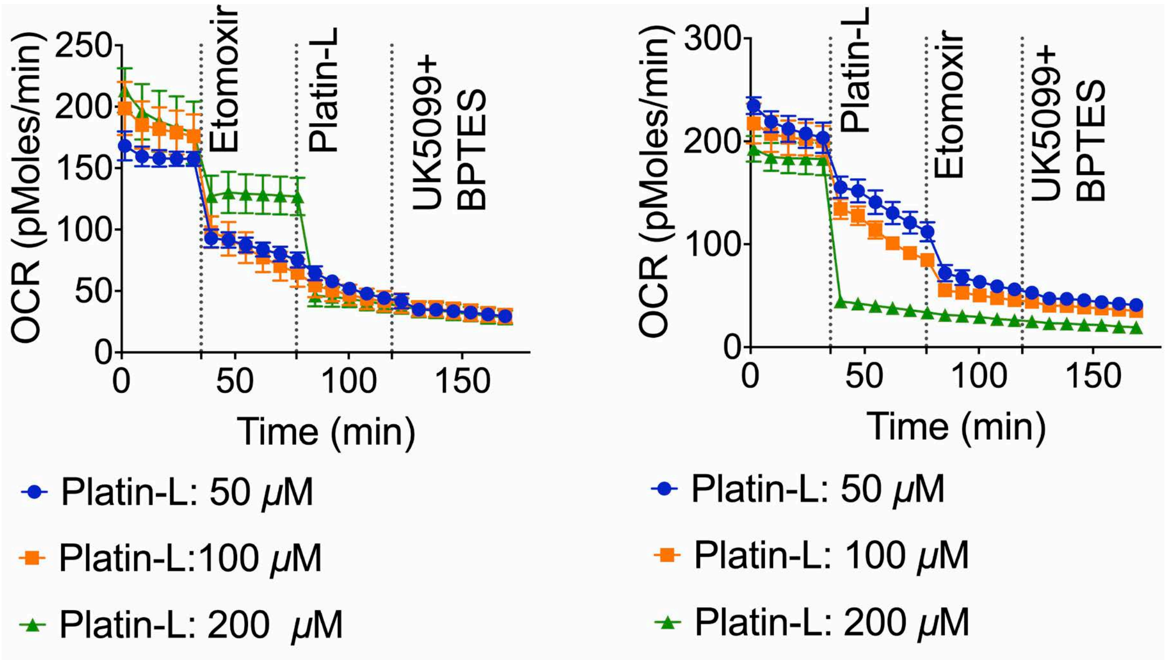

Fig. S9. Modulation of FAO pathway in presence of etomoxir or Platin-L at different concentrations in LNCaP cells. Real time oxygen consumption was monitored in response to addition of etomoxir, then Platin-L, then the simultaneous injection of UK5099 plus BPTES to evaluate the effect of Platin-L on the cell's response to etomoxir and vice-versa. Concentration of Platin-L was varied from 50 *μ*M to 200 *μ*M. Etomoxir, UK5099 and BPTES concentration were constant at 40 *μ*M, 20 *μ*M and 30 *μ*M respectively.

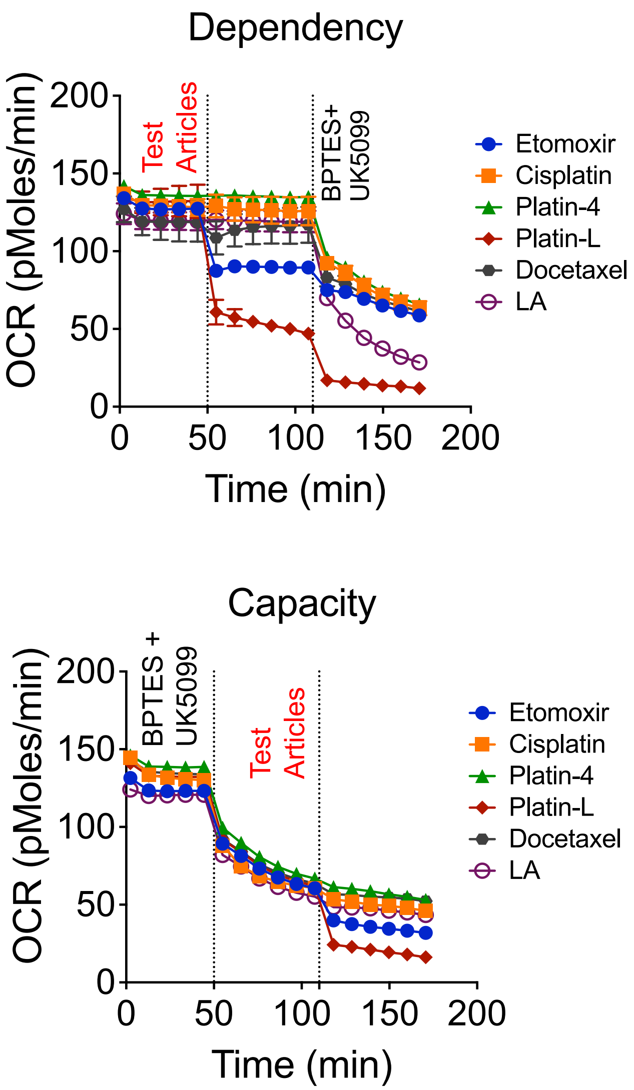

Fig. S10. Modulation of FAO pathway in PC3 cells. Real time oxygen consumption was monitored in response to addition of etomoxir, Platin-L, Platin-4, cisplatin, lauric acid (LA), or Docetaxel in PC3 cells, followed by the simultaneous injection of UK5099 plus BPTES to evaluate the effect of the test articles on FAO. Concentration of test articles was 100 μM. Etomoxir, UK5099 and BPTES concentration were constant at 40 μM, 20 μM and 30 μM respectively.

(A)

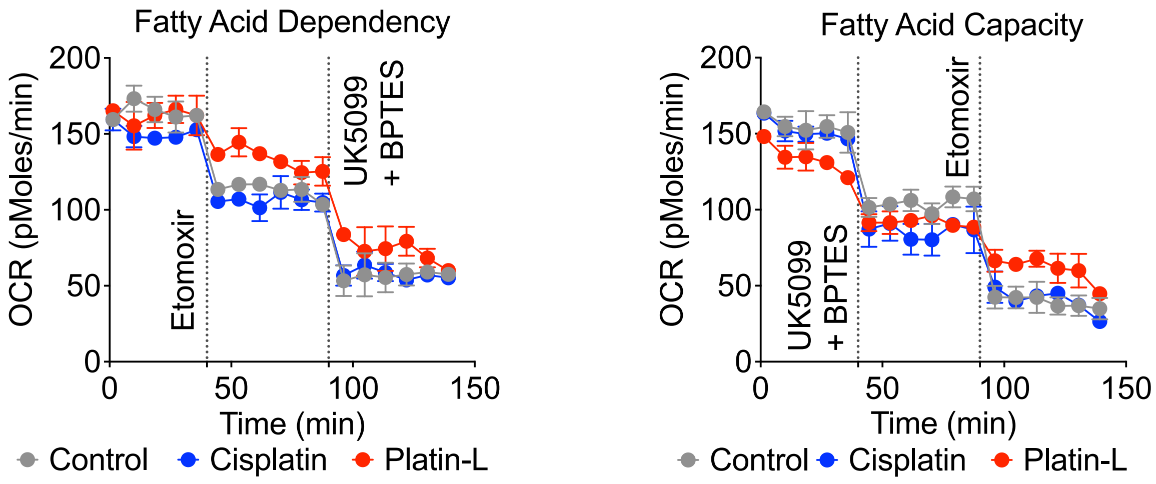

(B)

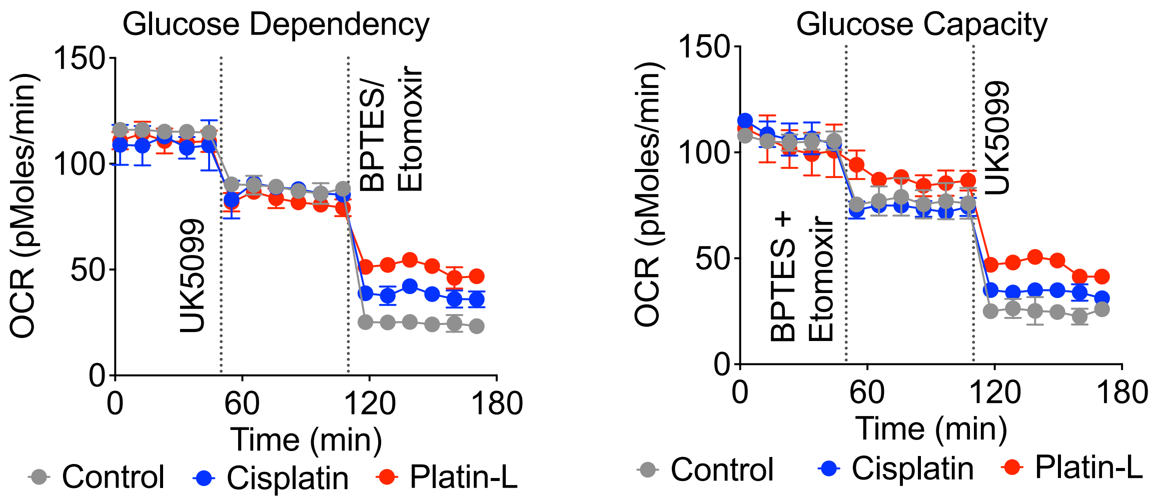

Fig. S11. Effects of Platin-L on (A) fatty acid dependency and (B) glucose dependency of LNCaP cells. Cells were treated with cisplatin or Platin-L (both at 10 μM) for 24h, Followed by monitoring the real time oxygen consumption in a seahorse Fuel Flex assay for the glucose dependency or fatty acid dependency.

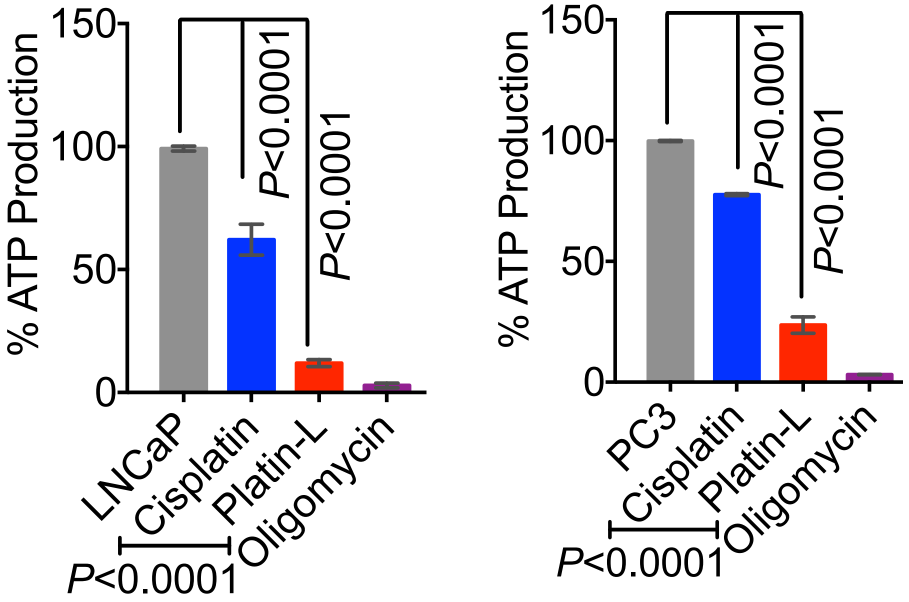

Fig. S12. ATP production in PC3 and LNCaP cells was evaluated in the presence of cisplatin or Platin-L (10 μM each), or Oligomycin (10 μM) as a positive control which is an ATP synthase inhibitor. Treatment was carried out for 24 h followed by quantification of ATP production using CellTiter-Glo Reagent.

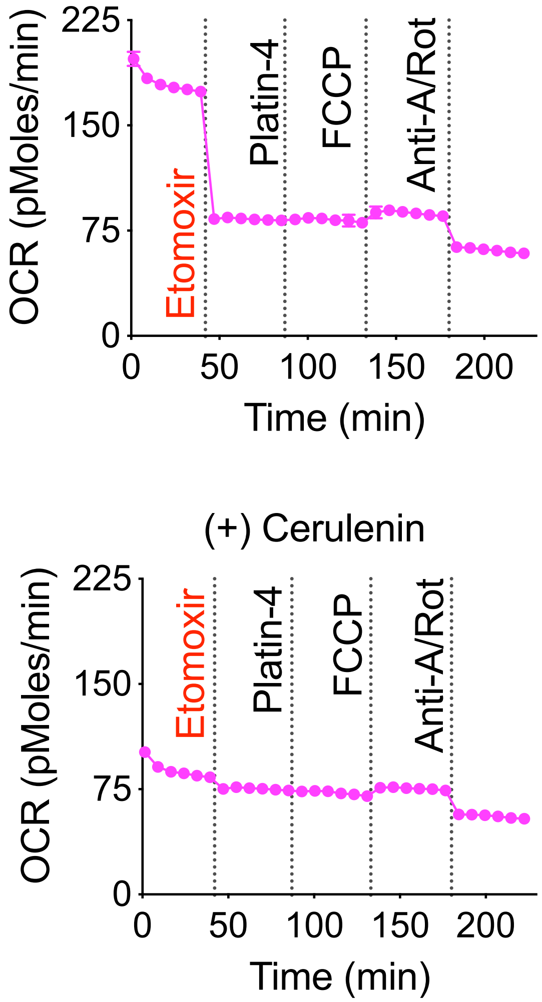

Fig. S13. LNCaP cells (top) without cerulenin pretreatment and (bottom) with cerulenin pretreatment followed by etomoxir or Platin-4 to check FAO inhibition efficacy. 20,000 cells were plated per well in a seahorse assay 96-well plate and grown overnight followed by treatment with cerulenin at 20 μM for 24 h. Final concentration of articles were: etomoxir – 40 μM, Platin-4 – 100 μM, FCCP – 1 μM, and Antimycin-a + Rotenone – 1 μM each.

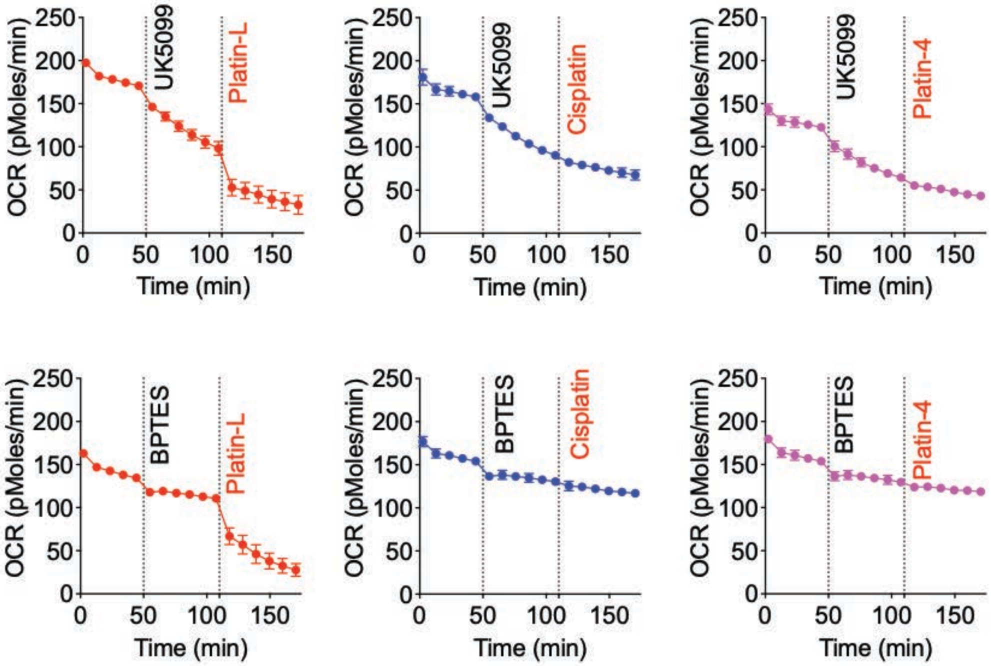

Fig. S14. The effect of cisplatin, Platin-L and Platin-4 to modulate oxygen consumption after shutting down the glucose or glutamine metabolic pathways was studied using a Seahorse extracellular flux analyzer. Following injection of glucose inhibitor UK5099 (20 μM) or glutamine inhibitor (30 μM), cisplatin, Platin-L or Platin-4 (100 μM) was injected, and the oxygen consumption was monitored.

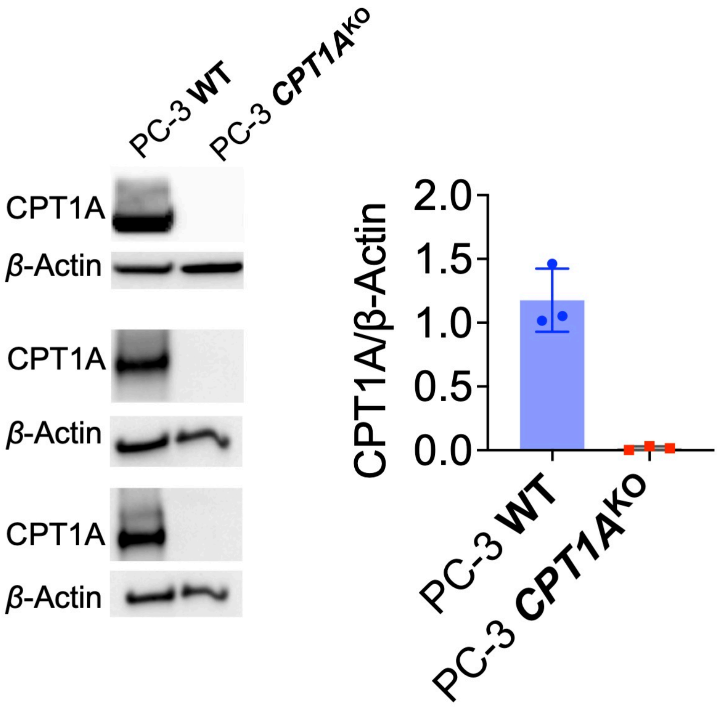

Fig. S15. Confirmation of *CPT1A* knockout in PC3 cells by western blot analysis. Cells were grown to confluency and then the cells lysates were prepared and analyzed for the presence of CPT1A. Each pair of CPT1A and *β*-actin represent an independent experiment. Expression of CPT1A was quantified with respect to *β*-actin on the right.

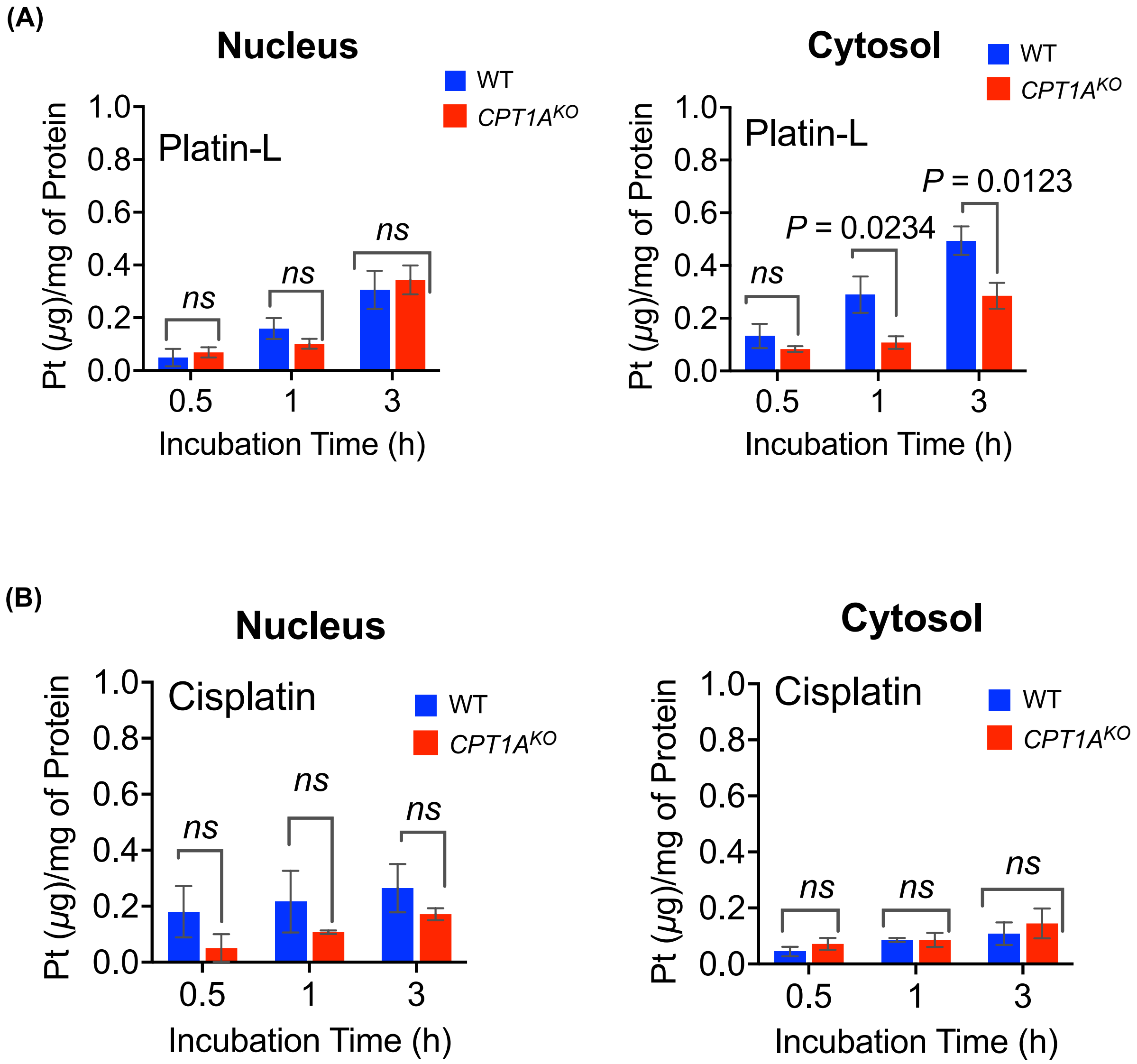

Fig. S16. Amount of Pt present in the nuclear and cytosolic fractions of PC-3 WT and CPT1A^{KO} PC-3 cells treated with Platin-L or Cisplatin. Cells were treated with Platin-L or cisplatin at 50 μg/mL for 0.5, 1, or 3 h followed by subcellular fractionation carried out with mitochondrial and nuclear isolation kits from Thermo Fisher. The isolated fractions were then analyzed for platinum content using ICP-MS.

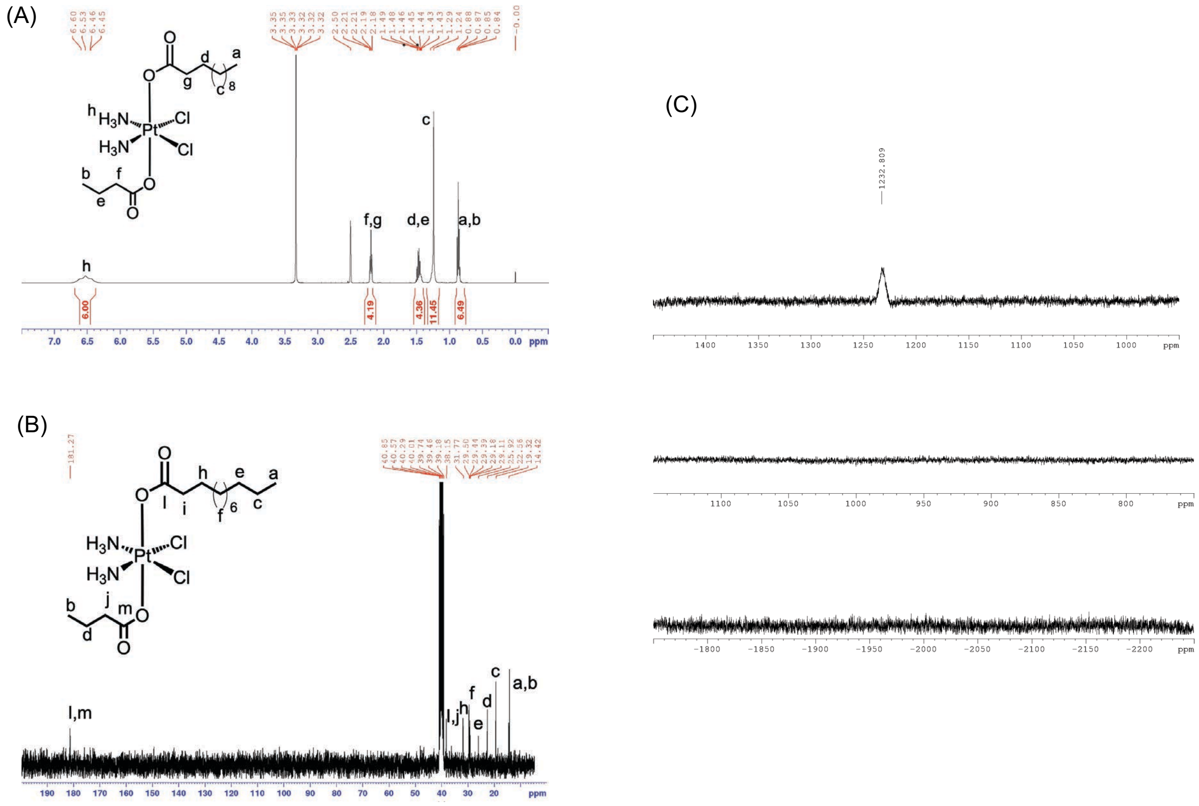

Fig. S17. (A) ^{1}H NMR of Platin-L-4 ($CDCl_3$, 400 MHz) δ 0.87 (t, 6H), 1.24 (s, 12H), 1.44 (m, 4H), 2.18 (m, 4H). 6.53 (m, 6H) (B) ^{13}C NMR spectrum of Platin-L-4. (C) ^{195}Pt NMR of Platin-L-4. Top: Region of spectrum corresponding to Pt(IV) compounds with two axial ligands. Middle: Region corresponding to Pt(IV) compounds with one axial ligand. Bottom: Region corresponding to Pt(II) compounds.

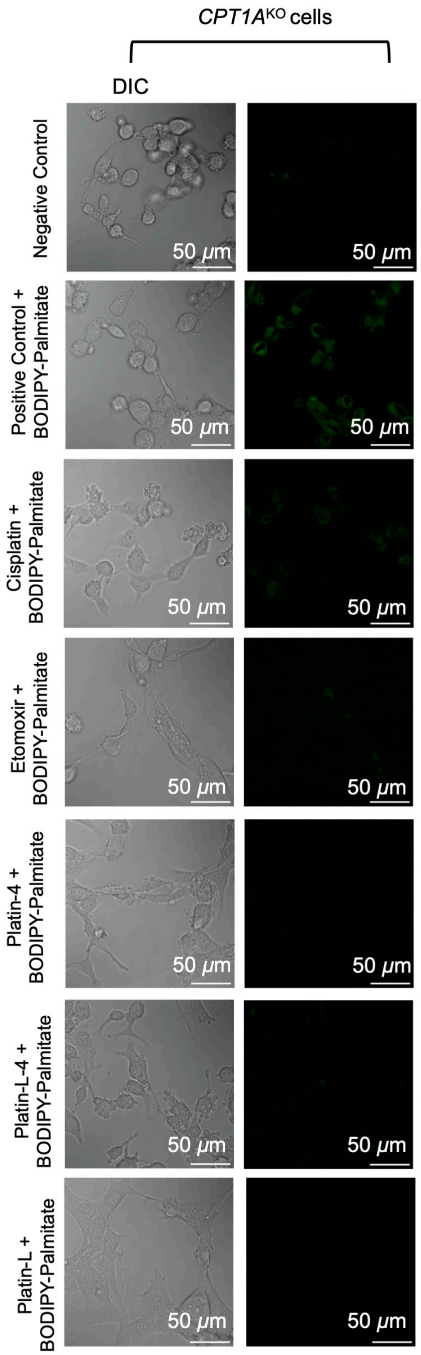

Fig. S18. Uptake of BODIPY-Palmitate in *CPT1A*^{KO} cells. Laser: 488 nm; Laser Transmissivity: 0.04%; PMT Voltage: 655 V

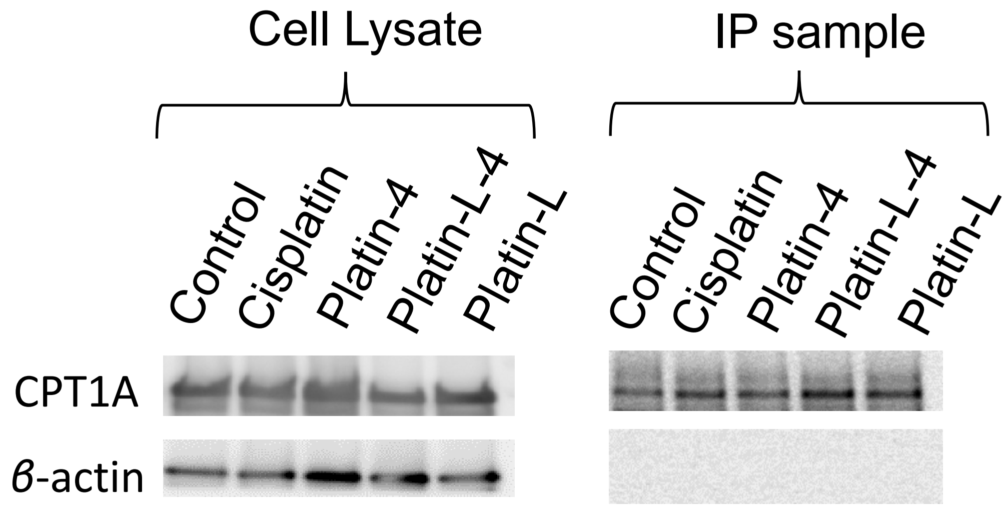

Fig. S19. Confirmation of CPT1A isolation through immunoprecipitation after treatment of PC-3 cells with cisplatin, Platin-4, Platin-L-4, or Platin-L.

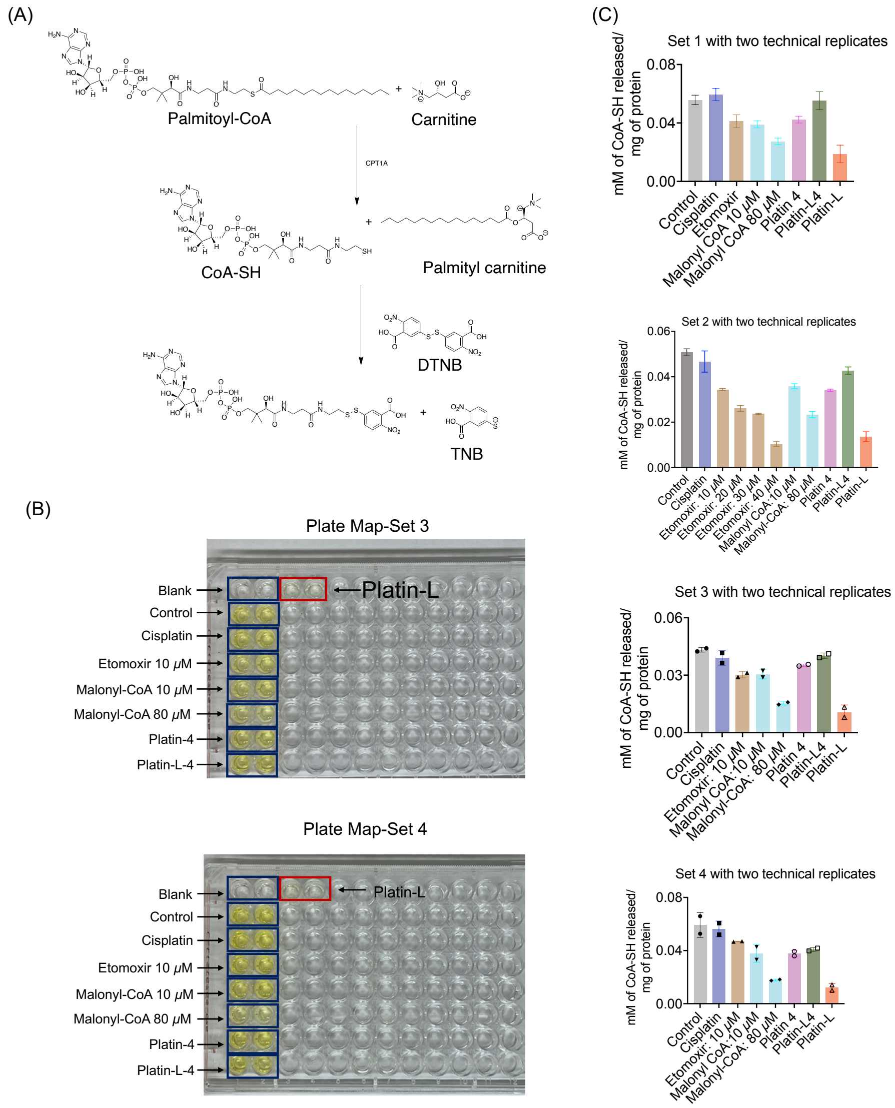

Fig. S20. CPT1 activity assay. (A) Chemical scheme of the reaction occurring in the well. Activity of CPT1A governs the release of CoA-SH from palmitoyl-CoA, which in turn reacts with DTNB to release TNB which has a yellow color. (B) Plate maps of two biological replicates of the assay with the difference in color, corresponding to the activity of CPT1A in the cell lysates after treatment. (C) CPT1A activity from individual biological replicates represented as mM of CoA-SH released normalized by the amount of protein present. Treatment was at 10 μM for 3 h unless otherwise noted. Absorbance of TNB was measured on a plate reader at 405 nm wavelength.

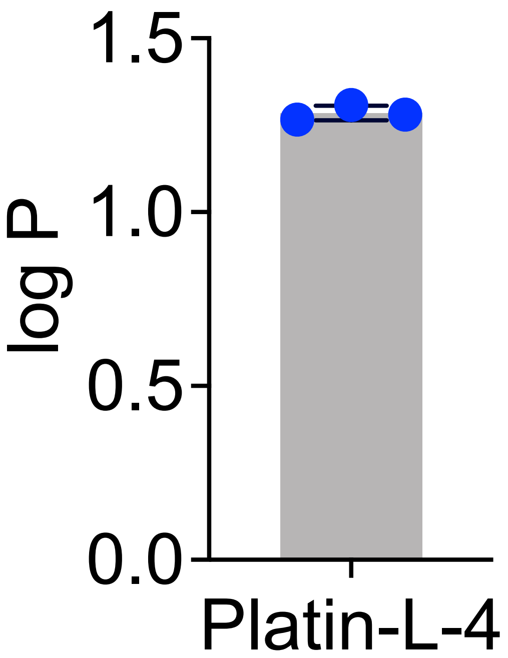

Fig. S21. Lipophilicity of Platin-L-4. Plot of the logP value of Platin-L-4. LogP value of Platin-L-4 was calculated by creating a solution of 500 μM Platin-L-4 in phosphate buffered saline (PBS) saturated octanol, followed by mixing with equal volume of PBS for 16 h. The layers were separated, and Pt was quantified in the separate layers using ICP-MS. LogP value was calculated using the equation LogP = Log([Platin-L-4_{Oct}]/[Platin-L-4_{PBS}]).

Set I

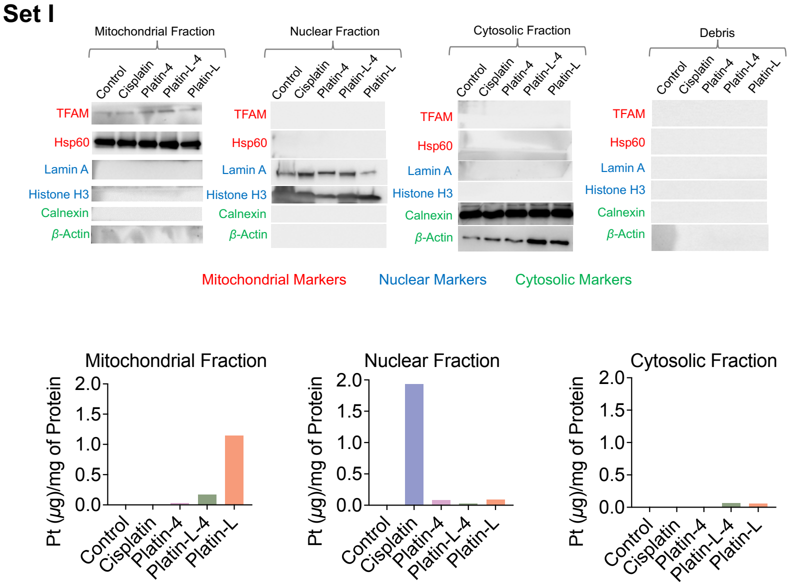

Set II

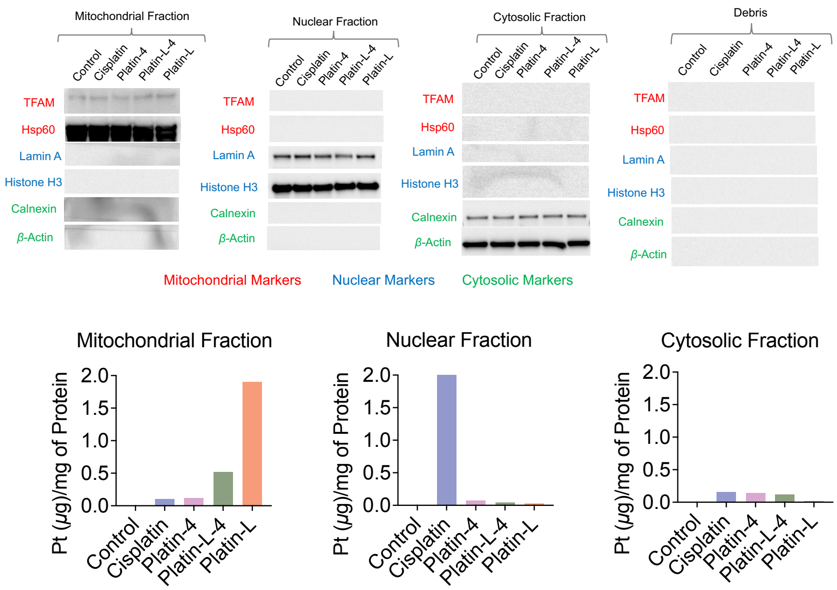

Fig. S22. Subcellular localization of different compounds in PC3 cells. The cells treated with various platinum compounds (10 μM for 3 h) and then subcellular fractions were isolated. The purity of the fractions was analyzed through Western Blot (top: **set I** and bottom: **set II**). Subsequently, the fractions were analyzed for platinum content using ICP-MS and normalized for protein content using BCA assay (top: **set I** and bottom: **set II**). Data from two biological replicates are shown.

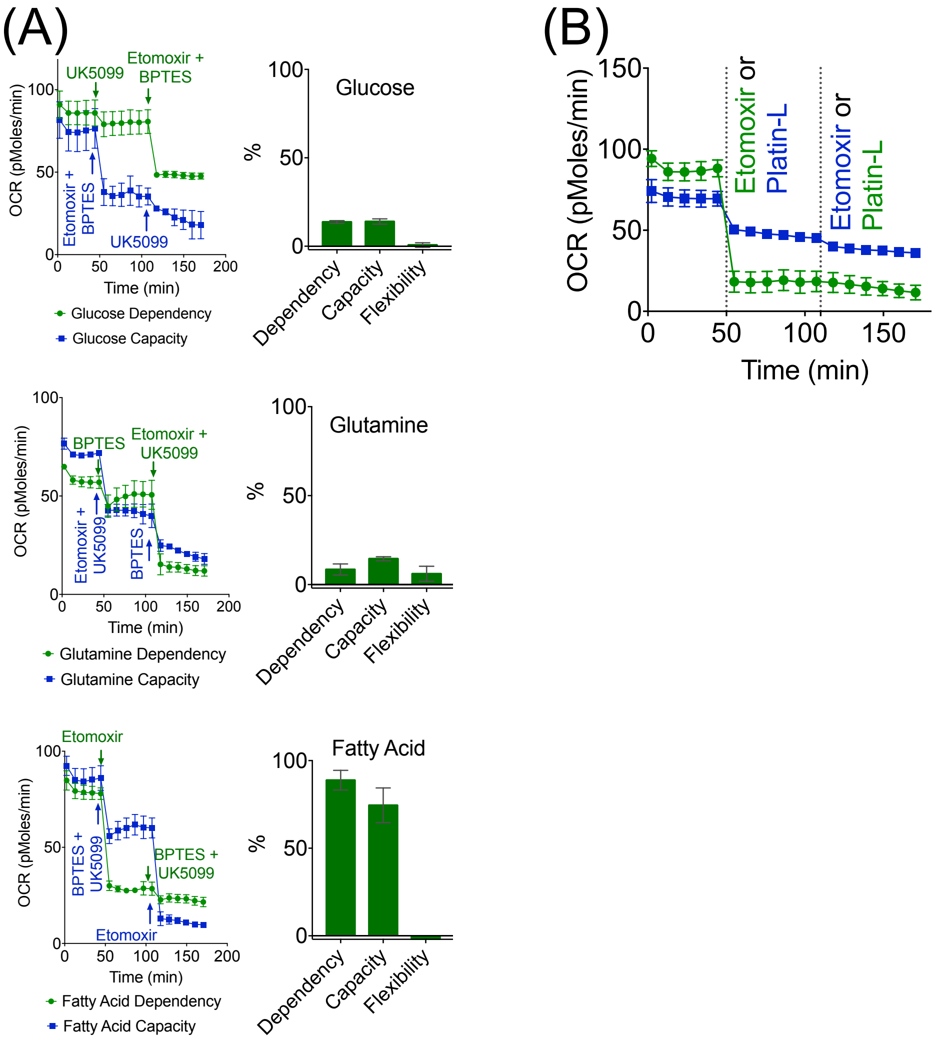

Fig. S23. (A) Substrate utilization by HepG2 cells using fuel flex assay. Dependency and capacity of HepG2 cells on glucose, glutamine and fatty acids were determined. (B) FAO inhibition in HepG2 cells by Platin-L or etomoxir. [Platin-L], 100 μM; [etomoxir], 40 μM.

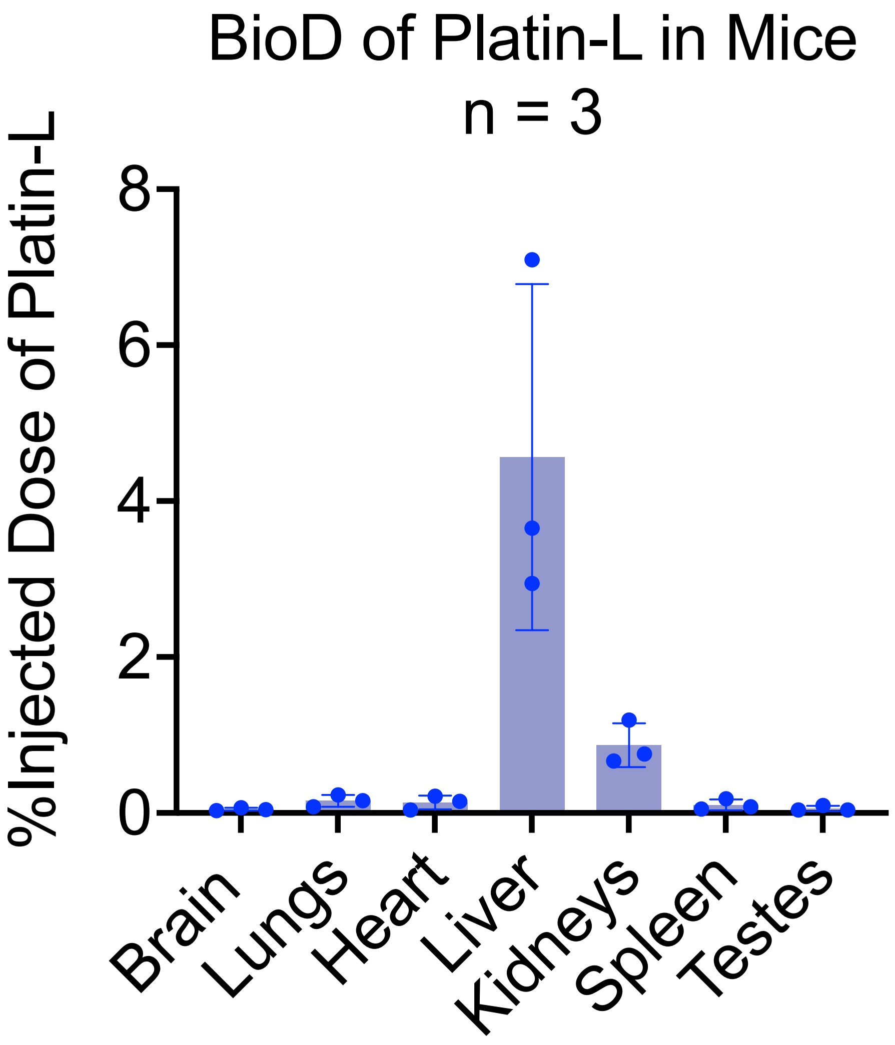

Fig. S24. BioD of Platin-L in BALB/c albino mice 24 h post administration *via* intravenous route, 1.2 mg/kg. Organs were dissolved in aqua regia and analyzed using ICP-MS for platinum content.

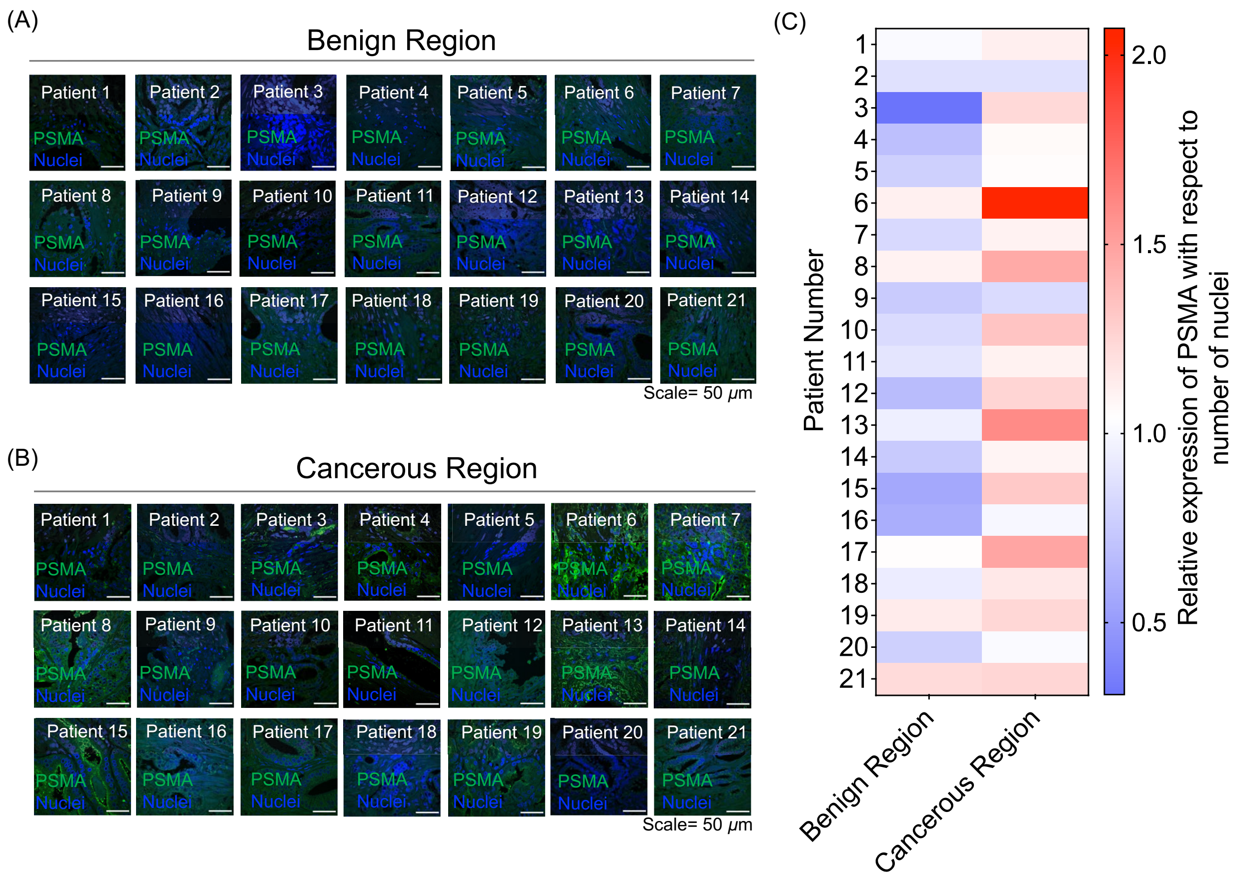

Fig. S25. Heterogeneity of PSMA expression in treatment naïve patient samples. Expression of PSMA in the tumor sections was analyzed through immunofluorescence in both benign (A) and cancerous (B) regions of the tissue. (C) Expression of PSMA in with respect to the number of nuclei was calculated and expressed as a heat map. Five images were taken for each sample and the average expression of PSMA was calculated with respect to the number of nuclei present in the image. Quantification was carried out using ImageJ software.

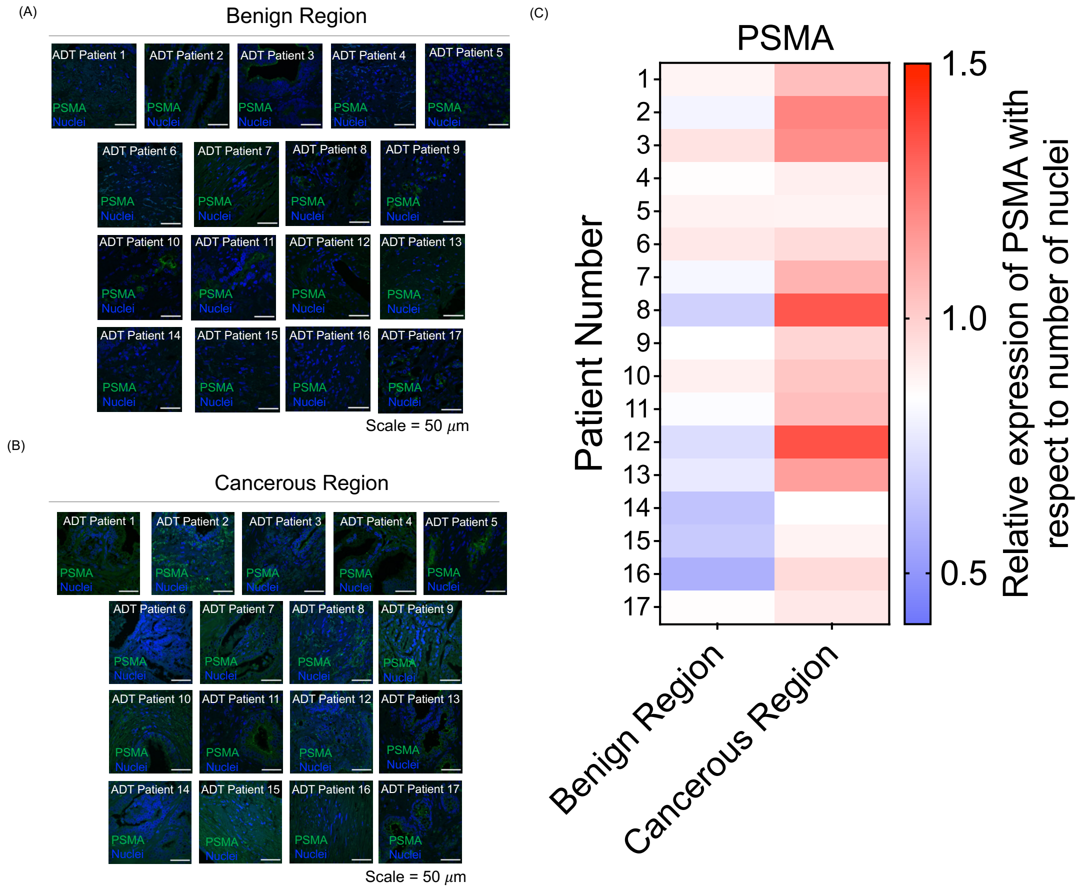

Fig. S26. Heterogeneity of PSMA expression in ADT treated patient samples. Expression of PSMA in the tumor sections was analyzed through immunofluorescence in both benign (A) and cancerous (B) regions of the tissue. (C) Expression of PSMA in with respect to the number of nuclei was calculated and expressed as a heat map. Five images were taken for each sample and the average expression of PSMA was calculated with respect to the number of nuclei present in the image. Quantification was carried out using ImageJ software.

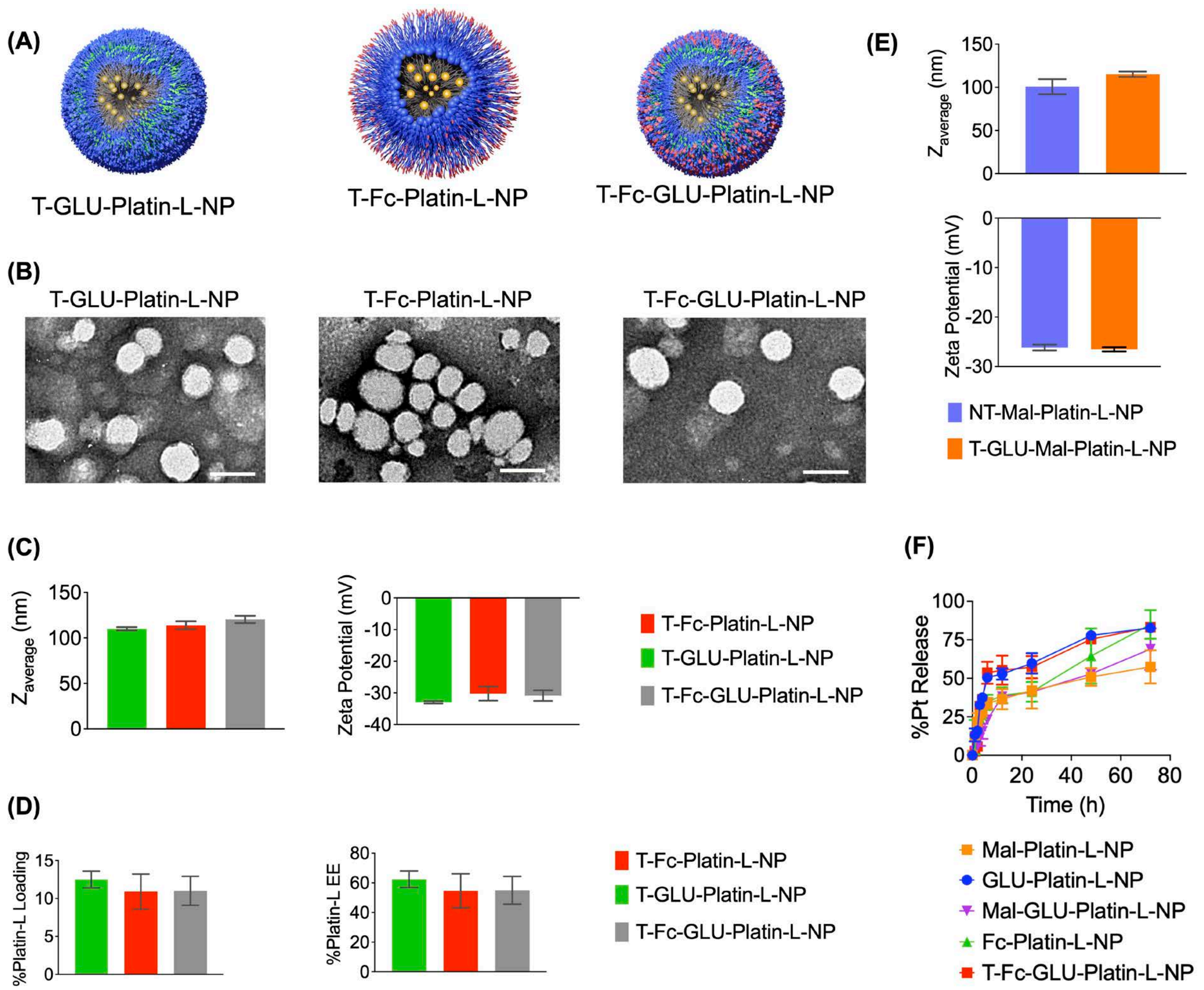

Fig. S27. Nanoparticle characterization of nanoparticles with different polymer composition. (A) Graphical representation, (B) transmission electron microscopy images; scale bar: 100 nm, (C) average diameter and zeta potential from repeated independent measurements, (D) average % Platin-L loading and % Platin-L Encapsulation Efficiency of T-GLU-Platin-L-NP, T-Fc-Platin-L-NP and T-Fc-GLU-Platin-L-NP. (E) Comparison of size and zeta potential between NT-Mal-Platin-L-NP and T-GLU-Mal-Platin-L-NP. (F) Release of Platin-L over time from NPs with different polymer composition. NPs were incubated in 1X PBS in dialysis tubes at 37 ºC and the amount of platinum was quantified at predetermined time points.

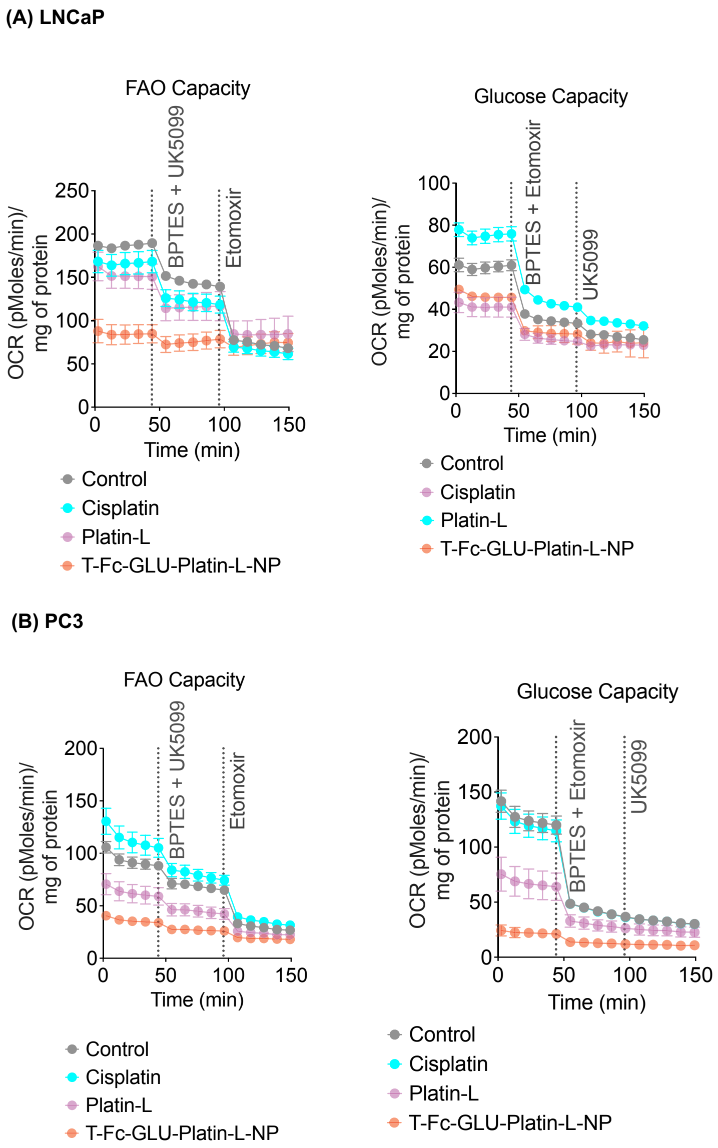

Fig. S28: Fatty acid capacity and glucose capacity of cisplatin, Platin-L and T-Fc-GLU-Platin-L-NP in LNCaP and PC3 cells.

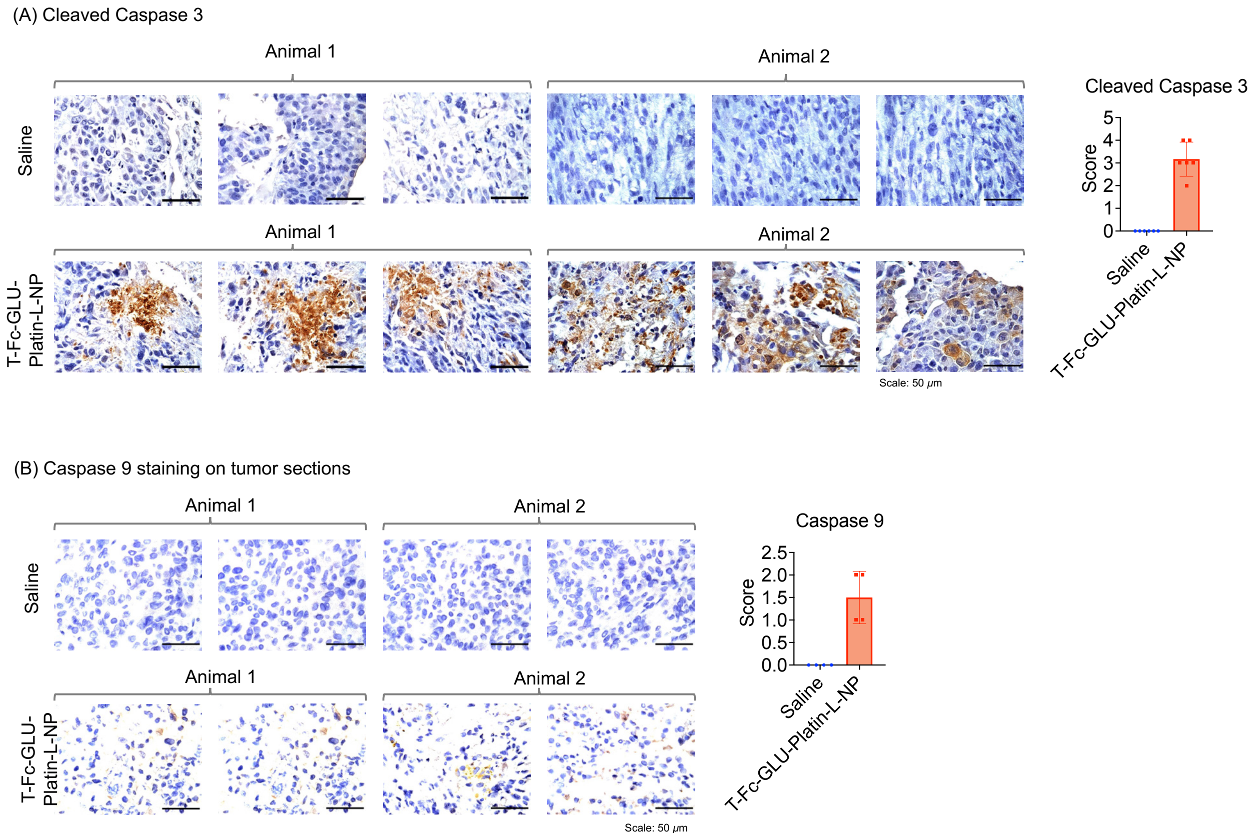

Fig S29. Tumor sections with IHC staining for (A) Cleaved caspase 3 and (B) caspase 9 from different animals. Multiple images from different regions of tissue from different animals were used for scoring.

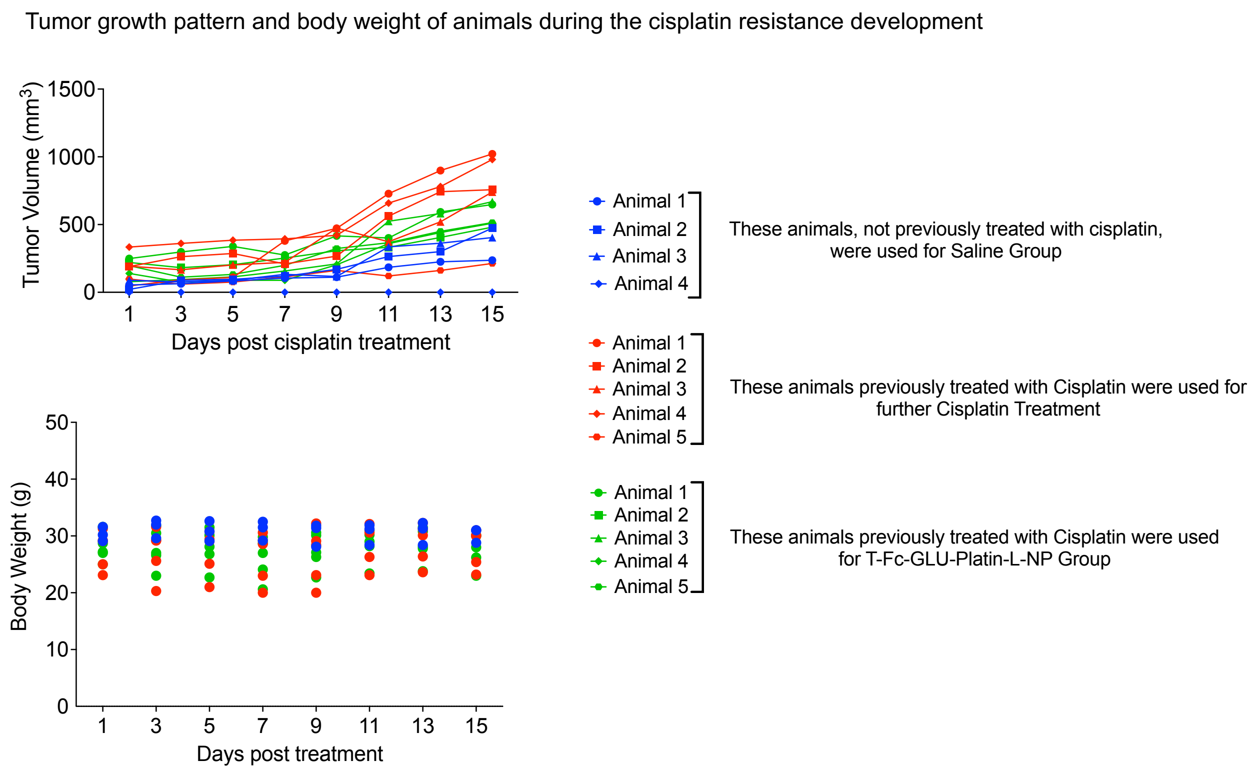

Fig. S30. Tumor volume and body weight of mice in cisplatin resistance LNCaP Xenograft mouse model. Other than the mice in the saline group, all mice were administered 3 mg/kg cisplatin via tail vein. Mice were randomly sorted into cisplatin or T-Fc-GLU-Platin-L-NP treatment groups after resistance was established. One mouse from the saline group did not develop a tumor until later into the study.

(A)

Dose No.	$Z_{average}$ (nm)	PDI	Zeta Potential (mV)	%Platin-L Loading	%Fc Conjugation
1	104±2.255	0.151	-30.8±1.1	12.67	27.2
2	120.1± 5.56	0.204	-29.2±1.3	11.1	25.6
3	103±2.566	0.143	-25.4±1.1	8.43	29.3
4	113.5±2.193	0.185	-27.2±0.8	7.9	25.6
5	88.96±1.804	0.17	-30.1±1.2	11.31	27.6
6	90.23±2.107	0.155	-28.2±1.1	12.36	29.1
7	90.61±2.610	0.179	-27.7±1.6	10.96	37.5
8	108±3.342	0.178	-23.4±1.5	9.12	29.1
9	129.3±2.193	0.298	-24.8±1.1	8.42	38.7
10	92.7±1.804	0.18	-28.2±1.2	14.72	39.1
11	99.2±0.228	0.21	-23.2±1.1	10.89	32.5
12	108.2±0.503	0.184	-26.2±0.9	10.23	25.6
13	120.1±6.96	0.204	-22.3±2.1	11.28	35.3
14	107.3±1.552	0.278	-28.4±1.0	12.11	33.7
15	90.6±1.201	0.179	-29.2±1.3	9.7	32.1
16	106.7±0.503	0.21	-29.1±1.1	8.7	37.1
17	102±3.064	0.177	-30.5±1.0	14.2	29.4
18	113.5±2.972	0.185	-25.6±0.8	13.2	24.7
19	119.4±4.91	0.272	-27.7±0.7	14.2	41.2
20	98.43±1.18	0.18	-27.2±0.8	14.9	34.6
21	113.2±1.206	0.176	-30.4±1.8	12.94	28.3
22	104±4.43	0.175	-29.9±1.5	10.66	29
23	110±0.864	0.174	-26.3±0.8	10.41	32.7
24	121±3.47	0.272	-27.3±1.2	10.75	37.6
25	103.2±1.978	0.198	-24.8±1.2	7.03	38.9
26	102.1±3.084	0.21	-26.4±1.1	11.07	25.6
27	116±3.921	0.182	-31.6±1.3	10.01	29.1
28	88.6±2.872	0.17	-28.6±0.9	9.72	27.4
29	117±1.697	0.192	-29.2±1.1	14.11	32.4
30	129.3±3.336	0.298	-28.4±1.2	12.19	37.6
31	113.5±4.911	0.185	-26.5±0.9	10.72	33.7
32	88.9±1.109	0.17	-30.8±1.0	10.64	34.7

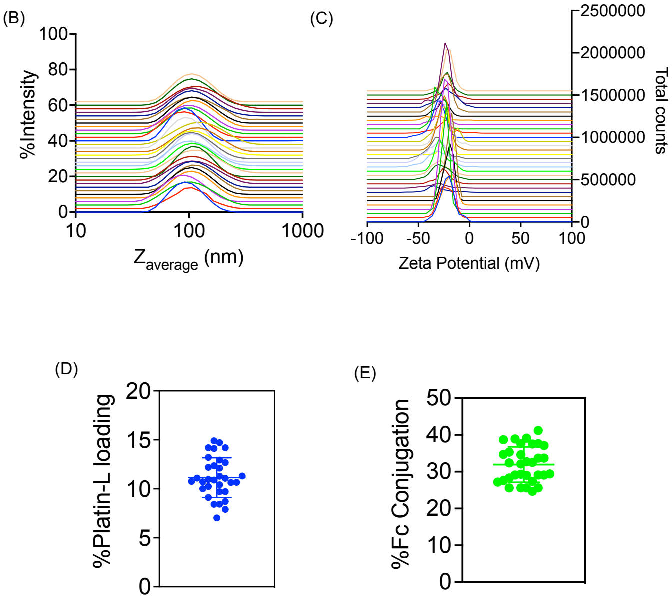

Fig. S31. Nanoparticle characterization data for NPs used in cisplatin resistant LNCaP xenograft model. (**A**) table of the physical characteristics of each dose of NP, including the Platin-L loading and %Fc Conjugation to the NP. (**B**) Overlay of the size and (**C**) Zeta potential graphs from each dose. (**D**) Percent loading of Platin-L for each batch of NP, and (**E**) percent Fc conjugation for each batch of NP used during the course of the study.

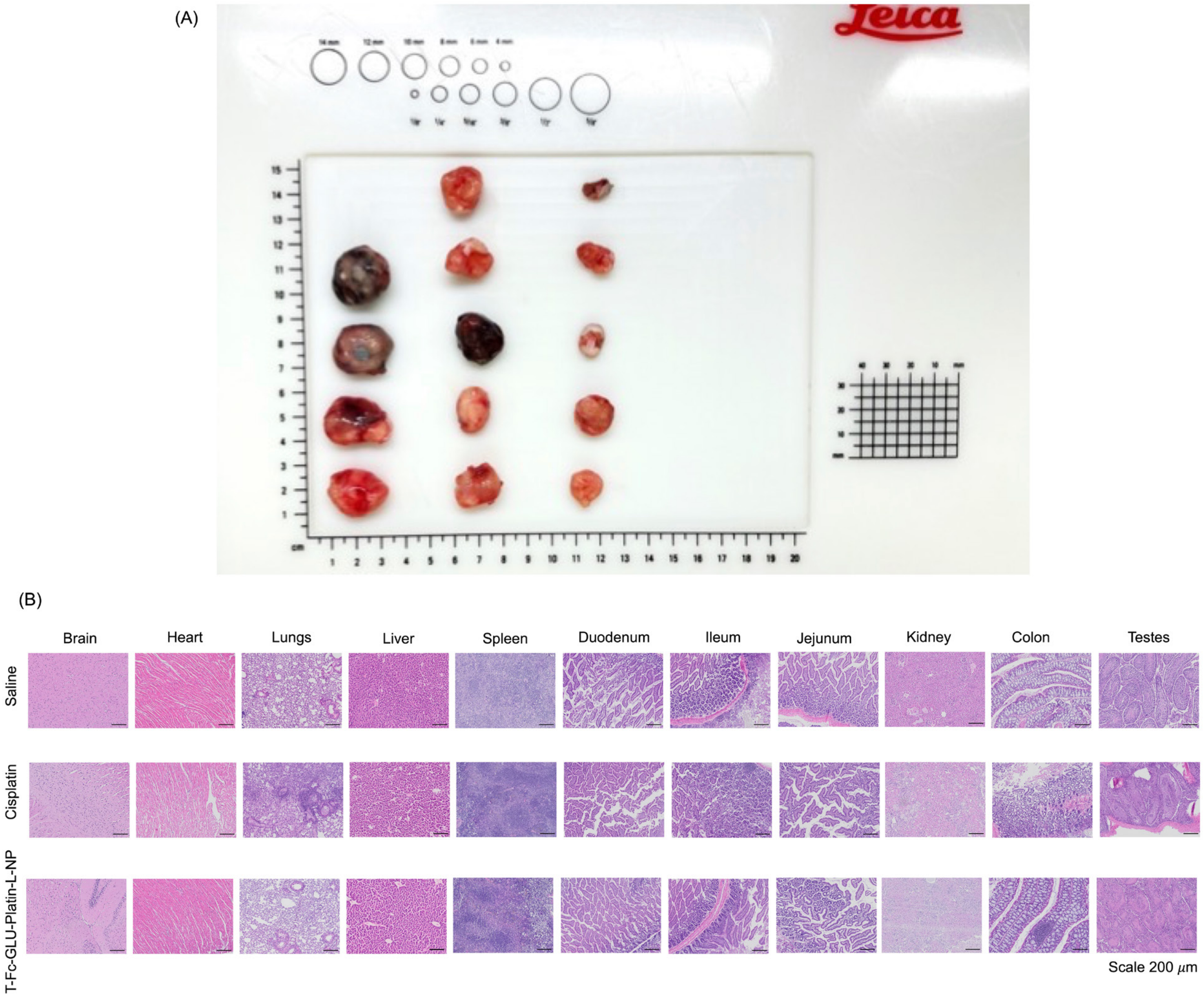

Fig. S32. (**A**) Full image of the excised tumors from the cisplatin resistant Xenograft model on a grossing board with scales. (**B**) H&E images of all organs from representative mice treated with Saline, cisplatin, or T-Fc-GLU-Platin-L-NP.

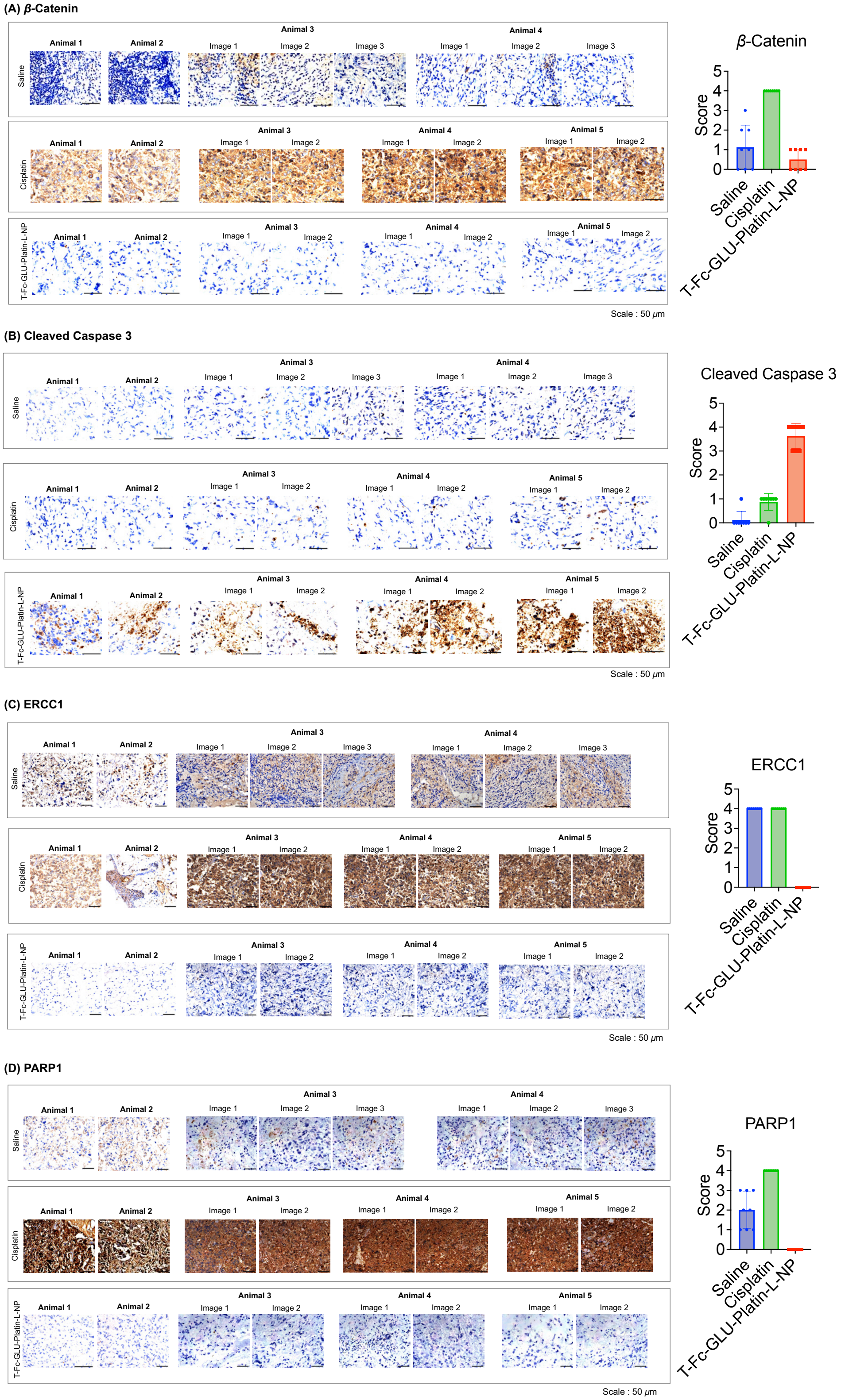

Fig. S33. Immunohistological staining of (A) beta catenin, (B) Cleaved caspase 3, (C) ERCC1, (D) PARP1 in tumor sections from different animals along with multiple images used for scoring.

References

1. Marrache, S.; Dhar, S., Engineering of blended nanoparticle platform for delivery of mitochondria-acting therapeutics. *Proc Natl Acad Sci U S A* **2012,** *109* (40), 16288-93.
2. Kalathil, A. A.; Kumar, A.; Banik, B.; Ruiter, T. A.; Pathak, R. K.; Dhar, S., New formulation of old aspirin for better delivery. *Chem Commun* **2016,** *52* (1), 140-3.
3. Marrache, S.; Choi, J. H.; Tundup, S.; Zaver, D.; Harn, D. A.; Dhar, S., Immune stimulating photoactive hybrid nanoparticles for metastatic breast cancer. *Integr Biol* **2013,** *5* (1), 215-23.
4. Marrache, S.; Dhar, S., Biodegradable synthetic high-density lipoprotein nanoparticles for atherosclerosis. *Proc Natl Acad Sci U S A* **2013,** *110* (23), 9445-50.
5. Marrache, S.; Tundup, S.; Harn, D. A.; Dhar, S., Ex vivo programming of dendritic cells by mitochondria-targeted nanoparticles to produce interferon-gamma for cancer immunotherapy. *ACS Nano* **2013,** *7* (8), 7392-402.
6. Pathak, R. K.; Marrache, S.; Choi, J. H.; Berding, T. B.; Dhar, S., The prodrug Platin-A: simultaneous release of cisplatin and aspirin. *Angew Chem Int Ed Engl* **2014,** *53* (7), 1963-7.
7. Pathak, R. K.; Marrache, S.; Harn, D. A.; Dhar, S., Mito-DCA: A Mitochondria Targeted Molecular Scaffold for Efficacious Delivery of Metabolic Modulator Dichloroacetate. *ACS Chem Biol* **2014,** *9* (5), 1178-87.
8. Pathak, R. K.; Basu, U.; Ahmad, A.; Sarkar, S.; Kumar, A.; Surnar, B.; Ansari, S.; Wilczek, K.; Ivan, M. E.; Marples, B.; Kolishetti, N.; and Dhar, S. "A designer bow-tie combination therapeutic platform: An approach to resistant cancer treatment by simultaneous delivery of cytotoxic and anti-inflammatory agents and radiation" *Biomaterials*, **2018**, *187*, 117-129.
9. Marrache, S.; Pathak, R. K.; and Dhar, S. Detouring of cisplatin to access mitochondrial genome for overcoming resistance *Proc. Natl. Acad. Sci. USA,* **2014***, 111,* 10444-10449*.*
10. Pathak, R. K.; McNitt, C. D.; Popik, V. V. and Dhar, S. Copper-free click-chemistry platform to functionalize cisplatin prodrugs *Chem. Eur. J.*, **2014**, *20*, 6861-6865.
11. Pathak, R. K. and Dhar, S. Unique use of alkylation for chemo-redox activity by a Pt(IV) prodrug" *Chem. Eur. J.,* **2016***, 22,* 3029-3036*.*
